# A MAFG~MITF complex drives melanoma phenotype switching and progression

**DOI:** 10.1038/s41467-026-73291-x

**Published:** 2026-05-21

**Authors:** Olga Vera, Michael Martinez, Zulaida Soto-Vargas, Kaizhen Wang, Xiaonan Xu, Nicol Mecozzi, Harini Murikipudi, Sara Ruiz-Buceta, Manon Chadourne, Benjamin Posorske, Ariana Angarita, Ilah Bok, Juan D. Ulloa Arrieta, Qian Liu, Yumi Kim, Jane L. Messina, Kenneth Y. Tsai, Michael B. Major, Eric K. Lau, Xiaoqing Yu, Inmaculada Ibanez de Caceres, Florian A. Karreth

**Affiliations:** 1 Department of Molecular Oncology, H. Lee Moffitt Cancer Center and Research Institute, Tampa, Florida USA; 2 Experimental Therapeutics and Biomarkers in Cancer, IdiPAZ, Madrid, Spain; 3 Cancer Epigenetics Laboratory, INGEMM, La Paz University Hospital, Madrid, Spain; 4 Cancer Biology PhD Program, University of South Florida, Tampa, FL USA; 5 Department of Cell Biology and Physiology, Washington University, St. Louis, MO USA; 6 Department of Tumor Microenvironment and Metastasis, H. Lee Moffitt Cancer Center and Research Institute, Tampa, FL USA; 7 Department of Metabolism and Physiology, H. Lee Moffitt Cancer Center and Research Institute, Tampa, FL USA; 8 Department of Pathology, H. Lee Moffitt Cancer Center & Research Institute, Tampa, FL USA; 9 Division of Oncological Sciences, Knight Cancer Institute, Oregon Health & Science University, Portland, OR USA; 10 Department of Biostatistics and Bioinformatics, H. Lee Moffitt Cancer Center and Research Institute, Tampa, Florida USA; 11 Present Address: Inserm, Biology and Pathologies of melanocytes, team1, Equipe Labellisée Ligue 2025 and Equipe Labellisée ARC 2022, Centre Méditerranéen de Médecine Moléculaire, Nice, France; 12 Present Address: A.T. Still University, School of Medicine, 5850 E. Still Circle, Mesa, AZ USA

**Keywords:** Melanoma, Transcriptomics, Mechanisms of disease, Oncogenes, Cancer models

## Abstract

Phenotype switching, a key driver of melanoma progression and therapy resistance, is governed by the lineage transcription factor MITF. Here, we identify the small MAF family transcription factor MAFG as a critical regulator of MITF activity and melanoma cell state plasticity. MAFG expression is frequently elevated in melanoma and correlates with poor patient survival. Mechanistically, MAFG binds MITF and redirects its genomic occupancy, thereby modulating transcriptional programs governed by MITF. Genetic perturbation studies in vitro and in vivo show that MAFG promotes a dedifferentiated cell state and accelerates melanoma progression through its direct interaction with MITF. Moreover, MAFG is required for melanoma cell proliferation and for the transition from nevi to melanoma in genetic mouse models. Together, these findings demonstrate that the MAFG~MITF complex orchestrates phenotype switching and tumor progression, uncovering an unrecognized mechanism of MITF regulation in melanoma.

## Abstract (web summary)

Phenotype switching is a key driver of melanoma progression and therapy resistance. Here, the authors identify the small MAF family transcription factor MAFG as a critical regulator of microphthalmia-associated transcription factor (MITF) activity and melanoma cell state plasticity through binding MITF to redirect its genomic occupancy and modulate its transcriptional programming.

## Introduction

Melanoma is an aggressive cancer that affected 331,000 people in 2022, with 60,000 patients succumbing to the disease worldwide^1,2^. The incidence of melanoma has steadily risen over the past three decades, with 350,000 new cases and over 70,000 deaths projected for 2025^2^. While resection of early-stage tumors is often curative, metastatic melanoma remains a highly lethal disease. While targeted or immune therapies have improved this dire clinical outlook, they are limited in their efficacy by inherent or acquired resistance to these treatments. The plasticity of melanoma cells is critically associated with the development of resistance, enabling phenotype switching in response to environmental cues and stresses^3^. Moreover, melanoma cells can switch between invasive and proliferative states, which fuels the formation of metastasis^3,4^. This phenotype switch has been proposed to be mediated, at least in part, by the microphthalmia-associated transcription factor (MITF), a lineage transcription factor critical for melanocyte differentiation, proliferation, and survival^5,6^. However, the molecular mechanisms driving the phenotype switch in melanoma are incompletely understood.

Activating mutations in *BRAF* and *NRAS* occur in >80% of melanomas, and significant effort has been invested in exploiting these initiating genetic events and their downstream pathways for therapeutic intervention. Additional genetic events, such as loss of the tumor suppressors *CDKN2A* and *PTEN,* promote the early stages of melanoma progression. However, few mutations that specifically drive the advanced stages of melanoma progression and metastasis have been identified^7,8^. Instead, non-genetic mechanisms that influence gene expression programs have emerged as potent drivers of phenotype switching and thus melanoma progression and resistance^9,10^. Transcription factor dysregulation enables dynamic and reversible phenotypic adaptation, implicating it as a key mechanism of melanoma progression and resistance. Understanding the molecular mechanisms underlying melanoma plasticity is therefore critical for developing therapeutic strategies that can prevent or overcome treatment resistance.

We recently identified the transcription factor MAFG as a bona fide target of the miR-29 melanoma suppressor^11^. MAFG belongs to the small MAF family (sMAFs) of bZIP transcription factors that either homodimerize or heterodimerize with bZIP transcription factors of the Cap’N’Collar (CNC) or BACH families to activate or repress gene expression^12^. Despite their functions as obligate dimerization partners of transcription factors implicated in cancer, most notably the redox and metabolism master regulator NRF2^13^, little is known about the role of sMAFs in human malignancies. In melanoma, MAFG is stabilized by hyperactivation of the MAPK pathway through direct phosphorylation by ERK, facilitating the formation of an epigenetic silencing complex^14^. However, whether increased expression of MAFG promotes the formation of melanoma is unknown.

Here, we demonstrate that MAFG potently promotes melanoma progression through phenotype switching. Mechanistically, MAFG binds MITF and redirects its transcriptional program, driving dedifferentiation and malignant progression. This represents a previously unrecognized mode of MITF regulation with implications for melanoma therapy.

## Results

### MAFG overexpression elicits oncogenic effects in human melanocytes and melanoma cells

**Fig. 1:**
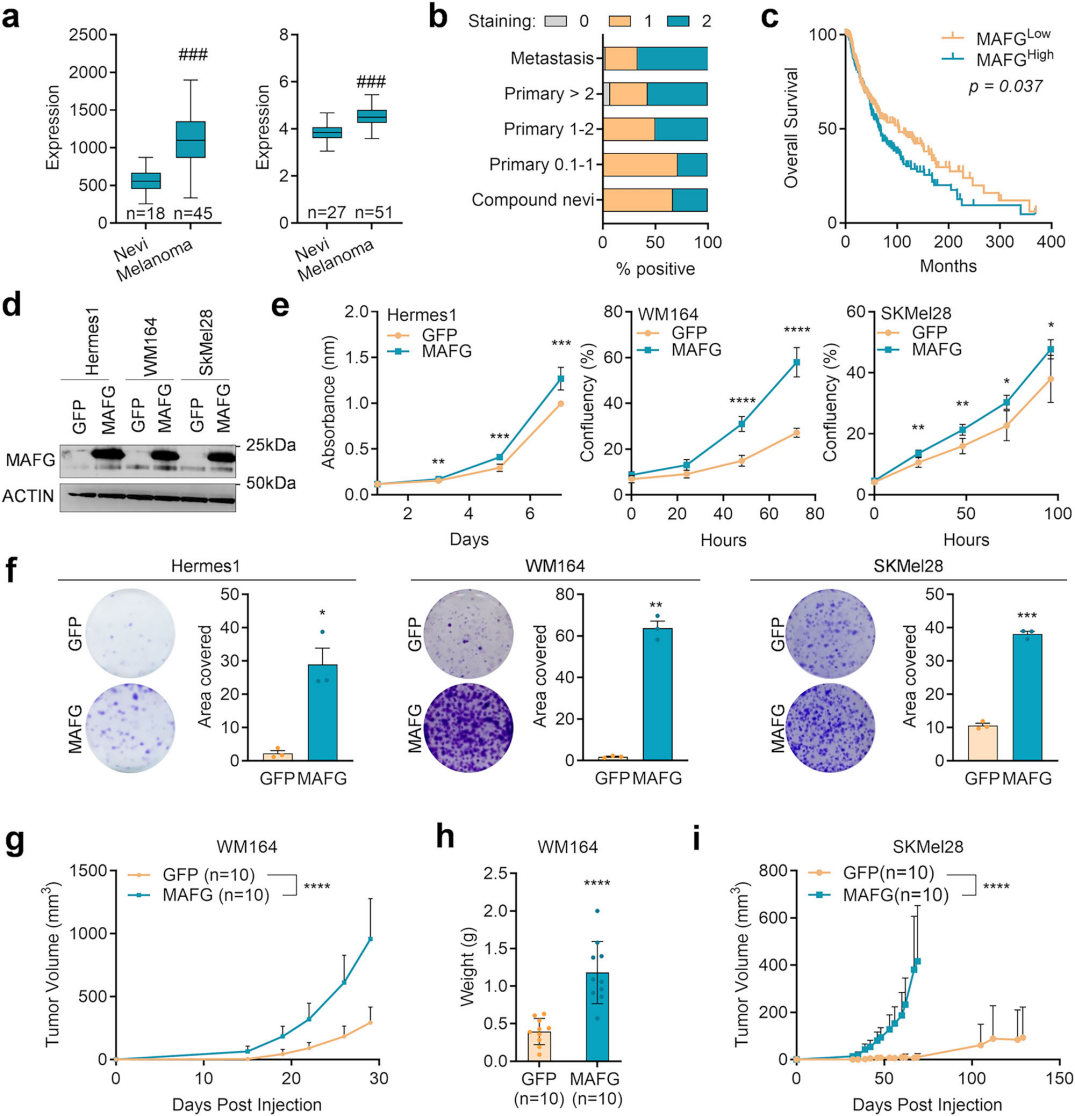
MAFG overexpression elicits oncogenic effects in melanocytes and melanoma cells. **a** MAFG expression in nevi and melanomas from GSE3189 (18 nevi, 45 melanomas) and GSE98394 (27 nevi, 51 melanomas). ### False discovery rate (FDR) < 0.001. **b** MAFG immunohistochemistry in nevi, primary melanomas, and metastatic melanomas from a tissue microarray scored from 0 (no staining) to 2 (high staining). **c** TCGA melanoma patient survival (PanCancer Atlas, *n* = 473) stratified by High vs Low MAFG expression (cutoff 1385.17 normalized reads). Survival differences were tested using two‑sided log‑rank (Mantel–Cox). **d** Western blot validation of MAFG overexpression in Hermes1, WM164 and SKMel28 cells; ectopic MAFG appears shifted due to the Myc‑DDK tag. The blot shown represents one out of three experiments with the same result. **e**, **f** Proliferation (**e**) and focus formation (**f**) assays of Hermes1 (left), WM164 (center), and SKMel28 (right) cells constitutively overexpressing MAFG or a GFP control. For the proliferation assay, *n* = 5 technical replicates from one out of two biological replicates are represented. Statistical significance was determined using a two‑sided Welch’s *t* test for each time point. *p*-values of each comparison at endpoint are as follows: Hermes1, *p* = 0.0024; WM164, *p *= 0.0002; SKMel28, *p* = 0.0453. Error bars represent mean ± s.d. For the focus formation assay, n = 3 technical replicates from one out of two biological replicates are represented. Statistical significance was determined using Welch’s corrected two-tailed *t* test for each time point. *p*-values of each comparison are as follows: Hermes1, *p* = 0.0291; WM164, p = 0.0027; SKMel28, *p* < 0.00001. Error bars represent mean + s.d. **g**, **h** Tumor volume (**g**) and tumor weight at endpoint (**h**) of xenograft assays performed with WM164 cells overexpressing MAFG (*n* = 10) or GFP (*n* = 10) control. Tumor volume was compared between groups at day 29 after subcutaneous injection by two-tailed Student’s *t* test (*p* < 0.0001). Tumor weight at endpoint was compared by two-tailed Student’s *t* test (*p* < 0.0001). Error bars represent mean + s.d. **i** Tumor volume of xenograft assays performed with SKMel28 cells overexpressing MAFG (*n* = 10) or GFP (*n* = 10) control. Tumor volume was compared between groups at day 69 after subcutaneous injection by two-tailed Student’s *t* test (*p* = 0.0004). Error bars represent mean + s.d.*p < 0.05; **p < 0.01; ***p < 0.001; ****p < 0.0001. Source data are provided as a Source Data file.

We previously reported that downregulation of the tumor suppressive miR-29 family increases MAFG levels in melanoma cells compared to melanocytes^11^. This prompted us to characterize the genomic and expression alterations of *MAFG* in melanoma. *MAFG* is amplified, gained and/or overexpressed in 32% of specimens in The Cancer Genome Atlas skin cutaneous melanoma (TCGA-SKCM) dataset (Supplementary Fig. 1a). Similarly, analysis of two RNA sequencing datasets (GSE3189 and GSE98394) revealed increased *MAFG* levels in melanoma compared to nevi (Fig. 1a). Immunohistochemistry on a tissue microarray containing human nevi, primary melanomas, and metastatic melanomas showed elevated MAFG staining intensity in advanced and metastatic specimens (Fig. 1b and Supplementary Fig. 1b). We also observed worse survival in melanoma patients with high *MAFG* expression in the TCGA-SKCM dataset (Fig. 1c). These findings indicate that *MAFG* is upregulated during melanoma progression and suggest an oncogenic role for MAFG. To determine whether MAFG possesses oncogenic activity in cells of the melanocytic lineage, we overexpressed MAFG in a human immortalized melanocyte (Hermes1) and two human melanoma cell lines (WM164, SKMel28) (Fig. 1d). MAFG overexpression in Hermes1, WM164 and SKMel28 cells significantly increased proliferation (Fig. 1e) and low-density focus formation (Fig. 1f). Moreover, MAFG overexpression markedly promoted tumor growth of WM164 and SKMel28 cells transplanted into the flanks of immunocompromised NSG mice (Fig. 1g–i). These results indicate that increased MAFG expression enhances the malignant behavior of human melanoma cells.

### MAFG overexpression promotes melanomagenesis in genetically engineered mouse models

**Fig. 2:**
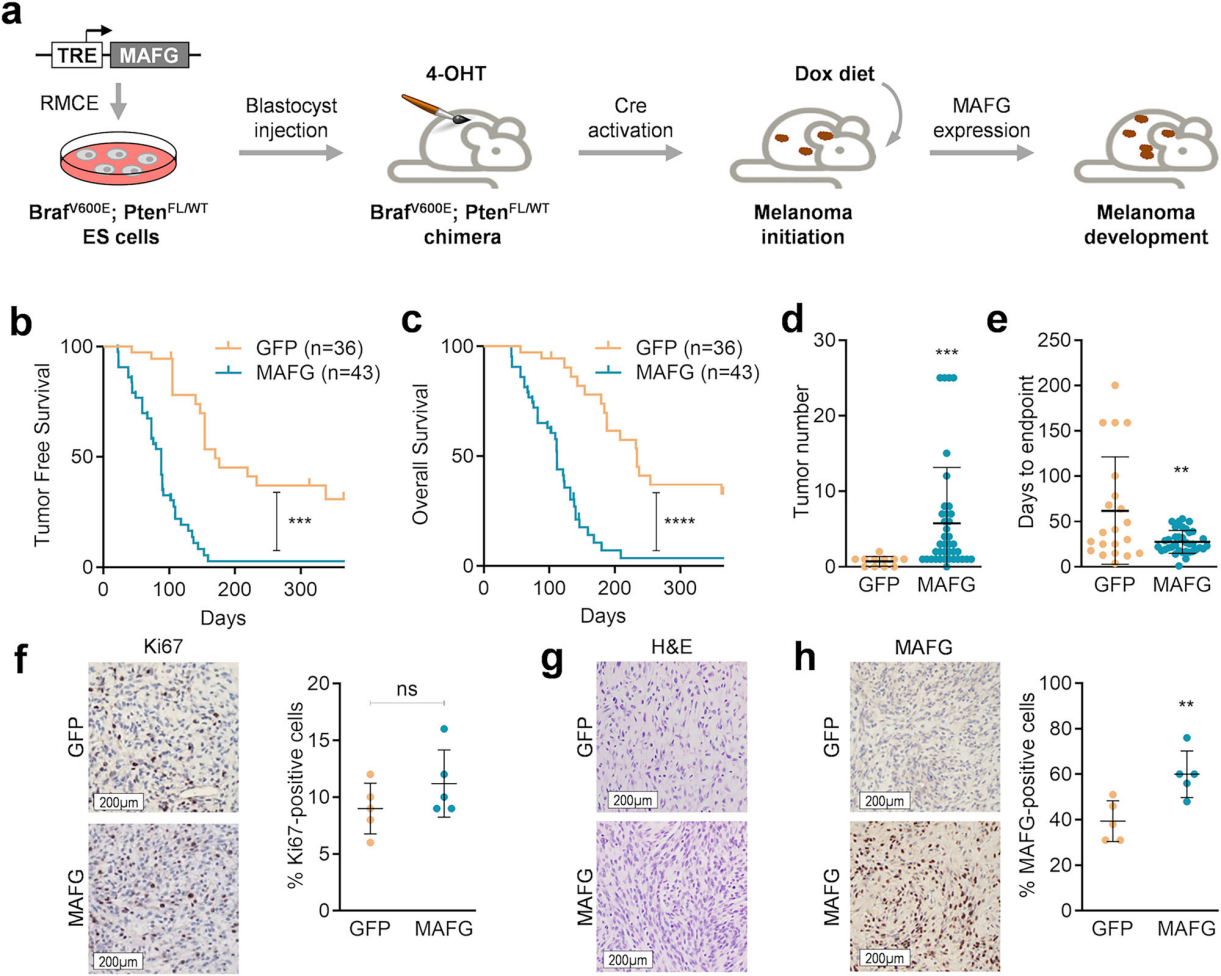
MAFG overexpression promotes melanoma in genetically engineered mouse models. **a** Outline of the embryonic stem cell-genetically engineered mouse model approach (Bok et al. 2019), where a Dox-inducible MAFG cDNA is expressed in Braf^V600E^; Pten^FL/WT^ melanocytes. (**b,c**) Kaplan-Meier curves comparing the tumor-free survival (i.e., tumor latency) (**b**) and overall survival (**c**) of BP^GFP^ (*n* = 36) or BP^MAFG^ (*n* = 43) experimental chimeras using the Gehan-Breslow-Wilcoxon test (*p* < 0.0001 for both comparisons). **d** Number of melanomas that developed in the experimental chimeras of BP^GFP^ (*n* = 11) or BP^MAFG^ (*n* = 39). Statistical significance was determined using Welch’s two-tailed *t* test (*p* = 0.0001). Error bars represent mean ± s.d. **e** Days from tumor emergence to euthanasia due to tumor burden in BP^GFP^ (*n* = 21) and BP^MAFG^ (*n* = 36) chimeras, using Welch’s two-tailed *t* test (*p* = 0.0156). Error bars represent mean ± s.d. **f** Ki67 immunohistochemistry on tumors from BP^MAFG^ and BP^GFP^ mice at endpoint and quantification of Ki67-positive cells per field. Statistical significance was determined using Welch’s two-tailed *t* test (*p* = 0.2231). Error bars represent mean ± s.d. **g** Representative H&E staining of tumors from BP^MAFG^ and BP^GFP^ mice. **h** MAFG immunohistochemistry (left) on tumors from BP^MAFG^ and BP^GFP^ mice at endpoint and quantification (right) of MAFG-positive cells per field. A total of *n* = 5 independent tumors from each group were analyzed. Statistical significance was determined using Welch’s two-tailed *t* test (*p* = 0.0097). Error bars represent mean ± s.d. Source data are provided as a Source Data file.

To investigate the role of MAFG in melanomagenesis in an immune-proficient setting, we used our embryonic stem cell-genetically engineered mouse modeling (ESC-GEMM) platform^15^. We generated chimeric mice on a C57BL/6 background harboring Cre-inducible *Braf*^V600E^ and heterozygous *Pten* deletion in melanocytes (BP model) with Dox-inducible MAFG (BP^MAFG^) or GFP (BP^GFP^) expression. Following induction of *Braf*^V600E^ and *Pten* loss, mice were placed on a Dox diet to activate MAFG or GFP expression (Fig. 2a). MAFG overexpression in BP^MAFG^ mice drastically reduced tumor latency (Fig. 2b) and reduced overall survival (Fig. 2c) compared to BP^GFP^ mice. In addition, BP^MAFG^ mice developed significantly more tumors (Fig. 2d), and these tumors on average grew moderately faster (Fig. 2e). Accordingly, at endpoint, there was a trend toward increased Ki67-positive proliferating cells (Fig. 2f). Tumors were relatively circumscribed masses composed predominantly of small, spindle-shaped cells with focal pigmentation. Tumors with MAFG overexpression had notably more nodular regions exhibiting increased cellularity (Fig. 2g and Supplementary Fig. 2a). No macro-metastases were detected in BP^MAFG^ or BP^GFP^ mice, indicating that MAFG promotes early progression but cannot induce metastasis in the BP model that lacks inherent metastatic propensity. We further confirmed that tumors from BP^MAFG^ mice expressed increased levels of MAFG (Fig. 2h).

To study whether the tumor suppressor background influences the effects of MAFG, we generated mice harboring *Braf*^V600E^ and homozygous deletion of *Cdkn2a* (BCC model) with Dox-inducible MAFG or GFP expression (BCC^MAFG^ and BCC^GFP^, respectively). Melanomagenesis was induced with 4-OHT, followed by the activation of ectopic MAFG and GFP expression with Dox. BCC^MAFG^ mice showed shorter tumor latency, decreased survival, and increased tumor numbers compared to BCC^GFP^ mice (Supplementary Fig. 2b–d), suggesting that MAFG may have oncogenic effects independent of the driver background. Accordingly, there is no association of *MAFG* expression levels or copy number gains with *BRAF*, *NRAS*, *NF1*, *CDKN2A*, or *PTEN* driver mutations in human melanoma (Supplementary Fig. 2e, f).

Oncogenic MAPK signaling results in ERK-mediated phosphorylation of MAFG, leading to increased MAFG protein stability^14^. This raises the possibility that MAFG is a critical downstream mediator of melanoma driver mutations that activate the MAPK pathway, and we therefore tested whether MAFG overexpression is sufficient to induce melanoma in the absence of mutant BRAF. To this end, we generated mice lacking an oncogenic driver but harboring homozygous deletion of *Pten* in melanocytes (PP model) with the Dox-inducible MAFG construct (PP^MAFG^). PP^MAFG^ mice were treated with 4-OHT and placed on a Dox diet or kept on a regular diet as a control. Neither PP^MAFG^ mice on a Dox diet nor PP^MAFG^ mice on a regular diet developed tumors (Supplementary Fig. 2g). These results demonstrate that MAFG overexpression promotes the progression of spontaneous melanoma but is insufficient for melanoma initiation.

### The oncogenic effects of MAFG are independent of NRF2

We next investigated the mechanism by which MAFG promotes melanoma progression. To this end, we performed RNA sequencing (RNAseq) analysis of WM164 and SKMel28 cells expressing either MAFG or GFP. MAFG induced significant transcriptional changes in WM164 and SKMel28 cells (925 and 598 differentially expressed genes, respectively; Supplementary Fig. 3a). MAFG is an obligate binding partner of the oncoprotein NRF2, prompting us to assess whether MAFG elicits its oncogenic effects via NRF2. MAFG-induced differentially expressed genes in WM164 cells did not significantly overlap with previously reported NRF2 expression signatures (Supplementary Fig. 3b). NRF2 silencing in WM164 cells overexpressing MAFG (Supplementary Fig. 3c) did not diminish proliferation or focus formation (Supplementary Fig. 3d, e). Moreover, MAFG overexpression did not increase the expression of a NRF2 transcriptional reporter (6xARE-Luciferase) (Supplementary Fig. 3f), indicating that the oncogenic effects of MAFG in melanoma are independent of NRF2.

### MAFG drives a phenotype switch

**Fig. 3:**
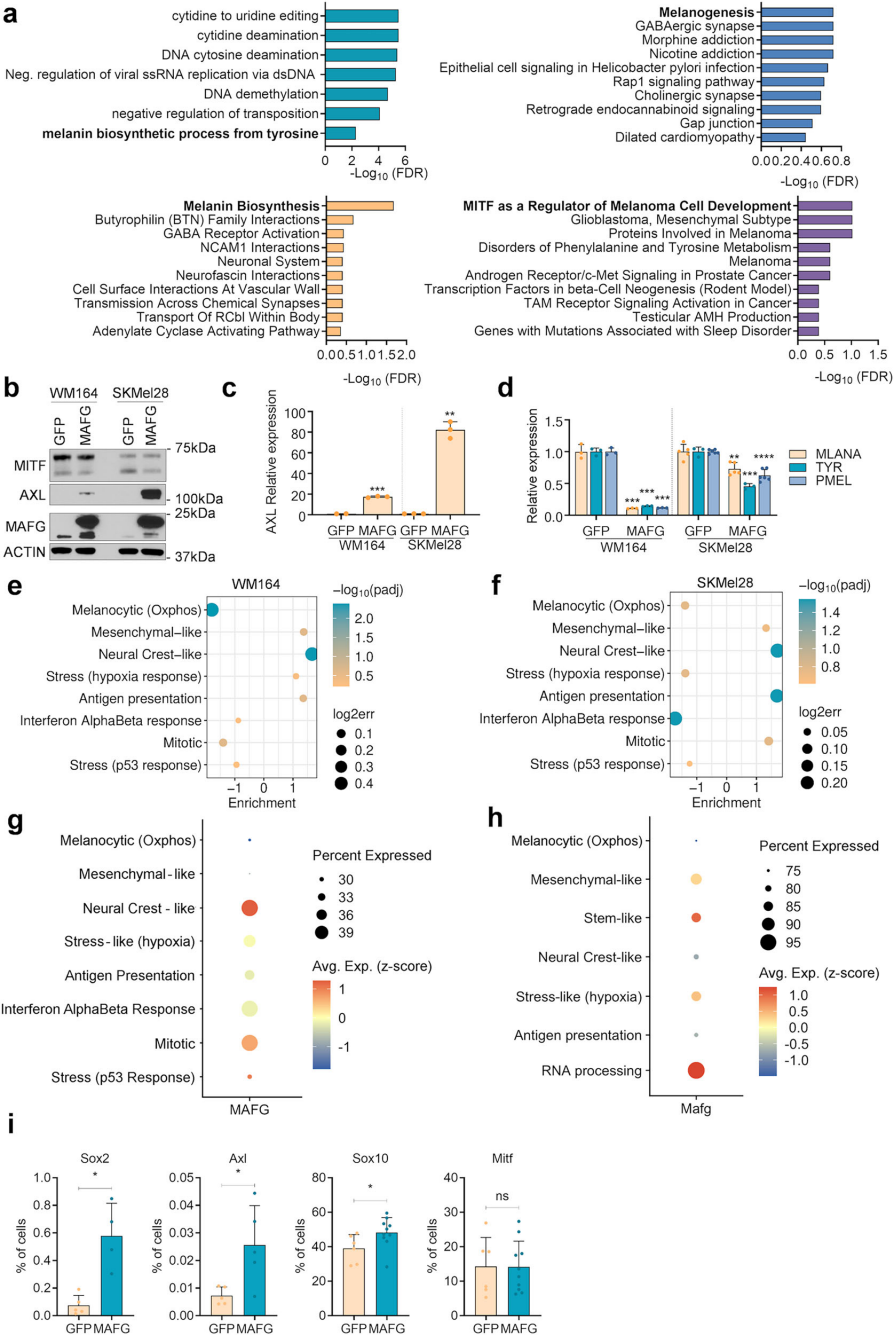
MAFG promotes a phenotype switch. **a** Pathway enrichment analysis of differentially expressed genes from RNA‑seq comparing WM164 cells overexpressing MAFG versus GFP. Pathway sources: GeneOntology (turquoise), Reactome (orange), Elsevier (blue), KEGG (purple). **b** Expression changes of MITF and AXL by western blot in WM164 and SKMel28 cells overexpressing either MAFG or GFP. The blot shown represents one out of three experiments with similar results. **c** Expression changes of *AXL* measured by qRT-PCR in WM164 (*p* = 0.0006) and SKMel28 (*p* = 0.0032) cells overexpressing either MAFG or GFP. Data represents a biological experiment out of two biological replicates measured in triplicate. Statistical significance was determined using Welch’s two-tailed *t* test. Error bars represent mean + s.d. **d** Expression changes of MITF target genes *MLANA*, *TYR* and *PMEL* measured by qRT-PCR in WM164 (*MLANA*, *p* = 0.0053; *TYR*, *p* = 0.0013; and *PMEL*, *p* = 0.0014), and SKMel28 (*MLANA*, p = 0.0128; *TYR*, *p* = 0.0011; and *PMEL*, *p* = 0.0001) cells overexpressing either MAFG or GFP. Statistical significance was determined using Welch’s two-tailed *t* test. Error bars represent mean + s.d. **e**, **f** Expression changes of the melanoma phenotype-associated gene signatures in WM164 (**e**) or SKMel28 (**f**) cells expressing MAFG versus GFP. **g** Expression of *MAFG* across human melanoma cell clusters defined by cell‑state gene signatures. The plot shows the distribution of *MAFG* expression within melanoma cell populations representing distinct phenotypic states from (Pozniak et al., 2024). **h** Expression of *Mafg* across murine melanoma cell clusters defined by cell‑state gene signatures. The plot depicts the distribution of *Mafg* expression within mouse melanoma cell populations representing distinct phenotypic states from (Karras et al., 2022). **i** Quantification of multiplexed immunohistochemistry of SOX2 (*p* = 0.0201) and AXL (*p* = 0.0440), and immunohistochemistry of SOX10 (*p* = 0.0156) and MITF (*p *= 0.9809) in tumors from BP^Mafg^ and BP^GFP^ mice. A total of *n* = 5 independent tumors from each group were analyzed. Statistical significance was determined for each protein staining by Unpaired two tailed *t* test. Error bars represent mean + s.d. ns, not significant; *p < 0.05; **p < 0.01; ***p < 0.001. Source data are provided as a Source Data file.

Pathway analyses of the MAFG-induced transcriptional changes revealed altered activity of several cancer-associated pathways (Supplementary Fig. 4a, b), most notably downregulation of melanin biosynthesis (Fig. 3a and Supplementary Fig. 4b). Melanin biosynthesis is chiefly regulated by the lineage transcription factor MITF^6,16^, which also plays a role in melanoma phenotype switching, where it defines the Melanocytic state, while AXL expression marks dedifferentiated states^5,17,18^. We therefore investigated whether MAFG affects MITF and promotes a phenotype switch. While MAFG overexpression moderately diminished *MITF* mRNA levels in WM164 but not SKMel28 cells (Supplementary Fig. 4c), it had minimal impact on MITF protein levels in both cell lines (Fig. 3b). Despite the lack of a robust effect on MITF levels, MAFG overexpression induced the expression of AXL (Fig. 3b, c) and repressed the MITF target genes *MLANA*, *TYR*, and *PMEL* (Fig. 3d). This observation demonstrates that MAFG regulates MITF activity rather than MITF expression levels and suggests a role for MAFG in melanoma phenotype switching. To test this, we analyzed previously established gene signatures for melanoma cell phenotypic states^19^ and observed that MAFG altered the expression of these signature genes. Specifically, the Melanocytic signature was diminished while the Neural Crest-like and Mesenchymal-like signatures were consistently enhanced in both cell lines (Fig. 3e, f). To further assess the association of MAFG expression with melanoma phenotypic states, we analyzed human^20^ and mouse^19^ scRNAseq data. This revealed that fewer cells in the Melanocytic cluster expressed MAFG, and expression levels were lower in this cluster compared to others (Fig. 3g, h and Supplementary Fig. 4d, e). To determine whether MAFG expression promotes a phenotype switch in the BP mouse model, we analyzed the expression of SOX2, AXL, SOX10, and MITF in tumors from BP^MAFG^ and BP^GFP^ mice by immunohistochemistry. SOX2 and AXL are highly expressed in the Stem cell-like, Mesenchymal, and Neural Crest-like cell states of murine melanoma but virtually absent in the Melanocytic state (Supplementary Fig. 4f). SOX10 is more broadly expressed but highest in the Stem-like cell state, while MITF is most abundantly expressed in cells of the Melanocytic state (Supplementary Fig. 4f). Consistent with a phenotype switch in vivo, the number of SOX2, AXL, and SOX10 positive cells is significantly increased in BP^MAFG^ tumors compared to BP^GFP^ tumors (Fig. 3i and Supplementary Fig. 4g). MITF levels were similar in BP^MAFG^ and BP^GFP^ tumors (Fig. 3i and Supplementary Fig. 4g), further corroborating our in vitro finding that MAFG influences MITF activity but not abundance. Thus, increased MAFG expression is inversely correlated with the Melanocytic state of melanoma cells.

Our results showed that MAFG promotes aggressive behaviors of MITF^hi^/AXL^lo^ human melanoma cells in the Melanocytic state. We next asked whether human melanoma cells in a more dedifferentiated state (MITF^lo^/AXL^hi^) are also sensitive to MAFG overexpression. Compared to WM164 and SKMel28 cells, A375 and WM793 cells express very low levels of MITF and its targets *PMEL* and *TYR,* while AXL is abundantly expressed (Supplementary Fig. 5a, b). Moreover, while WM164 and SKMel28 cells showed an enrichment of the Melanocytic state gene signature, the signatures defining the Mesenchymal-like and Neural Crest-like states were enriched in A375 and WM793 cells (Supplementary Fig. 5c). Notably, MAFG overexpression had no effect on proliferation and focus formation of A375 and WM793 cells (Supplementary Fig. 5d–f), suggesting context-dependent oncogenic properties of MAFG. Moreover, RNAseq revealed that MAFG overexpression provokes only modest expression changes in A375 cells (167 differentially expressed genes; Supplementary Fig. 5g), corroborating the results of the cell biological assays. These findings suggest that MAFG has oncogenic effects primarily in human melanoma cells that are in a Melanocytic cell state by inducing a phenotype switch.

### MAFG interacts with MITF

**Fig. 4:**
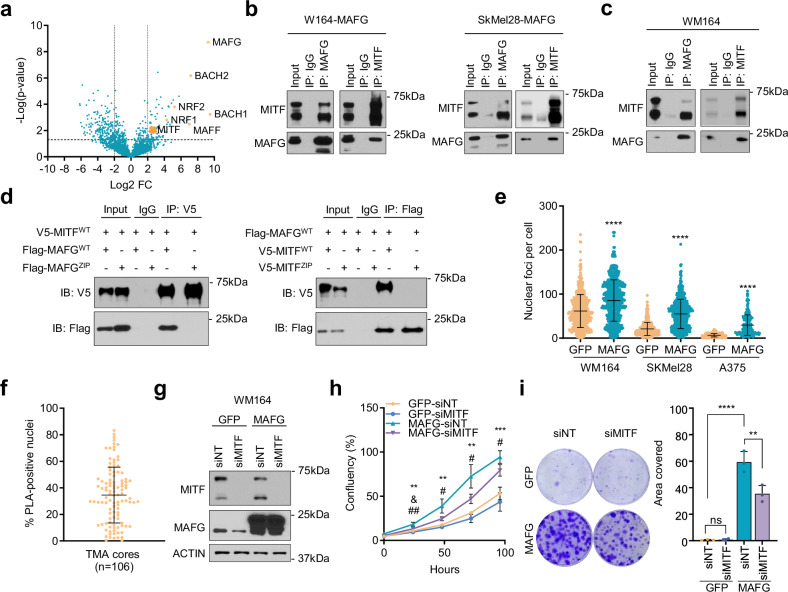
MAFG interacts with MITF. **a** Volcano plot of biotin-labeled proteins in WM164 cells expressing N-terminal MAFG-TurboID (*n* = 3 biological replicates) versus TurboID control (*n* = 3 biological replicates). Canonical MAFG interaction partners, as well as MITF, are highlighted. **b** Co-immunoprecipitation of MAFG and MITF in WM164 and SKMel28 cells overexpressing MAFG. Blot is representative of three experiments with similar results. **c** Co-immunoprecipitation of endogenous MAFG and MITF in parental WM164 cells. Blot is representative of three experiments with similar results. **d** Co-immunoprecipitation of flag-MAFG with either wildtype V5-MITF or mutant V5-MITF^Zip^ and of V5-MITF with either wildtype flag-MAFG or mutant flag-MAFG^Zip^. Blot is representative of three experiments with similar results. **e** Quantification of the proximity ligation assays (PLA) from Supplementary Fig. 9a in WM164, SKMel28 and A375 cells overexpressing either MAFG or GFP. Each individual dot represents a PLA-positive nucleus. Statistical significance was determined using Welch’s two-tailed *t* test. Stats represent the comparison between MAFG and GFP in each cell line, WM164 (*p* < 0.0001), SkMel28 (*p* < 0.0001) and A375 (*p* < 0.0001). Error bars represent mean ± s.d. **f** Quantification of PLA-positive nuclei on a melanoma metastasis TMA. Error bars represent mean ± s.d. **g** Western blot validating the silencing of MITF in WM164 cells overexpressing MAFG or GFP. The blot is representative of three experiments with similar results. **h**, **i** Proliferation (**h**) and focus formation (**i**) assays in WM164 cells overexpressing MAFG or GFP, transfected with siMITF or control siRNA. For the proliferation assay, *n* = 4 technical replicates are represented. Statistical significance was determined using Welch’s two-tailed *t* test for each time point. *p*-value of each comparison at 96 h is as follows: GFP-siNT vs MAFG-siNT (*p* = 0.0002); GFP-siNT vs GFP-siMITF (*p *= 0.1892); MAFG-siNT vs MAFG-siMITF (*p* = 0.0197); GFP-siMITF vs MAFG-siMITF (*p* = 0.0021). Error bars represent mean ± s.d. For the focus formation assay, *n* = 3 technical replicates are represented. Statistical significance was determined by one‑way ANOVA with Tukey’s multiple‑comparison test. GFP-siNT vs MAFG-siNT (*p* < 0.0001); GFP-siNT vs GFP-siMITF (*p* > 0.9999); MAFG siNT vs MAFG siMITF (*p* = 0.0019). Error bars represent mean + s.d. ns, not significant; * *p* < 0.05; **p < 0.01; ***p < 0.001; ****p < 0.0001; # *p* < 0.05; ## *p* < 0.01; & *p* < 0.05. Source data are provided as a Source Data file.

The critical role of MITF in phenotype switching^3^ and the finding that MAFG overexpression has oncogenic effects only in melanoma cells expressing MITF suggested that MAFG deregulates MITF activity. However, the modest MITF expression changes induced by MAFG overexpression indicated that MAFG may impact MITF activity through non-transcriptional mechanisms. Interestingly, MITF is a basic helix-loop-helix leucine zipper transcription factor, and we hypothesized that MAFG directly interacts with MITF via their leucine zippers. To explore this possibility, we first performed biotin proximity labeling using an N-terminal TurboID-MAFG fusion construct in WM164 cells (Supplementary Fig. 6a, b). This approach detected known MAFG dimerization partners such as MAFF, BACH1, BACH2, NRF1, and NRF2 (Fig. 4a and Supplementary Dataset 1), validating the ability of this approach to identify proteins that interact with MAFG. Notably, biotin proximity labeling also identified MITF as a putative MAFG interaction partner (Fig. 4a and Supplementary Dataset 1). We validated the interaction by reciprocal co-immunoprecipitation of MAFG and MITF in WM164 and SKMel28 cells overexpressing MAFG (Fig. 4b). Similarly, we observed the interaction of MAFG with MITF by co-immunoprecipitation in parental WM164 cells expressing endogenous MAFG (Fig. 4c). To test whether MAFG and MITF interact through their leucine zippers, we mutated residues that, based on AlphaFold3 predictions (Supplementary Fig. 6c), interlock the MAFG and MITF zipper domains (Met100, Leu104, Leu107 in MAFG and Leu281, Ile285 in MITF). We expressed the MAFG^Zip^ and MITF^Zip^ mutants in HEK293 cells and performed co-immunoprecipitation assays. This revealed that MAFG interacts with wildtype MITF but not MITF^Zip^, while MITF interacts with wildtype MAFG but not MAFG^Zip^ (Fig. 4d), indicating that the interaction between MAFG and MITF involves their respective leucine zippers. MAFG is phosphorylated by ERK at Ser124, and we tested whether phospho-Ser124 promotes the interaction with MITF. Phospho-mimetic (S124D) and phospho-dead (S124A) mutant MAFG readily co-immunoprecipitated MITF in WM164 cells (Supplementary Fig. 6d), indicating that Ser124 phosphorylation is not required for mediating the interaction with MITF.

To test whether MAFG affects MITF subcellular localization, we performed immunofluorescence (IF) in WM164, SKMel28, and A375 cells expressing MAFG or GFP. IF validated overexpression and nuclear localization of MAFG (Supplementary Fig. 7) and demonstrated that MITF levels and localization were not affected by MAFG in WM164 and SKMel28 cells, while MITF was not detectable in A375 cells (Supplementary Fig. 8). We then performed proximity ligation assays (PLA) in WM164, SKMel28 and A375 cells overexpressing MAFG or GFP. Nuclear PLA foci were present in WM164 and SKMel28 cells and, to a lesser extent, in A375 cells (Fig. 4e and Supplementary Fig. 9a). Notably, the number of foci increased significantly upon MAFG overexpression in all three cell lines (Fig. 4e and Supplementary Fig. 9a). We also performed PLA on a melanoma metastasis TMA. This revealed the presence of nuclear PLA foci in human melanoma specimens (Fig. 4f and Supplementary Fig. 9b) and showed a correlation between the number of PLA-positive nuclei and the number of PLA foci per nucleus (Supplementary Fig. 9c). These results reveal a direct interaction of MAFG with MITF in melanoma cell lines and patient specimens, highlighting the clinical relevance of this interaction.

Given the interaction of MAFG with MITF, we determined whether MITF is required for the oncogenic effects of MAFG. To this end, we silenced MITF in WM164 and SKMel28 using a SMART pool of 4 siRNAs (Fig. 4g and Supplementary Fig. 10a). MITF expression was required for proliferation and focus formation of SKMel28 control cells expressing GFP (Supplementary Fig. 10b, c). While this demonstrated the critical role of MITF in SKMel28 cells, it precluded conclusions as to whether MITF is required for the effects of MAFG. However, WM164 control cells tolerated the silencing of MITF, and we observed a significant reduction of MAFG-induced proliferation and focus formation upon MITF silencing (Fig. 4h, i). We used Dox-inducible shRNAs as an orthogonal approach of MITF silencing in WM164 cells overexpressing MAFG (Supplementary Fig. 10d). This MITF silencing approach also decreased proliferation and focus formation (Supplementary Fig. 10e, f). These results suggest that MITF is required for the oncogenic effects of MAFG in melanoma.

### MAFG overexpression redistributes MITF genome binding

**Fig. 5:**
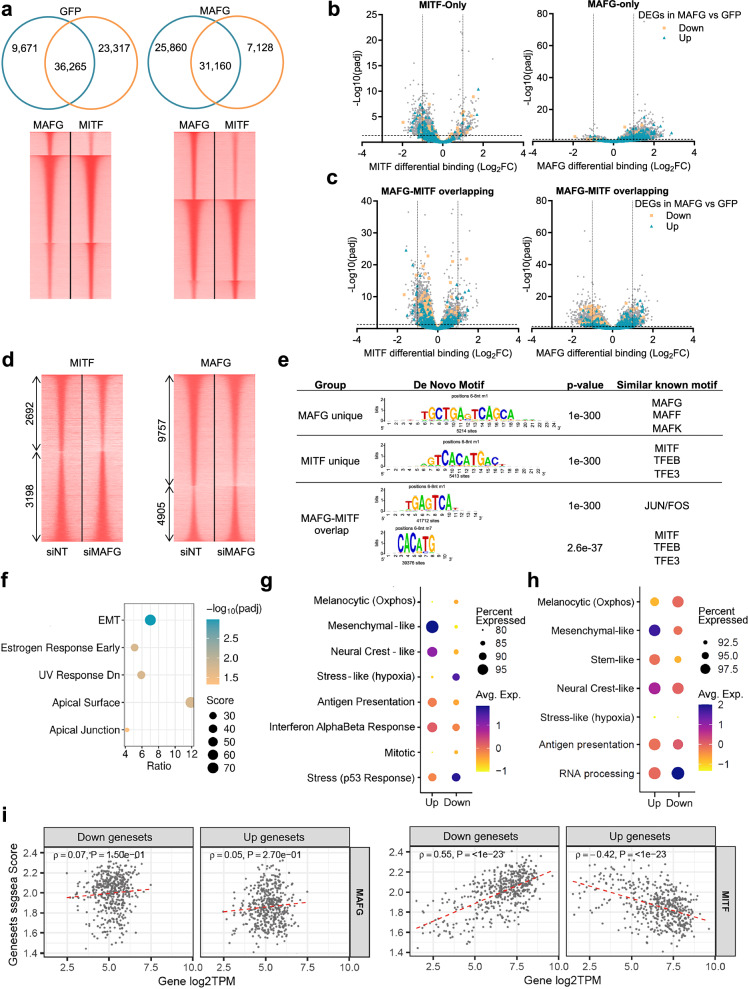
MAFG impacts MITF target gene binding and transactivation. **a** Venn diagrams (top) and density plots (bottom) showing the overlap between MAFG and MITF peaks identified by CUT&RUN in WM164 cells expressing GFP (left) or MAFG (right). **b** Volcano plots showing differential CUT&RUN peak heights for MITF (left) and MAFG (right) at non-overlapping genomic sites. **c** Volcano plots showing differential CUT&RUN peak heights for MITF (left) and MAFG (right) at overlapping genomic sites. For both (**b**, **c**), orange squares and turquoise triangles are downregulated and upregulated genes, respectively, when MAFG is overexpressed (padj < 0.05; Fold Change > 2). **d** Density maps showing significantly altered MAFG and MITF peaks upon MAFG silencing. **e** Motif analysis for MAFG-unique, MITF-unique and MAFG-MITF overlapping peaks. **f** Gene Ontology analysis using the Hallmark dataset GSEA for genes that are significantly differentially expressed and that harbor an MITF peak that is significantly altered upon MAFG overexpression. **g**, **h** Dot blots showing the association of the MAFG-regulated MITF target gene sets with the human (**g**) and murine (**h**) melanoma cell states. **i** Expression correlation of MAFG and MITF with the MAFG-regulated MITF target gene sets in the TCGA skin cutaneous melanoma dataset.

To understand how MAFG modulates MITF activity, we first mapped their genome-wide binding patterns. To this end, we performed CUT&RUN in WM164 cells overexpressing MAFG or GFP. In control cells, we identified 45,936 MAFG peaks and 59,582 MITF peaks, with substantial co-occupancy (36,265 overlapping peaks; Fig. 5a and Supplementary Dataset 2). Analysis of canonical MITF target genes (*TYR*, *TYRP1*, *DCT*, *MLANA*, and *PMEL*/*CDK2*) confirmed MAFG binding at MITF-occupied regulatory regions, though MITF exhibited more prominent binding (Supplementary Fig. 11a).

MAFG overexpression induced substantial redistribution of both MAFG and MITF genome occupancy. While total MAFG peaks increased modestly (~24%), MITF peaks decreased by ~36% (Fig. 5a). This redistribution primarily affected non-overlapping MAFG and MITF peaks, with unique MAFG peaks increasing by 167% while unique MITF peaks decreased by 69%. In contrast, sites with concomitant MAFG~MITF binding decreased by only 14% (Fig. 5a), suggesting these co-bound regions are more stable. Analysis of high-confidence differential peaks (present in at least 2 of 3 replicates; −1> log2FC > 1; p_adj_ < 0.05) confirmed that MAFG overexpression enhanced MAFG binding and reduced MITF binding predominantly at their respective unique sites (Fig. 5b, c and Supplementary Dataset 3). Notably, MAFG overexpression attenuated MITF binding at its canonical target genes *TYR*, *TYRP1*, *DCT*, *MLANA*, and *PMEL*/*CDK2* (Supplementary Fig. 11b) while increasing MAFG binding at genes with unique MAFG sites, including *GLIS3*, *IL7R*, and *AXL* (Supplementary Fig. 11c, d). The observation of unique MAFG binding at the *AXL* locus suggests that MAFG can directly regulate *AXL* expression independent of MITF.

To assess whether MITF occupancy is also sensitive to MAFG reduction, we silenced MAFG in WM164 cells and performed CUT&RUN. While MAFG peaks were predominantly reduced as expected, MITF binding was only modestly affected, with slightly more peaks increased (54%) than decreased (Fig. 5d). These findings indicate that basal MAFG levels have limited influence on MITF genome occupancy, whereas MAFG overexpression substantially reduces MITF binding affinity, both at sites where MITF binds with and without MAFG.

We next determined the genome binding preferences of the MAFG~MITF heterodimer. Motif enrichment analysis demonstrated that peaks unique to MAFG and MITF overlapped with the consensus MARE and E/M-box motifs, respectively (Fig. 5e). Interestingly, genomic sites where both MAFG and MITF bind (presumably as heterodimer) are most strongly enriched for the AP-1 motif, which is naturally embedded in the MARE motif, and the E-box motif is also weakly enriched (Fig. 5e). Thus, while canonical dimers containing MAFG and MITF strongly associate with their respective binding motifs, the heterodimer binds to weaker motifs that partially resemble MARE (AP-1) and E-box motifs.

### MAFG-MITF interaction drives phenotype-associated gene expression

To determine the transcriptional consequences of MAFG-mediated changes in MITF genome binding, we integrated the CUT&RUN data with RNAseq profiles from WM164 cells overexpressing MAFG. We hypothesized that altered MITF occupancy would correlate with changes in target gene expression. Unexpectedly, genes harboring MITF peaks, either unique or overlapping with MAFG, showed variable expression changes in response to MAFG overexpression, with some genes induced and others repressed regardless of whether MITF occupancy increased or decreased (Fig. 5b, c and Supplementary Dataset 3). In addition, many genes with modest changes in MITF occupancy exhibited significant expression alterations (Fig. 5b, c and Supplementary Dataset 3). This complexity suggests that MAFG impacts both MITF genome occupancy and transcriptional activity and that additional factors beyond MITF contribute to determining the transcriptional output.

To identify genes directly responsive to the MAFG-MITF interaction, we prioritized those (i) harboring an MITF peak that significantly changed with MAFG overexpression and (ii) showing significant differential expression. This analysis identified 106 upregulated and 49 downregulated genes (Supplementary Dataset 4). Gene Ontology analysis revealed significant enrichment of the Epithelial Mesenchymal Transition (EMT) pathway (Fig. 5f), linking MAFG-dysregulated MITF target genes to phenotype switching.

To validate this association, we examined expression patterns of these gene sets across melanoma cell states. In both human and murine single-cell datasets, MAFG-upregulated MITF targets showed high expression in Neural Crest-like and Mesenchymal states, while MAFG-downregulated targets were reduced in these states (Fig. 5g, h). This pattern mirrors the expression of MAFG itself across cell states. Analysis of the TCGA skin cutaneous melanoma dataset further showed that while MAFG expression did not correlate with either gene set, MITF levels showed strongly positive and negative correlations with the downregulated and upregulated gene sets, respectively (Fig. 5i). This observation suggests that MITF primarily controls the expression of these genes, while MAFG alters both the activating and repressive outputs of MITF, effectively reprogramming its transcriptional activity. Collectively, these findings demonstrate that MAFG dysregulates an MITF-dependent transcriptional program associated with dedifferentiated melanoma cell states, mechanistically linking the MAFG-MITF interaction to phenotype switching.

### The MAFG~MITF dimer has oncogenic effects

**Fig. 6:**
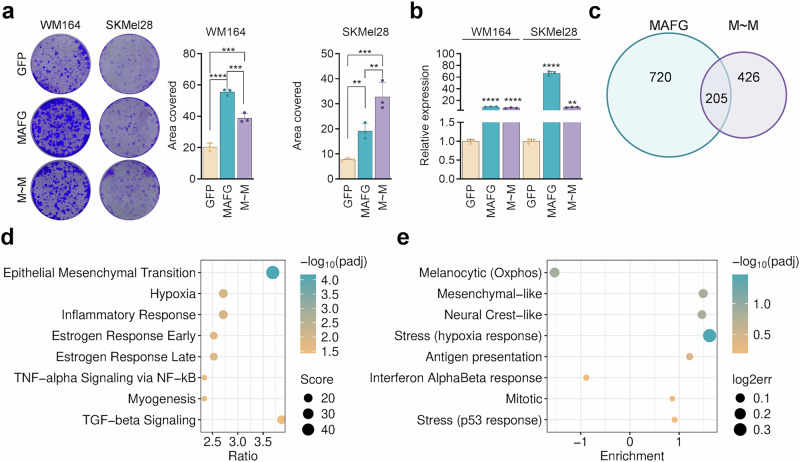
The MAFG~MITF dimer has oncogenic effects. **a** Focus formation assays (left) and quantification (right) in WM164 and SKMel28 cells overexpressing a MAFG~MITF dimer or a GFP control. Data represent *n* = 3 technical replicates from one out of two biological replicates. Statistical significance was determined using two‑sided one‑way ANOVA followed by Tukey’s multiple‑comparisons test. For WM164: GFP vs MAFG (*p* < 0.0001), GFP vs M~M (*p* = 0.0003), MAFG vs M~M (*p* = 0.0005). For SKMel28: GFP vs MAFG (*p* = 0.0246), GFP vs M~M (*p* = 0.0005), MAFG vs M~M (*p* = 0.0108). **b** qRT-PCR showing expression of *AXL* in WM164 and SKMel28 cells overexpressing the tethered MAFG~MITF (M~M) dimer. Statistical significance was evaluated by two‑sided one‑way ANOVA with Tukey’s multiple comparisons. For WM164: GFP vs MAFG (*p* < 0.0001), GFP vs M~M (*p* < 0.0001). For SKMel28: GFP vs MAFG (*p* < 0.0001), GFP vs M~M (*p* = 0.0056). Error bars represent mean + s.d. **c** Venn diagram showing the overlap of differentially expressed genes upon overexpression of MAFG or the tethered MAFG~MITF dimer in WM164 cells. **d** Pathway analysis of the overlapping genes in (**c**). **e** Expression changes of the melanoma phenotype-associated gene signatures in WM164 cells expressing the tethered MAFG~MITF dimer or GFP. **p < 0.01; ***p < 0.001; ****p < 0.0001. M~M, MAFG~MITF dimer fused via a flexible linker. Source data are provided as a Source Data file.

To directly test whether the MAFG-MITF interaction drives the observed oncogenic effects, we generated a tethered dimer construct in which MAFG and MITF proteins are covalently fused via a flexible linker [RS(GGGS)_4_GGRS]. This approach, previously used to study the NRF2~MAFG dimer^21^, ensures constitutive heterodimerization and eliminates the influence of competing binding partners. Expression of the MAFG~MITF dimer in WM164 and SKMel28 cells (Supplementary Fig. 12a) recapitulated key phenotypes observed with MAFG overexpression. The dimer enhanced focus formation (Fig. 6a) and induced *AXL* expression (Fig. 6b), though effects on proliferation were modest (Supplementary Fig. 12b). RNA sequencing of WM164 cells expressing the MAFG~MITF dimer revealed 631 differentially expressed genes, with substantial overlap (205 genes) with the MAFG overexpression signature (Fig. 6c). Gene set enrichment analysis identified Epithelial Mesenchymal Transition as the top enriched pathway (Fig. 6d), consistent with our previous analysis of MAFG-dysregulated MITF target genes (Fig. 5e). Importantly, the dimer induced a phenotype switch, reducing the Melanocytic state signature while increasing the Mesenchymal-like, Stress-like, and Neural Crest-like signatures (Fig. 6e). These findings demonstrate that forced MAFG~MITF heterodimerization recapitulates the effects of MAFG overexpression, implicating the physical interaction between MAFG and MITF as a key mechanism underlying the oncogenic potential of MAFG.

### sMAF family members MAFF and MAFK interact with MITF

Given the functional redundancy observed among sMAF family members^22^, we investigated whether MAFF and MAFK, which share 68% and 78% sequence homology with MAFG^12^, respectively, also contribute to melanomagenesis. Analysis of the TCGA dataset revealed that while *MAFG*, *MAFF*, and *MAFK* amplifications are mutually exclusive, copy number gains frequently co-occur in 33–50% of melanoma samples (Supplementary Fig. 13a–d). Notably, amplification of any single sMAF gene correlated with significantly worse patient survival (Supplementary Fig. 13e), suggesting oncogenic potential for all three family members. To compare the oncogenic potential of sMAFs family members, we overexpressed MAFF and MAFK in WM164 and SKMel28 cells. Similar to MAFG, both MAFF and MAFK increased AXL expression without affecting MITF levels (Supplementary Fig. 13f) and promoted proliferation as potently as MAFG (Supplementary Fig. 13g). However, MAFF and MAFK enhanced focus formation less effectively than MAFG (Supplementary Fig. 13h, i), suggesting quantitative differences in oncogenic potential. Co-immunoprecipitation experiments demonstrated that all three sMAF proteins bind MITF (Supplementary Fig. 13j); however, MAFG exhibited stronger binding than MAFF and MAFK based on normalized co-IP efficiency (Supplementary Fig. 13k). These data indicate that MAFF and MAFK possess oncogenic activity in melanoma, likely through MITF interaction, but MAFG is the most potent family member. The co-occurrence of sMAF copy number gains in patient tumors suggests that cumulative sMAF activity may drive melanoma progression.

### MAFG is a vulnerability in melanoma

**Fig. 7:**
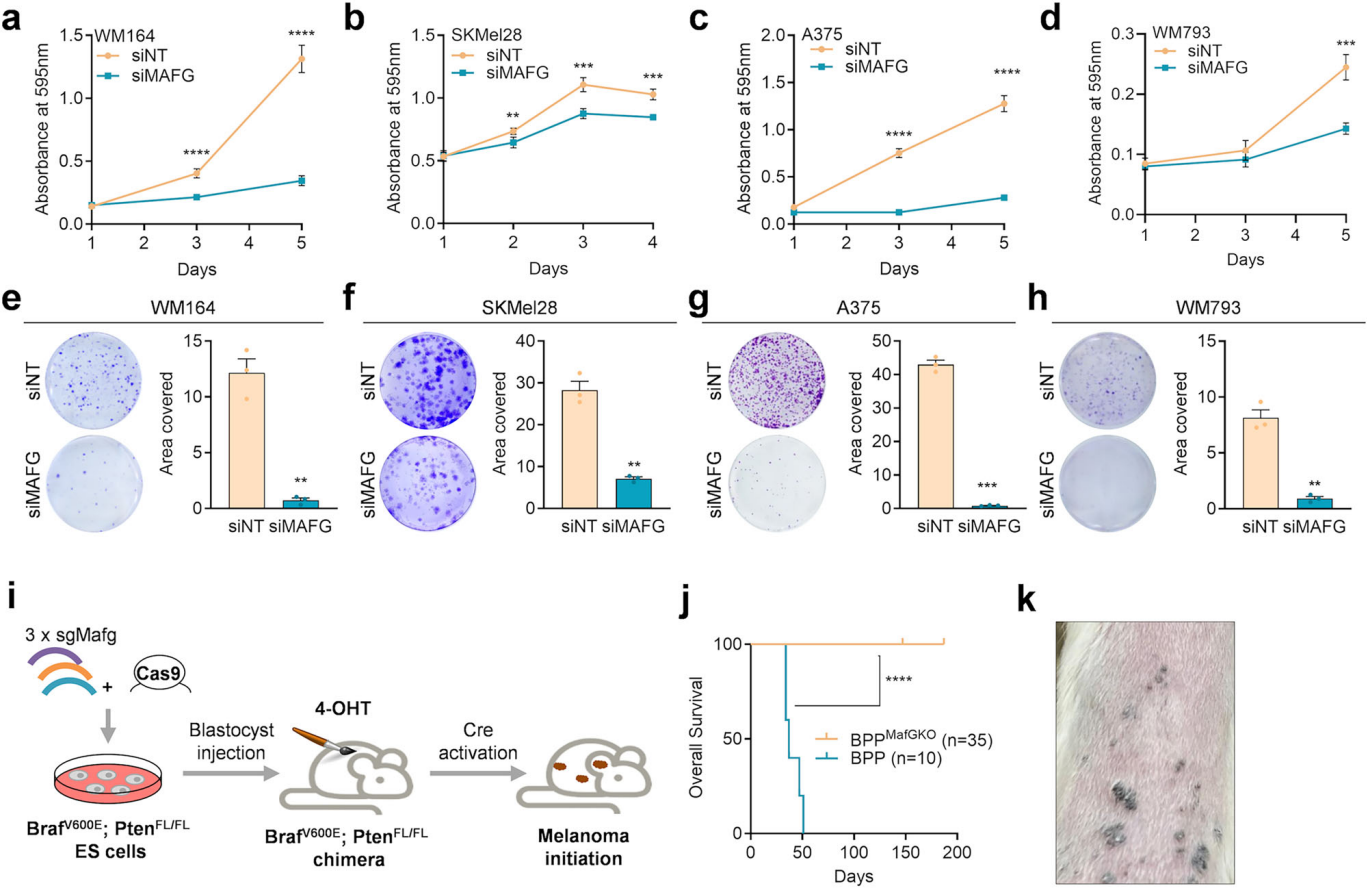
MAFG is a vulnerability in melanoma. **a**–**d** Proliferation assays of WM164 (**a**, *n* = 5 technical replicates per group), SKMel28 (**b**, *n* = 5 technical replicates per group), A375 (**c**, *n* = 6 technical replicates per group) and WM793 (**d**, *n* = 6 technical replicates per group) upon silencing of MAFG. Statistical significance was determined using Welch’s two-tailed *t* test for each time point. *p*-values of each cell line at endpoint are as follows: WM164 (*p* < 0.00001); SKMel28 (*p* = 0.0002); A375 (*p* < 0.00001); WM793 (*p *= 0.0001). **e**–**h** Focus formation assays of WM164 (*p* = 0.0105) (**e**), SKMel28 (*p* = 0.0076) (**f**), A375 (*p* = 0.009) (**g**) and WM793 (*p* = 0.0069) (**h**) cells upon silencing of MAFG. Statistical significance was calculated by comparing siMAFG vs siNT using Welch’s two-tailed *t* test. **i** Outline of the embryonic stem cell-genetically engineered mouse model approach (Bok et al. 2019) where Braf^V600E^; Pten^FL/FL^ embryonic stem cells are electroporated with 3 guide RNAs against the murine *Mafg* gene together with the Cas9 protein. **j** Kaplan-Meier curve comparing the overall survival of BPP (*n* = 10) or BPP^MafgKO^ (*n* = 35) experimental chimeras using the Gehan-Breslow-Wilcoxon test. **k** Nevi on a 25-week-old BPP^MafgKO^ mouse. *** *p* < 0.001; **** *p* < 0.0001. Source data are provided as a Source Data file.

Given the potent oncogenic effects of MAFG in melanoma, we next tested whether basal expression of MAFG is required to sustain the malignant state of melanoma cells. To this end, we silenced MAFG in WM164, SKMel28, A375, and WM793 cells (Supplementary Fig. 14a) and performed proliferation and focus formation assays. MAFG silencing robustly diminished proliferation of all four melanoma cell lines (Fig. 7a–d). Similarly, focus formation was markedly reduced upon MAFG silencing (Fig. 7e–h), indicating that MAFG is required for the growth of human melanoma cells in vitro. These results prompted us to determine the contribution of basal MAFG expression to melanomagenesis. To this end, we transiently transfected Braf^V600E^; Pten^FL/FL^; Tyr-CreERt2; CAGs-LSL-rtTA3; CHC (BPP) ES cells on a C57BL/6 background^15,23^ with Cas9 protein and a combination of three sgRNAs targeting the two coding exons to knockout *Mafg* (Supplementary Fig. 14b). Several clones showed homozygous deletions in the *Mafg* gene (Supplementary Fig. 14c) and there was no detectable MAFG protein expression in these clones (Supplementary Fig. 14d). Using BPP^MafgKO^ ES cell clones and control BPP clones with intact MAFG expression, we generated chimeric mice and induced melanomagenesis via topical administration of 4-OHT on the back skin (Fig. 7i). BPP control mice readily developed melanoma, requiring euthanasia due to the size of the primary tumors after 6-7 weeks similar to our previous BPP cohorts^15,23^. In contrast, BPP^MafgKO^ mice failed to form bona fide tumors and survived up to ~ 25 weeks post 4-OHT administration when we ended the experiment (Fig. 7j). Interestingly, BPP^MafgKO^ mice developed benign nevi despite the absence of MAFG expression (Fig. 7k), indicating that MAFG may be important for melanoma formation by promoting the malignant transition from nevi to frank melanoma. We then determined whether the role of MAFG in melanoma formation extends to the other sMAF family members. To this end, we silenced MAFG, MAFK, and MAFF in WM164 and A375 cells (Supplementary Fig. 15a) and assessed proliferation and focus formation. Notably, while MAFG knockdown significantly reduced melanoma cell growth, the silencing of MAFK or MAFF had no significant effect on proliferation (Supplementary Fig. 15b, c) or focus formation (Supplementary Fig. 15d, e). Thus, MAFG plays a critical role in melanoma that is not shared by the sMAF family members MAFK and MAFF.

## Discussion

Non-mutational mechanisms are emerging as potent drivers of melanomagenesis. Among these, transcription factor dysregulation enables dynamic and reversible transcriptional adaptation that governs melanoma progression and metastasis. Here, we demonstrate that overexpression of the sMAF transcription factor MAFG potently promotes melanomagenesis at least in part by interacting with MITF and promoting a phenotype switch to a dedifferentiated cell state. Moreover, our study identifies MAFG as a melanoma cell-specific vulnerability.

Expression and silencing studies have implicated MAFG in several cancer types^11,24,25^ and in the development of platinum resistance in lung and ovarian cancer^26,27^. However, direct investigations into the oncogenic effects of elevated MAFG expression, particularly using in vivo models, remain limited. Our in vitro and in vivo studies demonstrate that MAFG functions as a bona fide pro-tumorigenic driver in melanoma. Knockout studies have revealed functional redundancy among the sMAF family members^12^, and we discovered that MAFF and MAFK also exhibit oncogenic potential. The co-occurrence of low-level copy number gains of sMAF genes, alongside mutually exclusive amplifications, may suggest a threshold level of sMAF proteins necessary for optimal oncogenic activity. However, MAFF and MAFK appear to be less effective in promoting melanoma cell growth. This could be due to less robust interactions of MAFF and MAFK with MITF, but also raises the possibility that MAFG possesses distinct functions that synergize with its effect on MITF to facilitate melanoma progression. Moreover, MAFG contains a serine residue absent in MAFF and MAFK, which is phosphorylated by ERK to increase protein stability^14^. The prevalent hyperactivation of the MAPK pathway in melanoma may specifically increase MAFG protein levels, thereby promoting melanomagenesis. This notion is further supported by our observation that MAFG overexpression requires oncogenic BRAF to enhance the development of melanoma.

The observation that MAFG exerts its pro-tumorigenic effects only in the presence of an initiating mutation underscores its role in melanoma progression. Phenotype switching is emerging as a key facilitator of this process^28^, with MITF and AXL playing a central role in governing melanoma cell states^3^. Our findings indicate that MAFG induces a dedifferentiated cell state through its interaction with MITF and modulating its transcriptional activity. This attenuation in MITF output correlates with increased AXL expression, consistent with the well-established inverse relationship between MITF and AXL levels in melanoma cells^29–31^. In addition, our data showed that MAFG may directly regulate AXL expression by binding to its regulatory region. This supports a model in which MAFG contributes to phenotype switching by shifting cells toward an MITF^low^/AXL^high^ state, a hallmark of dedifferentiation, thus contributing to the growing number of molecular mechanisms involved in this process.

Our genomic and transcriptomic analyses revealed that MAFG employs multiple mechanisms to modulate MITF-dependent transcription. First, MAFG overexpression substantially redistributes MITF genome occupancy, reducing MITF binding at E/M-boxes of canonical target genes while the MAFG~MITF complex occupies alternative genomic sites harboring AP-1 motifs and weak E-boxes. This redistribution alone would be expected to alter the MITF transcriptional program. However, we also observed transcriptional changes at genes where MITF binding was not significantly altered, suggesting that MAFG additionally modulates the transcriptional activity of MITF independent of binding site redistribution. This could occur through several non-mutually exclusive mechanisms by altering the recruitment of transcriptional co-activators or co-repressors, modifying the interaction with the basal transcription machinery, or influencing local chromatin accessibility. The complexity of these effects, where some MITF target genes are upregulated while others are downregulated regardless of binding changes, indicates that the MAFG~MITF complex represents a qualitatively different transcriptional entity than MITF homodimers. This multifaceted regulation allows MAFG to fine-tune the MITF transcriptional program to promote a dedifferentiated cell state.

Given the central role of MITF in melanoma phenotype switching, melanocyte differentiation, and pigmentation, it is tightly regulated through multiple mechanisms, including transcriptional control, posttranslational modifications, and interactions with co-factors^6^. MITF typically dimerizes with members of the MiT/TFE family (MITF, TFEB, TFE3, and TFEC), but can also form atypical heterodimers with the transcription factors β-Catenin and LEF-1^32,33^, suggesting that MITF may influence the output of the WNT pathway, which has been implicated in melanoma phenotype switching^34^. While the contribution of the direct interaction of MITF with β-Catenin or LEF-1 to melanomagenesis is not yet known, these observations, combined with our findings, suggest a regulatory mechanism wherein dimerization partner choice fundamentally alters the DNA binding pattern and transcriptional activity of MITF to promote melanomagenesis. Moreover, MAFG may also interact with other members of the MiT/TFE family to influence their transcriptional output and affect several cellular processes such as autophagy and energy metabolism.

Beyond MITF, sMAF proteins interact with CNC/BACH family transcription factors, most notably NRF2^12^. While NRF2 has been implicated in melanomagenesis^35,36^, its role remains debated^37^, and our data demonstrate that NRF2 is dispensable for the oncogenic effects of MAFG. In addition, MAFG recruits an epigenetic silencing complex comprising BACH1, CHD8, and DNMT3B in both colon cancer and melanoma^14,38^. Given that MAFG interacts with BACH1 and MITF via its leucine zipper domain, these complexes are likely mutually exclusive and form and function independently. Our tethered MAFG~MITF dimer experiments demonstrate that the MAFG-MITF interaction alone is sufficient to drive oncogenic phenotypes. However, MAFG may exert additional effects through the BACH1-containing corepressor complex or other dimerization partners, potentially explaining why MAFG is essential for melanoma maintenance even in dedifferentiated cells where MITF levels are low.

Our findings demonstrated that basal MAFG expression is critical for melanoma development. Previous studies showed that germline MAFG deficiency in mice allowed for normal development and provoked only mild phenotypes, including thrombocytopenia and juvenile ataxia that tends to diminish with age^22^. Moreover, MAFG^-/-^ mice exhibit normal pigmentation, indicating that MAFG is not required for melanocyte formation and function. This aligns with our observation that BPP^MafgKO^ mice, despite exhibiting impaired melanoma formation, show normal nevus development. These findings underscore the important role of MAFG in the malignant progression from benign nevi to melanoma. In addition, the importance of MAFG extends to fully transformed melanoma cells, as evidenced by the substantial reduction in proliferation following the silencing of MAFG in human melanoma cell lines. The stark contrast between the dispensability of MAFG in normal development and physiology and its requirement in melanoma cells – both in vitro and in vivo – identifies MAFG as a melanoma-specific vulnerability. Notably, while the oncogenic effects of MAFG were dependent on MITF and the phenotypic state, MAFG depletion consistently impacted melanoma cells in Melanocytic (WM164, SKMel28) and dedifferentiated (A375, WM793) cell states. This implies that the MAFG dependency operates via MITF-independent functions, and further work is needed to pinpoint the underlying molecular mechanism. Encouragingly, the broad effect of depletion suggests that therapeutic targeting of MAFG could be effective against melanomas regardless of their mutational or phenotypic profiles. Future studies investigating the effects of acute MAFG depletion on melanoma maintenance in vivo are needed to further explore this potential therapeutic approach. In parallel, emerging evidence points to a role for MAFG in modulating tumor-immune interactions in other tumor types^39–41^. Specifically, MAFG-silenced tumor cells in hepatocellular carcinoma and breast cancer are more susceptible to T cell-mediated cytotoxicity, which may further enhance its relevance as a therapeutic target. These findings suggest MAFG as a potential transcriptional integrator of immune modulation, an axis that may be explored in future studies.

In conclusion, we provide evidence for the robust oncogenic activity of the sMAF transcription factor MAFG in melanoma. MAFG influences the transcriptional output of MITF through direct interaction, representing an underexplored mechanism of MITF regulation that promotes phenotype switching. This study enhances our understanding of the molecular mechanisms driving melanoma progression, and further characterization of the processes affected by MAFG is expected to inform the development of improved therapeutic strategies.

## Methods

This study was conducted in accordance with all relevant ethical guidelines. All animal study protocols were approved by the University of South Florida (protocol IS00013445) and performed in compliance with IACUC standards for the ethical treatment of animals.

### Cell culture and treatments

The human immortalized melanocyte cell line Hermes1 was obtained from the Functional Genomics Cell Bank at St. George’s, University of London, UK, and cultured in RPMI-1640 media supplemented with 10% FBS, 10 ng/mL hSCF (R&D, Cat # 255-SC), 200 nM TPA (Sigma, Cat # P8139), 200 pM Cholera Toxin (Sigma, Cat # C8052), and 10 nM Endothelin-1 (Sigma, Cat # E7764) at 37 °C in a humidified atmosphere containing 10% CO_2_. A375 and SKMel28 cells were purchased from ATCC; WM164 and WM793 cells were a gift from M. Herlyn from the Wistar Collection of Melanoma cell lines. All melanoma cell lines were cultured in RPMI-1640 containing 5% FBS at 37 °C in a humidified atmosphere containing 5% CO_2_. Lenti-X HEK293T cells were obtained from Takara and cultured in DMEM containing 10% FBS at 37 °C in a humidified atmosphere containing 5% CO_2_. All cell lines were routinely tested for mycoplasma using MycoAlert Plus (Lonza, Cat # LT07-710), and human melanoma cell lines were STR authenticated by Moffitt’s Molecular Genomics Core.

### Plasmids

The CMV promoter and puromycin in pLenti-GFP-puro were replaced with the EF1α promoter and blasticidin, respectively, using standard In-Fusion cloning (Takara Bio, Cat. # 638911) to create pLEGB. The Myc-DDK-tagged ORF clone of MAFG (OriGene, Cat. # RC221486) was cloned into pLEGB to replace GFP and create pLEB-MAFG. ORF clones of MAFF and MAFK (OriGene, Cat. # RC215609L3 and #RC223543) were used to replace MAFG in pLEB-MAFG to create MYC-DDK-tagged cDNA expression constructs. TurboID-pCDNA constructs were obtained from E. Padron (Moffitt Cancer Center and Research Institute). MAFG and TurboID-3 x NLS were amplified via PCR from the Myc-DDK-tagged ORF MAFG expression plasmid and pCDNA3-TurboID-3 x NLS, respectively, and inserted into lentiviral vector pDEST via In-Fusion Recombination. shRNAs targeting MITF were designed using SplashRNA (http://splashrna.mskcc.org/) and cloned into a Doxycycline-inducible vector (pRRL-Puromycin) using the Q5 Site-Directed Mutagenesis Kit (NEB, Cat. # E0554S) with minor modifications. PCR for site-directed mutagenesis was carried out using 2X Platinum SuperFi II Green PCR Master Mix (Thermo Scientific, Cat. # 12369010), following the manufacturer’s recommended three-step protocol. To generate the MAFG-MITF dimer (M~M), the cDNA of the melanoma-specific isoform of MITF (M-MITF) was first cloned into pLenti-EF1α-Blasticidin, followed by cloning the MAFG cDNA downstream of MITF with a flexible linker of 22 amino acids [RS(GGGS)_4_GGRS] using Takara In-Fusion cloning. MAFG mutants (S124D, S124A, and delM100/L104A/L107A) and MITF mutant (L281A/L285A) were generated by Site-Directed Mutagenesis as described above.

### Cell transfection and lentiviral transduction

For siRNA transfections, 100,000 cells/well were plated in 6-well plates and transfected with 25 nM of ON-TARGETplus siRNA pool (Dharmacon, NFE2L2 Cat. # L-003755-00-0005; MITF Cat. # L-008674-00-0005; MAFG Cat. # L-009109-00-0005) or Non-Targeting control (Dharmacon, Cat. # D-001810-10-05) using JetPrime (VWR, Cat. # 89129-924) according to the manufacturer’s protocol. 4–6 h after transfection, cells were trypsinized and replated for cell biological assays. For lentiviral transductions, Lenti-X HEK293T cells were transfected with the lentiviral vector and the ∆8.2 and pMD2-VSV-G helper plasmids at a 9:8:1 ratio. Supernatants were collected 48 h after transfection and filtered through a 0.45 μm filter. Hermes1 and melanoma cells were plated in 10 cm dishes and transduced overnight with supernatants in the presence of 8 μg/mL polybrene. Selection was carried out by treating cells with 2.5–10 μg/mL Blasticidin for 5 days or 1 μg/mL Puromycin for 3 days.

### Proliferation and focus formation

For proliferation assays with Hermes1, cells were plated in 96-well plates at a density of 4500 cells/well and harvested for seven days. Cells were fixed and stained with 0.1% crystal violet (VWR, Cat. # 97061-850) solution in 20% methanol for 20 min followed by extraction of crystal violet with 10% acetic acid. Absorbance was measured at 600 nm using a plate reader. For melanoma proliferation assays, cells were plated in 96-well plates at a density of 1000−2500 cells/well in 200 μL of complete medium. After 24 h, the plate was loaded into Cellcyte-X live cell analyzer (ECHO). Images of each well were taken daily for 4–5 days, and the cell confluency of each image was quantified. For focus formation assays, cells were plated in 6-well or 12-well plates at a density of 1000−2000 cells/well and cultured for 2–3 weeks. Cells were fixed and stained with 0.1% crystal violet solution in 20% methanol for 20 minutes. Colonies or area covered was quantified using ImageJ software.

### RNA isolation and quantitative RT-PCR

Total RNA was isolated using TRI-Reagent (Zymo Research, Cat. # R2050-1-200) according to the manufacturer’s recommendations. For qRT-PCR, 500 ng of total RNA were reverse transcribed using PrimeScript RT Master Mix (Takara Bio, Cat. # RR036A), and subsequent SYBR Green-based qPCRs were performed as previously described^11^. Primers for SYBR Green qPCR were as follows: MAFG: FWD 5’-AGGAGATCGTCCAGCTGAAGCA-3’, REV 5’-TCTGCTTCTCCAGCTCCTCCTT-3’; NFE2L2: FWD 5’-CACATCCAGTCAGAAACCAGTGG-3’, REV 5’-GGAATGTCTGCGCCAAAAGCTG-3’; PRDX1: FWD 5’-CTGCCAAGTGATTGGTGCTTCTG-3’, REV 5’-AATGGTGCGCTTCGGGTCTGAT-3’; GAPDH: FWD 5’-GAGAGACCCTCACTGCTG-3’, REV 5’-GATGGTACATGACAAGGTGC-3’; MITF: FWD 5’-GGCTTGATGGATCCTGCTTTGC-3’, REV 5’-GAAGGTTGGCTGGACAGGAGTT-3’; AXL: FWD 5’-GTTTGGAGCTGTGATGGAAGGC-3’, REV 5’-CGCTTCACTCAGGAAATCCTCC-3’; TYR: FWD 5’-GCACAGATGAGTACATGGGAGG-3’, REV 5’-CTGATGGCTGTTGTACTCCTCC-3’; PMEL: FWD 5’-CTGCCTCAATGTGTCTCTGGCT-3’, REV 5’- CAAGGACCACAGCCATCAACAC-3’; MLANA: FWD 5’-GGACAGCAAAGTGTCTCTTCAAG-3’, REV 5’-TCAGGTGTCTCGCTGGCTCTTA-3’; MAFF: FWD 5’-ATCCCCTATCCAGCAAAGCTC-3’, REV 5’-TTGAGCCGTGTCACCTCCTC-3’; MAFK: FWD 5’-CGACTAATCCCAAACCGAAT-3’, REV 5’-ACATGGACACCAGCTCATCA-3’.

### RNA-sequencing

Total RNA from cells was isolated using miRNeasy Mini Kit (Qiagen, Cat. # 217004), quantitated with the Qubit Fluorometer (ThermoFisher Scientific), and screened for quality on the Agilent TapeStation 4200 (Agilent Technologies). The samples were then processed for RNA-sequencing using the NuGEN Universal RNA-Seq Library Preparation Kit with NuQuant (Tecan Genomics, Cat. # 9156). Briefly, 100 ng of RNA was used to generate cDNA and a strand-specific library following the manufacturer’s protocol. Quality control steps were performed, including TapeStation size assessment and quantification using the Kapa Library Quantification Kit (Roche, Cat. # 07960140001). The final libraries were normalized, denatured, and sequenced on the Illumina NovaSeq 6000 sequencer with the SP-200 cycle reagent kit in order to generate approximately 50 million 100-base read pairs per sample (Illumina). The raw RNA-seq reads were first assessed for quality using FastQC (http://www.bioinformatics.babraham.ac.uk/projects/fastqc/). Quality trimming was performed using cutadapt^42^ to remove reads with adapter contaminants and low-quality bases. Read pairs with either end too short ( < 25 bp) were discarded from further analysis. Next, trimmed and filtered reads were aligned to the human transcriptome GRCh38 using STAR^43^. Uniquely aligned reads were counted at the gene level using featureCounts^44^ and then normalized using DESeq2 package^45^ taking into account RNA composition bias. A negative binomial generalized linear model implemented in DESeq2 were used to determine differentially expressed genes. Genes with Fold Change > 2 and false discovery rate (FDR) controlled p-value ≤ 0.05 were considered differentially expressed and visualized using a volcano plot and heatmap. The gene list was used to perform pre-ranked gene set enrichment analysis (GSEA^46^ version 4.0.2) to assess enrichment of hallmarks, curated gene sets, and gene ontology^47^ terms in MSigDB^46,48^. We also collected signatures for phenotypic states of melanoma cells^19^ and assessed their enrichment in MAFG overexpression vs. control samples using pre-ranked GSEA. The resulting normalized enrichment score (NES) and FDR-controlled *p*-values were used to assess transcriptome changes. RNAseq data of melanoma cell lines^11^ (GSE148552) were analyzed as described above. Gene expression was quantified as Transcript per Million (TPM) using RSEM^49^. Enrichment of the phenotypic states of melanoma cells^19^ was calculated in each sample by single-sample GSEA analysis using the GSVA R package. The z-scored enrichment scores were visualized using a heatmap.

### CUT&RUN

At ~ 80% confluency, adherent cells were trypsinized, washed with PBS, and counted. Isolation of cell nuclei from a minimum of 250,000 cells, and experimental procedures were performed as described using the CUTANA ChIC/CUT&RUN Kit version 4.0 (EpiCypher, Cat. # SKU: 14-1048). Antibodies used were MAFG (1 µl/reaction, Abcam, Cat. # ab154318) and MITF (5 µl/reaction, Millipore Sigma, Cat. # HPA-003259). Following enrichment using the EpiCypher CUT&RUN kit, the samples were quantitated with the Qubit dsDNA HS Assay Kit (Thermo Fisher Scientific, Cat. # Q32851), and 0.6 to 5 nanograms of enriched DNA was used for library preparation using the Kapa HyperPrep Kit (Roche, Cat. # 07962347001) following EpiCypher’s parameters for indexing PCR and library amplification as described in the CUT&RUN Library Prep Manual. The final libraries were Qubit quantitated and screened on the Agilent TapeStation D1000 ScreenTape (Agilent Technologies) to assess the fragment size distribution. Following final quantification using the Kapa Library Quantification Kit (Roche, Cat. # 07960140001), the libraries were sequenced on the NextSeq 500 using a Mid-output 150-cycle Kit in 2×50 configuration to generate 6–8 million read-pairs per sample (Illumina). *FastQC* was used to examine characteristics of the sequencing libraries. Sequence reads of verified libraries were aligned to the human transcriptome GRCh38 using Bowtie2^50^. BigWig files were generated using *bamCoverage* from deepTools^51^ and visualized in IGV. Model-based Alignment (MACS2)^52^ was utilized for peak calling with --q 0.01 using IgG samples as control. A consensus set of peaks was generated by aggregating peaks of individual samples. The human ENCODE blacklist regions^53^ were removed from consensus peaks. Peaks were annotated using *annotatePeak* function in R package ChIPseeker^54^. The number of reads mapped per consensus peak were calculated for each sample using the *bamsToCount* function in Rsubread^55^ R package. Raw counts were further normalized by library size and tested for differential expression using DESeq2^45^. Differential peaks were identified as fold-change > 2 and false discovery rate (FDR) controlled *p*-value < 0.05. Pathway analysis^56^ was further performed using genes harboring differentially expressed peaks. Bubble plots were created using the SRplot tool^57^.

### Motif analysis

Motif enrichment analysis was performed using the Regulatory Sequence Analysis Tools (RSAT)^58^. Cut&Run–seq peaks were grouped into three categories (MITF-only, MAFG-only, and MITF/MAFG co-bound) based on binding status under control conditions. The same peak definitions were analyzed independently for siNT and GFP conditions. For each peak group and condition, genomic sequences centered on peak summits were provided as input to RSAT peak-motifs. Analyses were run with a maximum sequence length of 800 bp, using first-order Markov models for background correction during both motif discovery and scanning. Motif discovery was performed using oligonucleotide- and position-based approaches, identifying up to eight motifs per dataset with oligonucleotide lengths ranging from 6 to 8 bp. Motifs discovered at similar lengths were merged. Sequences were aligned relative to the peak center, and overlapping motifs were excluded.

### Public single-cell RNAseq data analysis

We downloaded processed single-cell RNAseq data of mouse melanoma^19^ from https://marinelab.sites.vib.be/en, and processed single-cell RNAseq data of malignant cells of human melanoma metastatic biopsies^20^ from KU Leuven RDR (10.48804/GSAXBN). Normalized expression of MAFG was visualized on the UMAP generated by the original studies. Differential expression of MAFG across different melanoma states of malignant cells was calculated by the Wilcoxon signed-rank test, and visualized by log2FC and percentage of expression.

### Biotin proximity labeling

TurboID-MAFG and TurboID-NLS expressing cells were cultured in DMEM containing 5% dialyzed FBS at 37 °C. For biotin labeling, cells were grown in three 10 cm dishes per experimental condition for 24 h and were given fresh media the day before the addition of biotin. At 80–90% confluency, cells were incubated with fresh media and 50 µM biotin in 5% dialyzed FBS in DMEM for 10 min at 37 °C. Biotin labeling was stopped by immediately placing cells on ice and five washes with PBS. Cells were scraped and collected to confirm biotinylation of proximal proteins as described previously^59^.

*Sample preparation for mass spectrometry analysis:* A total of 6 samples were analyzed, consisting of *n* = 3 biological replicates for the TurboID-MAFG experimental condition and *n* = 3 biological replicates for the TurboID-NLS spatial negative control. Cells were sonicated at 4 °C in RIPA buffer (10% glycerol, 50 mM HEPES, 150 mM NaCl, 2 mM EDTA, 0.1% SDS, 1% Triton X-100, 0.2% Sodium deoxycholate) containing protease inhibitor (Thermo Scientific, Cat. # 78429), phosphatase inhibitor (Thermo Scientific, Cat. # 78426), and benzonase (Sigma, Cat. # E1014-5KU). Protein concentration was measured using Pierce BCA Protein Assay kit (Thermo Scientific, Cat. # 23225). 4.5 mg of protein lysate was incubated with 30 µL of packed pre-washed Streptavidin Sepharose beads (Cytiva, Cat. # 17511301) overnight on a rotator at 4 °C. Beads were washed with wash buffer (WB) 1 (2% SDS) twice, once with WB2 (500 mM NaCl, 0.1% deoxycholate, 1% Triton X-100, 1 mM EDTA, 50 mM HEPES, pH 7.5), once with WB3 (250 mM LiCl, 0.5% Triton X-100, 0.5% deoxycholate, 1 mM EDTA, 50 mM HEPES. pH 8.1), and once with WB4 (150 mM NaCl, 50 mM HEPES, pH 7.4). The streptavidin beads were further washed 3 times in 50 mM ammonium bicarbonate (ABC). Beads were then resuspended in 100 µL of 50 mM ABC containing 1 mg trypsin (Promega, Cat. # V5113) and 0.1 mAu Lys-C (Wako Chemicals, Cat. # 129-02541) and incubated overnight at 37 °C with shaking for on-bead-digestion. The following day, 0.5 mg trypsin and 0.05 mAu Lys-C were added to the beads and incubated for 2 hours. Digested peptides in the supernatant were collected into a fresh tube, and the beads were washed twice with HPLC-grade water and pooled with the peptides. Pooled peptides were centrifuged at 16,000 × *g* for 10 min and filtered using BioPureSPN columns (Nest Group, Cat. # C100500), pre-wetted with 0.1% trifluoroacetic acid, and centrifuged at 3000 × *g* for 2 min. Filtered peptides were acidified to 2% formic acid, dried using a speed vac, and stored at − 80 °C. Peptides were resuspended in 13 µL of 98 parts buffer A (water + 0.1% formic acid), and 2 parts buffer B (99.9% acetonitrile + 0.1% formic acid) and 5 µL of peptides were injected for mass spectrometric analysis.

#### Chromatographic separation and label-free quantification

Tryptic peptides were separated by reverse phase nano-HPLC using an Ultimate 3000 RSLCnano System (Thermo Fisher Scientific) with a uPAC Trapping column (Thermo Scientific) and a 50 cm uPAC Neo HPLC column (Thermo Scientific). For peptide separation and elution, mobile phase A was 0.1% formic acid (FA) in water and mobile phase B was 0.1% FA in acetonitrile. Peptides were injected onto the trap column at 10 µL/min for 3 min using the loading pump. Initially, the nanoflow rate was set at 0.75 µL/min and 2% mobile phase B while the peptides were loaded onto the trap column, at 2.8 min the solvent composition was changed to 10% mobile phase B. At 5 min the flow rate was dropped to 0.300 µL/min at 12% mobile phase B. A two-step gradient was used from 12% to 20% mobile phase B for 41.8 min followed by 20% to 40% mobile phase for 15.9 min. The flow rate was then increased to 0.750 µL/min for column washing using seesaw gradients and re-equilibration. Mass spectrometry analysis was performed on an Orbitrap Eclipse (Thermo Fisher Scientific) operated in data-dependent acquisition mode. The MS1 scans were acquired in Orbitrap at 240k resolution, with a 1 × 10^6^ automated gain control (AGC) target, auto max injection time, and a 375–2000 m/z scan range. MS2 targets were filtered for charge states 2-7, with a dynamic exclusion of 60 s, and were accumulated using a 0.7 m/z quadrupole isolation window. MS2 scans were performed in the ion trap at a turbo scan rate following higher energy collision dissociation with a 35% normalized collision energy. MS2 scans used a 1 × 10^4^ AGC target and 35 ms max injection time.

#### Protein identification and data filtering for sample comparison

Raw MS data files were processed for protein identification and label-free quantification (LFQ) by MaxQuant (version 2.4.1.0)^60^ using the Human SwissProt canonical sequence database (3AUP000005640, downloaded June 2023) and common contaminants (streptavidin, trypsin, albumin and the default MaxQuant contaminants). The following parameters were used: specific tryptic digestion up to four missed cleavages, variable modification search for up to 5 modifications per peptide, including carbamidomethyl cysteine, protein N-terminal acetylation, and methionine oxidation, default match between run parameters and label-free quantification with a minimum ratio count of 1. Only unmodified, oxidized or N-terminal acetylated unique peptides were used for protein quantification. Quantified protein intensity data from MaxQuant were imported and analyzed by Perseus (version 2.0.11)^61^. Data were first filtered based on the categorical column to remove proteins labeled as “Only identified by site”, “Reverse”, and “Potential contaminant”. Then Intensity values were log2 transformed, and replicates were grouped in Categorical annotation rows. Data were further filtered to remove proteins without three valid values in at least one group. Missing values were replaced with 1% limit of detection for the total matrix. MAFG vs. control groups were compared using a two-sided *t* test with FDR of 0.05 and S0 of 0 with the default permutation-based FDR correction for multiple t-tests.

### Proximity ligation assay (PLA)

Cells were plated in an 8-well Chamber Slide system (Thermo Fisher Scientific Cat. # 154534PK) at a density of 50,000 cells per well. After 16–24 h, each well was washed twice with PBS and fixed with 100% methanol at − 20 °C for 20 min. After methanol fixation, cells were washed twice with PBS and PLA was performed with the Duolink In Situ PLA (Sigma Aldrich, Cat. # DUO92008) following the manufacturer’s indications. For the TMA, amplification was performed for 2 h. Antibodies used were MAFG (Abcam, Cat. # ab154318) and MITF (Santa Cruz, Cat. # sc-56725) at 1:400 for cell lines and at 1:200 for the TMA, with PLA anti-rabbit PLUS (Sigma Aldrich, Cat. # DUO92002) and PLA anti-mouse MINUS (Sigma Aldrich, Cat. # DUO92004) probes. All images were taken at 40x by confocal microscopy, and the maxIP projections were analyzed. For each image, total foci and nuclear foci per cell were counted.

### Immunoblotting

Cells were washed and scraped in PBS, centrifuged, and the pellet was lysed using RIPA buffer containing protease and phosphatase inhibitor cocktail (Thermo Scientific, Cat. # 78440). 20 μg of total protein were subjected to SDS-PAGE and Western blot, performed as previously described^15^. Primary antibodies used were MAFG (Thermo Fisher Scientific, Cat # PA5-90907 and Abcam, Cat # AB154318), AXL (Cell Signaling, Cat.no 8661S), MITF (Cell Signaling, Cat.no 12590S), Flag-M2 (Millipore Sigma, Cat # F1804), HSP90 (Cell Signaling, Cat # 4874), Anti-V5 (Thermo Fisher Scientific, Cat # R96025; RRID: AB_2556564), p-Ser/Thr (Cell Signaling, Cat # 9631S), and β-Actin (Invitrogen, Cat. # AM4302). Streptavidin-HRP (Thermo Fisher Scientific, Cat # S911) was used to detect biotinylated proteins in the TurboID biotin proximity ligation experiment.

### Co-Immunoprecipitation

Pierce protein A/G magnetic beads (Thermo Fisher, Cat. # 88803) were washed with 1% filtered BSA-PBS for 1 hr at 4 °C with agitation. Cells were washed once with PBS, scraped, and centrifuged to collect cell pellets. Cell pellets were lysed using 200 µl EBC lysis buffer (50 mM Tris-HCL, pH 7.4; 150 mM NaCl; 0.5% IGEPAL, 1:1000 Halt protease and phosphatase inhibitor cocktail (Thermo Fisher, Cat. #78440)), incubated at 4 °C for 30 minutes with agitation, and centrifuged at 12,000 × *g* for 20 min to collect the supernatant. Protein concentration was determined by the DC protein assay. Pre-washed beads were incubated with antibodies (Normal Rabbit IgG, Millipore Sigma, Cat. #12-370; Normal mouse IgG, Cell Signaling Technology, Cat. #68860 L; MAFG, Abcam, Cat. # ab154318; MITF, Millipore Sigma, Cat. # HPA-003259; Flag-M2, Millipore Sigma, Cat # F1804; V5, Thermo Fisher Scientific, Cat # R96025) for 2 h at RT with end-over-end rotation. 0.5-1 mg protein lysate was added to antibody-coated beads and incubated overnight at 4 °C with end-over-end rotation. Beads were washed 3 times using lysis buffer and once using ice-cold sterile PBS. Magnetically separated beads were resuspended in 20 µl 1 x Laemmli buffer and incubated at 350 rpm at RT for 2 min to elute protein. Supernatant was then subjected to immunoblotting.

### Immunohistochemistry

Tumor tissues were fixed in 10% buffered formalin overnight and dehydrated in 70% ethanol. Tissues were paraffin-embedded, sectioned, and hematoxylin and eosin-stained by IDEXX BioAnalytics (Columbia, MO). The tissue sections were de-paraffinized in xylene and rehydrated through an alcohol series. Antigen retrieval was performed by heating the sections in citrate buffer (pH 6.0) for 10 min followed by blocking endogenous peroxidase activity with 3% hydrogen peroxide. Immunohistochemistry was performed using ImmPRESS HRP goat anti-rabbit kit (Vector Laboratories, Cat. # MP-7451) as per the manufacturer’s instructions and then incubated with DAB peroxidase substrate (Cat. # SK4105). The tissue sections were then counter stained in hematoxylin (Vector Laboratories, Cat. # H-3404). Antibodies against MAFG (Abcam, Cat. # ab154318), SOX10 (Cell Signaling, Cat. # 78330), MITF (Cell Signaling, Cat. # 12590), and Ki67 (Cell Signaling, Cat. # 12202S) were used for immunohistochemistry.

### Multiplex immunohistochemistry (mIHC)

Mouse melanoma tissues were stained for SOX2 (1:100, Cell Signaling 14962), AXL (1:100, Cell Signaling 8661), and DAPI with an Automated Opal 7-Color IHC Kit and quantified in Moffitt’s Advanced Analytical and Digital Laboratory:

#### Multiplex immune panel procedure

Formalin-fixed and paraffin-embedded (FFPE) tissue samples were immunostained using the AKOAYA Biosciences OPAL 7-Color Automation IHC kit (Waltham, MA) on the BOND RX autostainer (Leica Biosystems, Vista, CA). The OPAL 7-color kit uses tyramide signal amplification (TSA)-conjugated to individual fluorophores to detect various targets within the multiplex assay. Sections were baked at 65 °C for one hour and then transferred to the BOND RX (Leica Biosystems). All subsequent steps were performed using an automated OPAL IHC procedure (AKOYA). OPAL staining of each antigen occurred as follows: heat induced epitope retrieval was achieved with EDTA pH 9.0 buffer for 20 min at 95 °C before the slides were blocked with AKOYA blocking buffer for 10 min. Then slides were incubated with primary antibody at RT for 30 min followed by OPAL HRP polymer and one of the OPAL fluorophores during the final TSA step. Individual antibody complexes are stripped after each round of antigen detection. This was repeated once using the remaining antibody of the panel. After the final stripping step, DAPI counterstain was applied to the multiplexed slide and was removed from BOND RX for coverslipping with ProLong Diamond Antifade Mountant (ThermoFisher Scientific). Autofluorescence slides (negative control) were included, which used primary and secondary antibodies omitting the OPAL fluorophores and DAPI. All slides were imaged with the Phenolmager HT (Akoya Biosciences).

#### Quantitative image analysis

Multi-layer TIFF images were exported from InForm (AKOYA) and loaded into HALO Image Analysis Platform version 4.0 (Indica Labs, New Mexico) for quantitative image analysis. For the quantitative fluorescent phenotype analysis, tissues were segmented into individual cells using the DAPI marker. For each marker, a positivity threshold within the nucleus or cytoplasm is determined based on visual intensity and expected staining localization. After setting a positive fluorescent threshold for each staining marker, the entire image set is analyzed with the created algorithm. The generated data includes positive cell counts for each fluorescent marker in cytoplasm or nucleus, and percent of cells positive for the marker.

### ESC-GEMM models and in vivo experiments

All animal experiments were conducted in accordance with an IACUC protocol approved by the University of South Florida. Mice were housed in 12 h light/dark cycles, at approximately 24 °C and 55% humidity. ES cell targeting by recombination-mediated cassette exchange and generation of chimeras was performed as described previously^15^. ES cell lines on a C57BL/6 background with the following genotypes were targeted with TRE-MAFG or TRE-GFP constructs for this study: Braf^V600E^; Pten^FL/WT^; Tyr-CreERt2; CAGs-LSL-rtTA3; CHC (BP), Braf^V600E^; Cdkn2a^FL/FL^; Tyr-CreERt2; CAGs-LSL-rtTA3; CHC (BCC), and Pten^FL/FL^; Tyr-CreERt2; CAGs-LSL-rtTA3; CHC (PP). In addition, Braf^V600E^; Pten^FL/FL^; Tyr-CreERt2; CAGs-LSL-rtTA3; CHC (BPP) ES cells on a C57BL/6 background were electroporated with Cas9 protein and 3 sgRNAs targeting murine *Mafg* (Synthego, Gene Knockout Kit v2_mouse_Mafg) to produce MAFG knockout BPP mice. Cas9 transfection decreased the pluripotency of BPP ES cells, yielding lower contribution chimeras (10–20%) in which tumorigenesis was analyzed. Melanoma development was induced in 3–4-week-old male and female MAFG (*n* = 43) and GFP control (*n* = 36) chimeras having similar ES cell contribution using 25 mg/mL 4-OH Tamoxifen as described previously^15^. Mice were fed 200 mg/kg Doxycycline (Envigo, Cat. # TD180625) *ad libitum*. Experimental mice were euthanized when IACUC-approved clinical endpoints, typically volume of primary tumors (1500 mm^3^), was reached. Maximal tumor size of 1500 mm^3^ was not exceeded. NSG mice were obtained from JAX (Stock No: 005557) and bred in-house. 6-week-old male and female NSG mice were randomly divided into groups (at least 5 mice per group). 400,000 WM164 or 200,000 SKMel28 melanoma cells were subcutaneously injected into NSG mice, and tumor growth was measured with calipers every 2-3 days. Experimental mice were euthanized when an IACUC-approved clinical endpoint, either a primary tumor volume of 1500 mm^3^ or significant worsening of animal wellbeing, was reached. Sex was not included as a biological variable in in vivo experiments, as tumor latency and growth kinetics were comparable between male and female mice. Data are therefore presented as pooled of both male and female mice.

### Statistics & reproducibility

Sample sizes were chosen based on prior studies and exploratory aims. Data were assessed for quality, and no data were excluded from the analyses. The experiments were not randomized, and the investigators were not blinded to allocation during experiments and outcome assessment. No statistical method was used to predetermine sample size. Statistical analysis was performed using GraphPad Prism software v9.0. Survival data were compared by applying the Gehan-Breslow-Wilcoxon test, and all other data were analyzed with the unpaired two-tailed *t* test or ordinary one-way ANOVA. A p-value below 0.05 (n.s., not significant; *p < 0.05; **p < 0.01; ***p < 0.001; ****p < 0.0001) was considered statistically significant. Data represent the mean ± SD of at least two independent biological experiments.

### Reporting summary

Further information on research design is available in the Nature Portfolio Reporting Summary linked to this article.

## Supplementary information

Supplementary Information

Description of Additional Supplementary Files

Supplementary Dataset 1

Supplementary Dataset 2

Supplementary Dataset 3

Supplementary Dataset 4

Reporting Summary

Transparent Peer Review file

## Source data

Source Data

## Data Availability

Sequencing data supporting the findings of this study have been deposited in the Gene Expression Omnibus under accession numbers GSE274945 (RNA-seq of MAFG overexpression in WM164, SKMel28, A375 cells), GSE274811 (RNA-seq of MAFG ~ MITF dimer), and GSE274810 (CUT&RUN). Mass spectrometry proteomics data have been deposited to the ProteomeXchange Consortium via PRIDE and can be accessed through the accession number PXD055213. Previously published datasets (GSE3189 and GSE98394) are available at GEO under https://www.ncbi.nlm.nih.gov/geo/query/acc.cgi?acc=GSE3189 and https://www.ncbi.nlm.nih.gov/geo/query/acc.cgi?acc=gse98394. Source data are provided with this study. Source data are provided in this paper.

## Code availability

No custom codes were developed and used in this study.
