# Supplementary material for: A MAFG~MITF complex drives melanoma phenotype switching and progression
Source: Nat Commun. 2026 May 21;17:6685. doi: 10.1038/s41467-026-73291-x (PMC13385793; doi:10.1038/s41467-026-73291-x)

SUPPLEMENTARY FIGURE LEGENDS

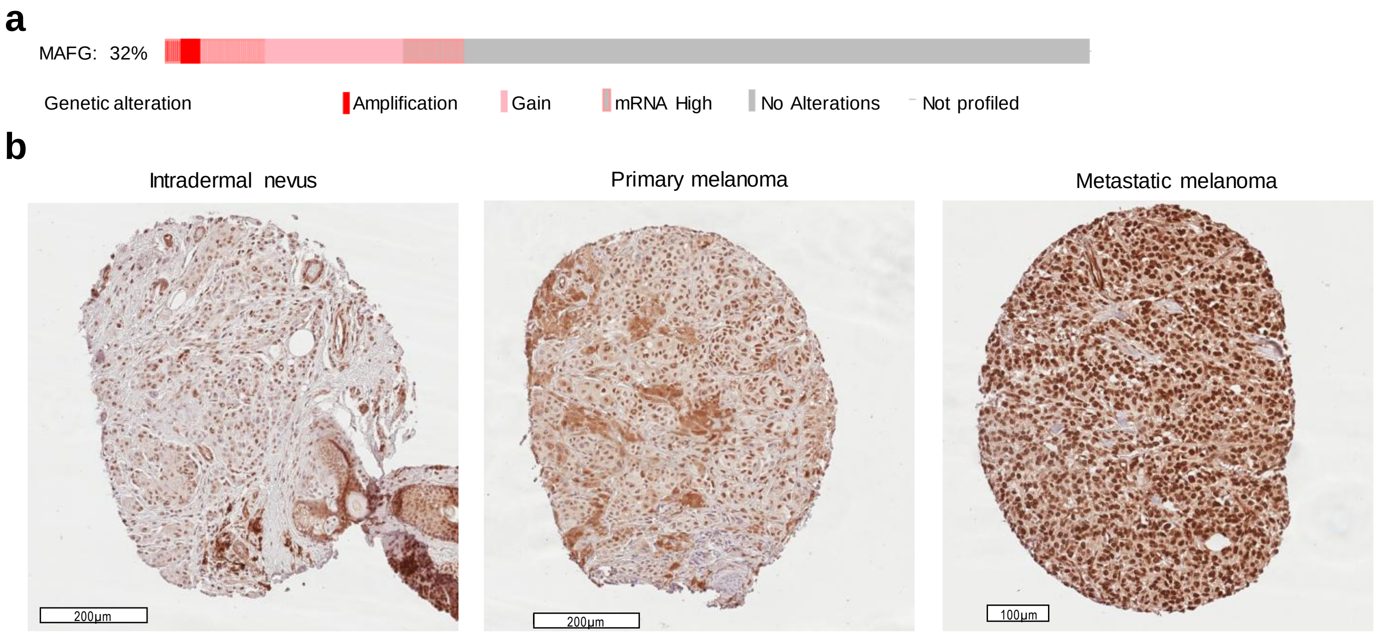

Supplementary Figure 1: MAFG is overexpressed in human melanoma. (**a**) Oncoprint of MAFG copy number gains and mRNA expression in samples from The Cancer Genome Atlas skin cutaneous melanoma (TCGA-SKCM) dataset. (**b**) Representative MAFG immunohistochemistry images on TMA cores containing intradermal nevus, primary melanoma, or metastatic melanoma. Scale bars indicate 200µm for Intradermal nevus and Primary melanoma, and 100µm for Metastatic melanoma.

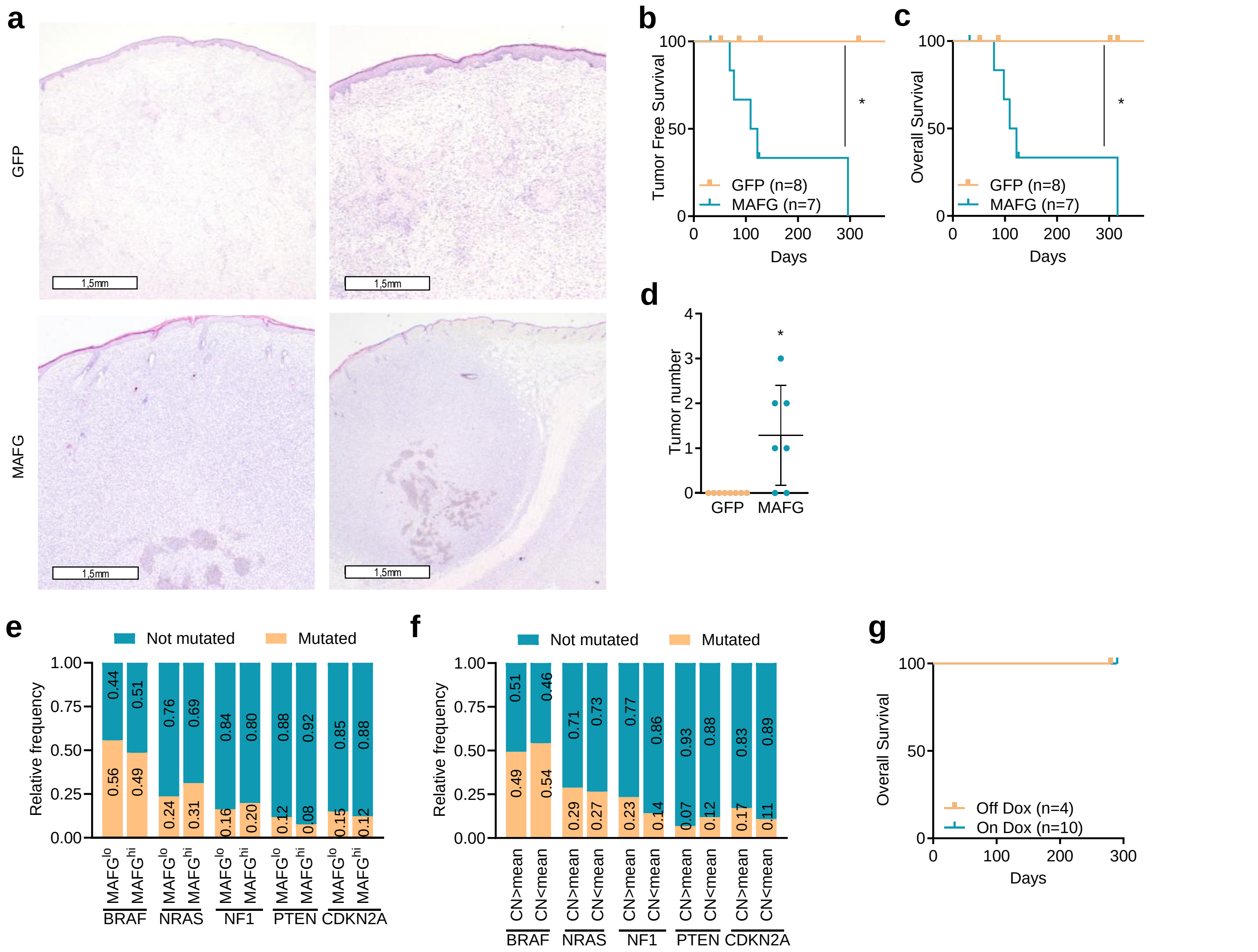

Supplementary Figure 2: MAFG overexpression elicits oncogenic effects in spontaneous mouse melanoma models. (**a**) Representative low magnifications H&E images of melanomas from BP^{Mafg} and BP^{GFP} mice. Scale bars indicate 1.5mm. (**b,c**) Kaplan–Meier curves showing the tumor-free survival (b, p=0.0021) and overall survival (c, p=0.0021) of BCC^{GFP} (n = 8) and BCC^{MAFG} (n = 7) chimeras using the Gehan-Breslow-Wilcoxon test. (**d**) Number of melanomas that developed in the BCC^{GFP} (n = 8) and BCC^{MAFG} (n = 7) chimeras. Statistical significance was determined using Welch's two-tailed t-test (p=0.0223). Error bars represent mean ± s.d. (**e,f**) Correlation between MAFG overexpression (e) or copy number alterations (f) and the frequency of *BRAF*, *NRAS*, *NF1*, *CDKN2A*, and *PTEN* alterations obtained from the SKCM-TCGA dataset. (**g**) Kaplan–Meier curves comparing the tumor free survival of PP^{MAFG} mice fed a Doxycycline-containing diet (On Dox, n = 10) or a regular diet (Off Dox, n = 4) using the Gehan-Breslow-Wilcoxon test (p>0.999). ns, not significant; * p < 0.05; ** p < 0.01.

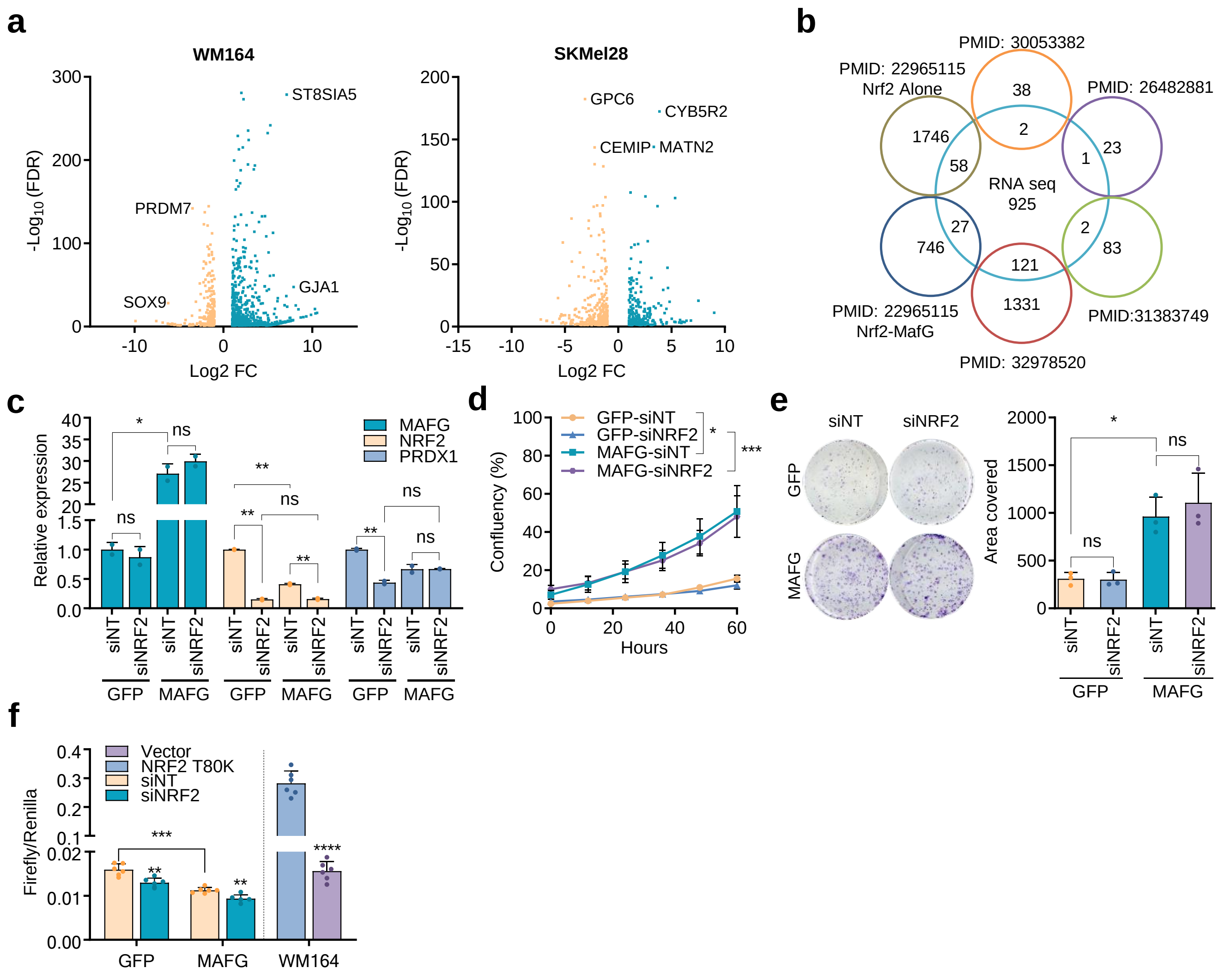

Supplementary Figure 3: The oncogenic effect of MAFG is independent of NRF2. (**a**) Volcano plots showing differentially expressed genes from RNA sequencing comparing GFP- and MAFG-overexpressing WM164 (left) and SKMel28 (right) cells. (**b**) Overlap between the 925 differentially expressed genes identified in WM164 cells upon MAFG overexpression and published NRF2 gene-expression signatures. (**c**) qRT-PCR measurement of MAFG, NRF2, and the canonical NRF2 target PRDX1 in WM164 cells overexpressing MAFG (n=2) or GFP (n=2) following NRF2 silencing. Cells transfected with non-targeting siRNA (siNT) served as controls. Data are from one representative biological experiment (of two independent experiments), measured in duplicate, and are presented as mean ± s.d. Statistical significance was assessed using a two-sided Welch's t-test. Exact p-values: • MAFG: GFP-siNT vs MAFG-siNT, p=0.039; GFP-siNT vs GFP-siNRF2, p=0.5027; MAFG-siNT vs MAFG-siNRF2, p=0.2987; GFP-siNRF2 vs MAFG-siNRF2, p=0.0232. • NRF2: GFP-siNT vs MAFG-siNT, p=0.0040; GFP-siNT vs GFP-siNRF2, p=0.0019; MAFG-siNT vs MAFG-siNRF2, p=0.0040; GFP-siNRF2 vs MAFG-siNRF2, p=0.7252. • PRDX1: GFP-siNT vs MAFG-siNT, p = 0.0932; GFP-siNT vs GFP-siNRF2, p=0.0084; MAFG-siNT vs MAFG-siNRF2, p=0.9675; GFP-siNRF2 vs MAFG-siNRF2, p=0.0635. (**d,e**) Proliferation (d) and focus-formation (e) assays in WM164 cells overexpressing MAFG or GFP after NRF2 silencing. Proliferation: n=3 technical replicates from one representative biological experiment (of two independent experiments); mean ± s.d.; two-sided Welch's t-test at each time point. Endpoint (60 h) p-values: GFP-siNT vs MAFG-siNT, p=0.0439; GFP-siNT vs GFP-siNRF2, p=0.0960; MAFG-siNT vs MAFG-siNRF2, p=0.8024; GFP-siNRF2 vs MAFG-siNRF2, p=0.0003. Focus formation: n=3 technical replicates from one representative biological experiment (of two independent experiments); mean ± s.d.; two-sided Welch's t-test. p-values: GFP-siNT vs MAFG-siNT, p=0.0219; GFP-siNT vs GFP-siNRF2, p=0.8953; MAFG-siNT vs MAFG-siNRF2, p=0.5408; GFP-siNRF2 vs MAFG-siNRF2, p=0.0393. (**f**) Luciferase assay using the 6×ARE luciferase reporter in WM164 overexpressing MAFG or GFP after NRF2 silencing (left), and parental WM164 overexpressing constitutively active NRF2-T80K as a positive control (right). n=6 technical replicates from one representative biological

experiment (of two independent experiments); mean ± s.d.; two-sided Welch's t-test. Exact p-values: GFP-siNT vs MAFG-siNT, p=0.0001; GFP-siNT vs GFP-siNRF2, p=0.0020; MAFG-siNT vs MAFG-siNRF2, p=0.0001; GFP-siNRF2 vs MAFG-siNRF2, p=0.0018; WM164-Vector vs WM164-NRF2-T80K, p<0.0001.
ns, not significant; *p<0.05; **p<0.01; ***p<0.001.

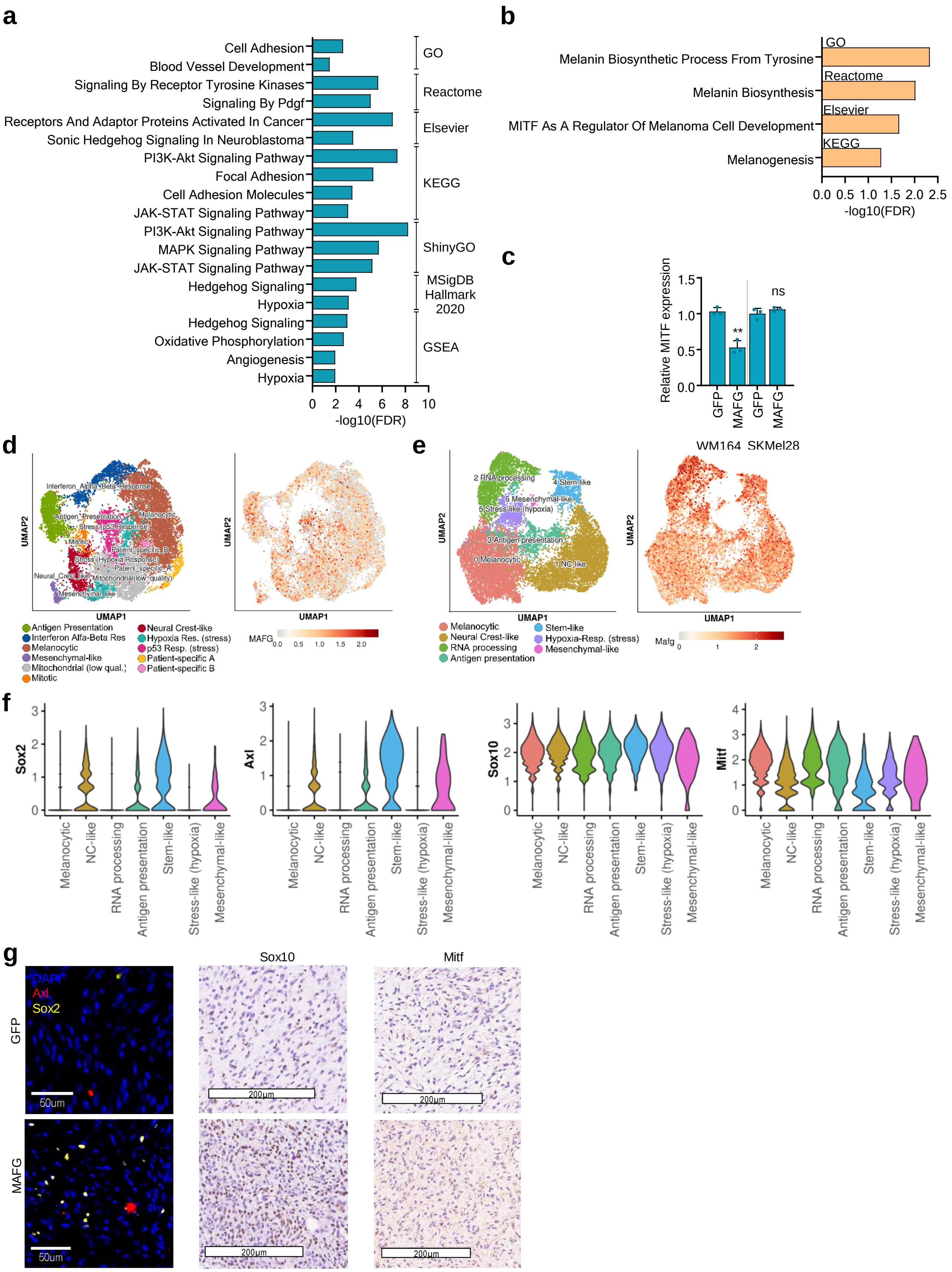

Supplementary Figure 4: MAFG promotes a phenotype switch. (**a,b**) Pathway analysis of the upregulated (a) and downregulated (b) genes identified by RNA sequencing comparing GFP and MAFG overexpression in WM164 cells. (**c**) qRT-PCR showing *MITF* expression upon MAFG overexpression in WM164 (p=0.0037) and SKMel28 cells (p=0.2992). n = 3 technical replicates from one out of two biological replicates is shown represented. Statistical significance was determined using Welch's two-tailed t-test. Error bars represent mean + s.d. (**d**) UMAP visualization of human melanoma cell clusters defined by cell-state gene signatures, illustrating the spatial distribution of MAFG expression across phenotypically distinct melanoma cell populations from Pozniak et al., 2024. (**e**) UMAP visualization of murine melanoma cell clusters defined by

cell-state gene signatures, illustrating the spatial distribution of MAFG expression across phenotypically distinct mouse melanoma cell populations from Karras et al., 2022. (**f**) Violin plots showing expression levels of *Sox2*, *Axl*, *Sox10* and *Mitf* in the murine melanoma cell populations from (Karras et al., 2022). (**g**) Multiplexed immunohistochemistry image of Sox2 and Axl (left, scale bars = 50µm) and immunohistochemistry of Sox10 and Mitf (right, scale bars = 200µm) in tumors from BP^{Mafg} and BP^{GFP} mice. ns, not significant; * $p < 0.05$; ** $p < 0.01$.

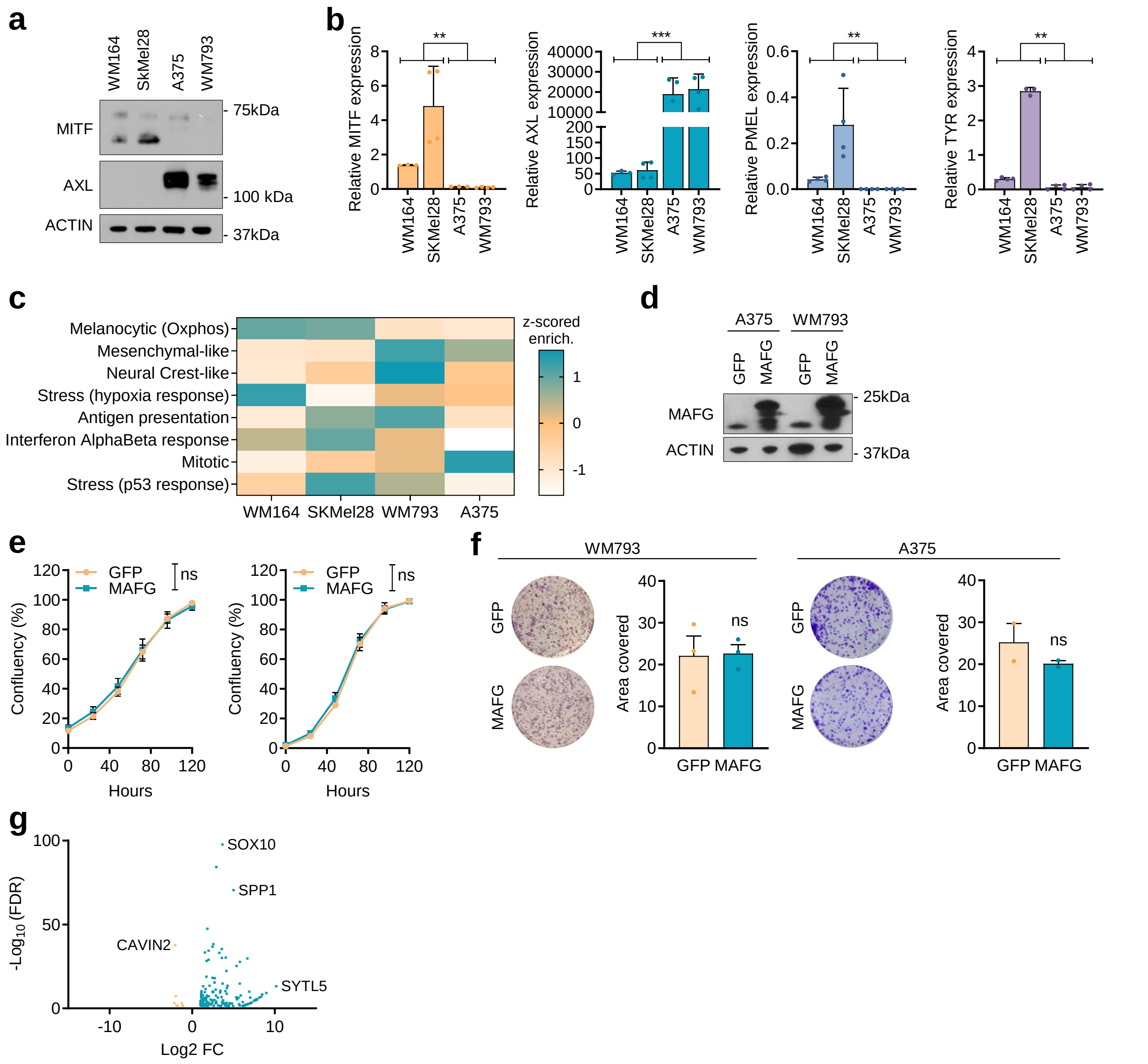

Supplementary Figure 5: MAFG has no oncogenic effects in dedifferentiated melanoma cells with low MITF expression. (**a**) Western blot showing levels of MITF and AXL in two MITF^{hi} (WM164 and SKMel28) and two MITF^{lo} (A375 and WM793) melanoma cell lines. Blot shown represents one out of three experiments with similar results. (**b**) qRT-PCR showing expression of *MITF*, *AXL*, and the MITF target genes *PMEL* and *TYR* in two MITF^{hi} (WM164 and SKMel28) and two MITF^{lo} (A375 and WM793) melanoma cell lines. Data shown are from n = 4 technical replicates from one out of two biological replicates. Statistical significance was determined using Welch's two-tailed t-test. p-values for each comparison are as follows: i) *MITF*: MITF^{hi} vs MITF^{lo+} p=0.0130; ii) *AXL*: MITF^{hi} vs MITF^{lo} p=0.0001; iii) *PMEL*: MITF^{hi} vs MITF^{lo+} p=0.0276; iv) *TYR*: MITF^{hi} vs MITF^{lo+} p=0.0418. Error bars represent mean + s.d. (**c**) Heatmap of the phenotypic state distribution of two MITF^{hi} (WM164 and SKMel28) and two MITF^{lo} (A375 and WM793) melanoma cell lines obtained by analyzing previously published RNAseq datasets (Vera et al., 2021) (GSE148552). (**d**) Western blot validating MAFG overexpression in A375 and WM793 melanoma cells. Ectopic MAFG is shifted up due to the presence of a Myc-DDK tag. (**e,f**) Proliferation (e) and focus formation (f) assays in WM793 (left) and A375 (right) cells overexpressing MAFG or GFP. For proliferation assay, n = 5 technical replicates from one out of two biological replicates are represented. Statistical significance was determined using Welch's two-tailed t-test for each time point. p-value of each comparison at 120h is as follows: A375-GFP vs -MAFG p= 0.7030; WM793-GFP vs -MAFG p=0.1422. Error bars represent mean ± s.d. For focus formation assay, n = 3 technical replicates from one out of two biological replicates are represented. Statistical significance was determined using Welch's two-tailed t-test. p-value of each comparison is as follows: A375-GFP vs -MAFG

p= 0.1047; WM793-GFP vs -MAFG p=0.9196. Error bars represent mean + s.d. (**g**) Volcano plot of differentially expressed genes identified by RNA sequencing comparing GFP and MAFG overexpression in A375. ns, not significant; ** $p < 0.01$; *** $p < 0.001$.

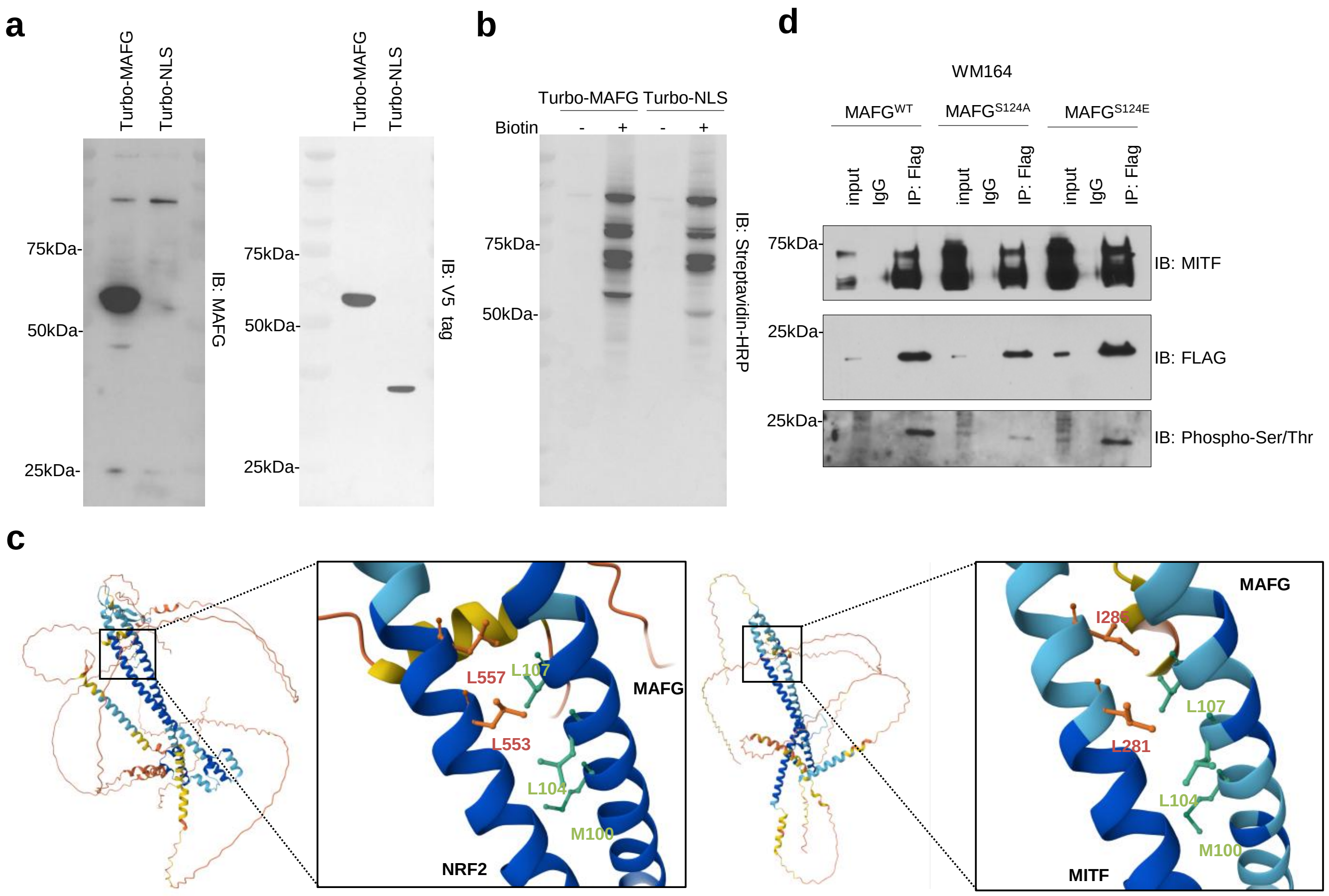

Supplementary Figure 6: MAFG interacts with MITF. (**a**) Western blot showing the expression of the TurboID-MAFG fusion construct in WM164 cells blotted with MAFG (left) or V5 tag (right). Blot shown represents one out of three experiments with similar results. (**b**) Biotin proximity labeling using N-terminal TurboID-MAFG fusion construct in WM164 cells blotted with Streptavidin-HRP. Blot shown represents one out of three experiments with similar results. (**c**) AlphaFold3 prediction of the interaction between MAFG and NRF2 (left) and between MAFG and MITF (right). The MAFG-MITF interaction at the leucine zipper interface mimics the interaction of the MAFG-NRF2 dimer. (**d**) Co-immunoprecipitation of MAFG and MITF in WM164 cells overexpressing $MAFG^{WT}$, $MAFG^{S124A}$, or $MAFG^{S124E}$. Blot shown represents one out of three experiments with similar results.

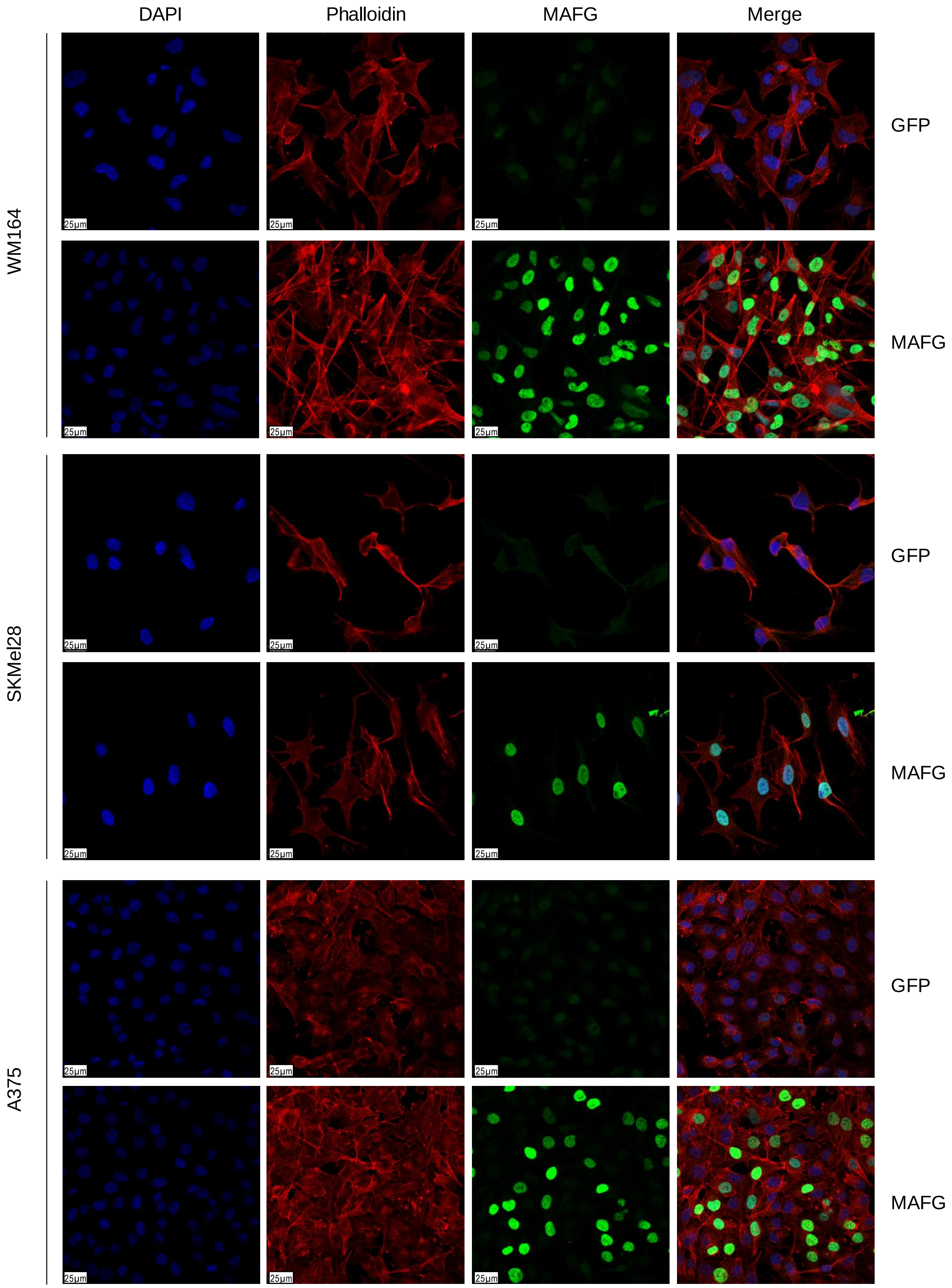

Supplementary Figure 7: Expression of MAFG in human melanoma cell lines. Immunofluorescence for MAFG in WM164, SKMel28 and A375 cells overexpressing GFP or MAFG. Scale bars indicate 25µm.

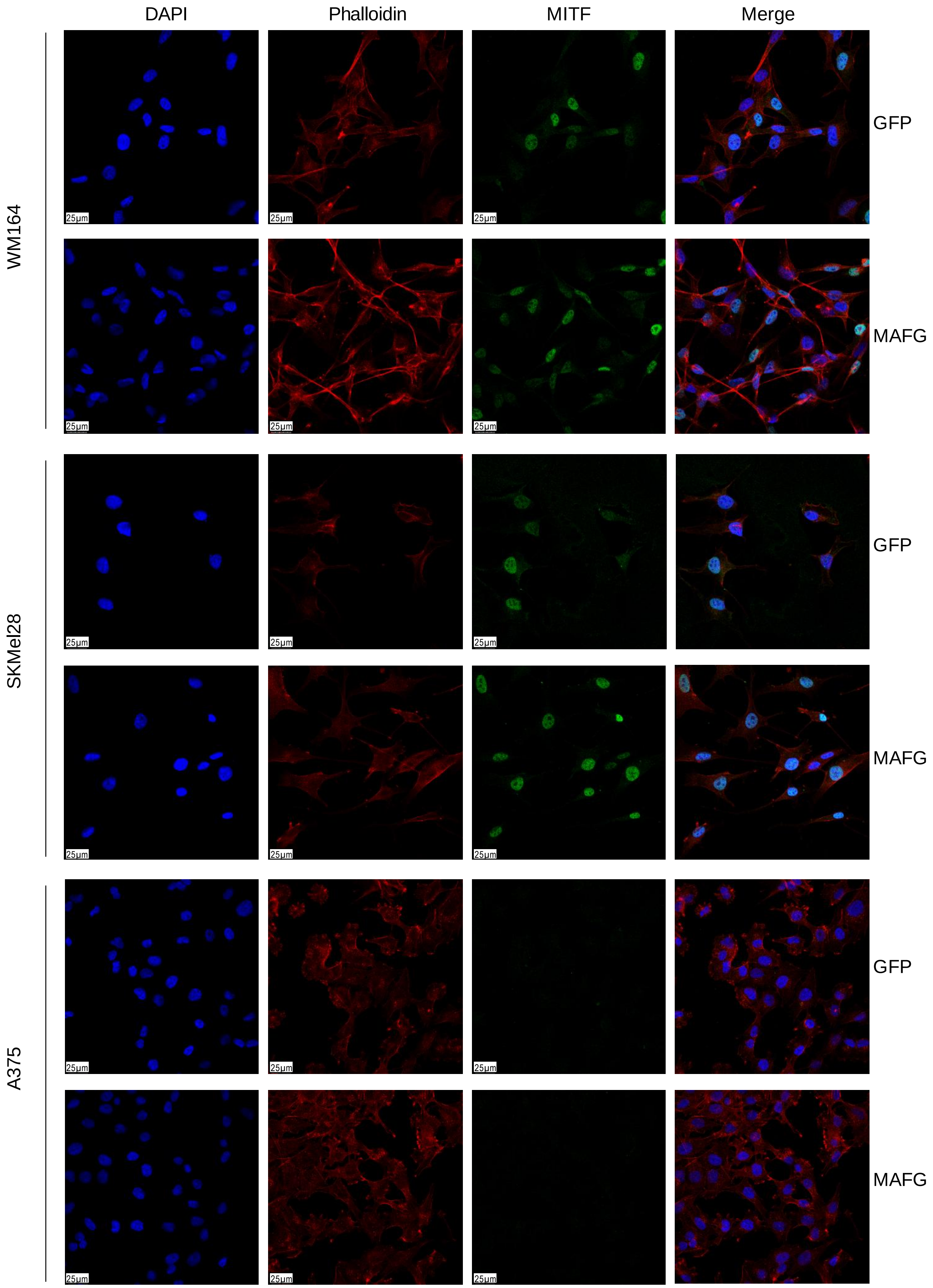

Supplementary Figure 8: Expression of MITF in human melanoma cell lines. Immunofluorescence for MITF in WM164, SKMel28 and A375 cells overexpressing GFP or MAFG. Scale bars indicate 25µm.

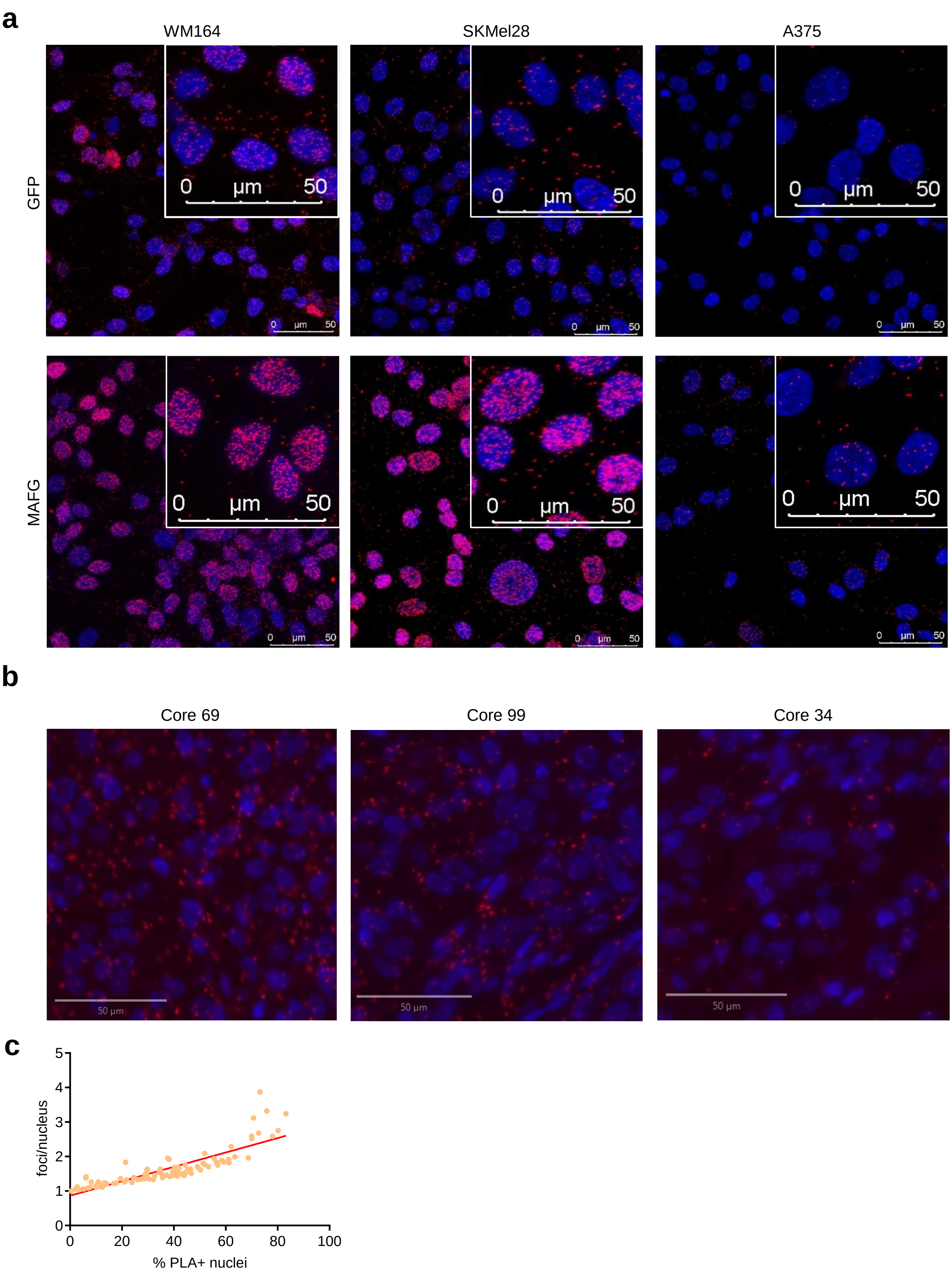

Supplementary Figure 9: MAFG interacts with MITF. (**a**) Images of proximity ligation assays (PLA) in WM164, SKMel28 and A375 cells overexpressing either GFP or MAFG. (**b**) Representative images of PLA performed on a melanoma metastasis TMA. (**c**) Correlation between the percent of PLA positive nuclei per core and the average number of PLA foci per nucleus in a melanoma metastasis TMA.

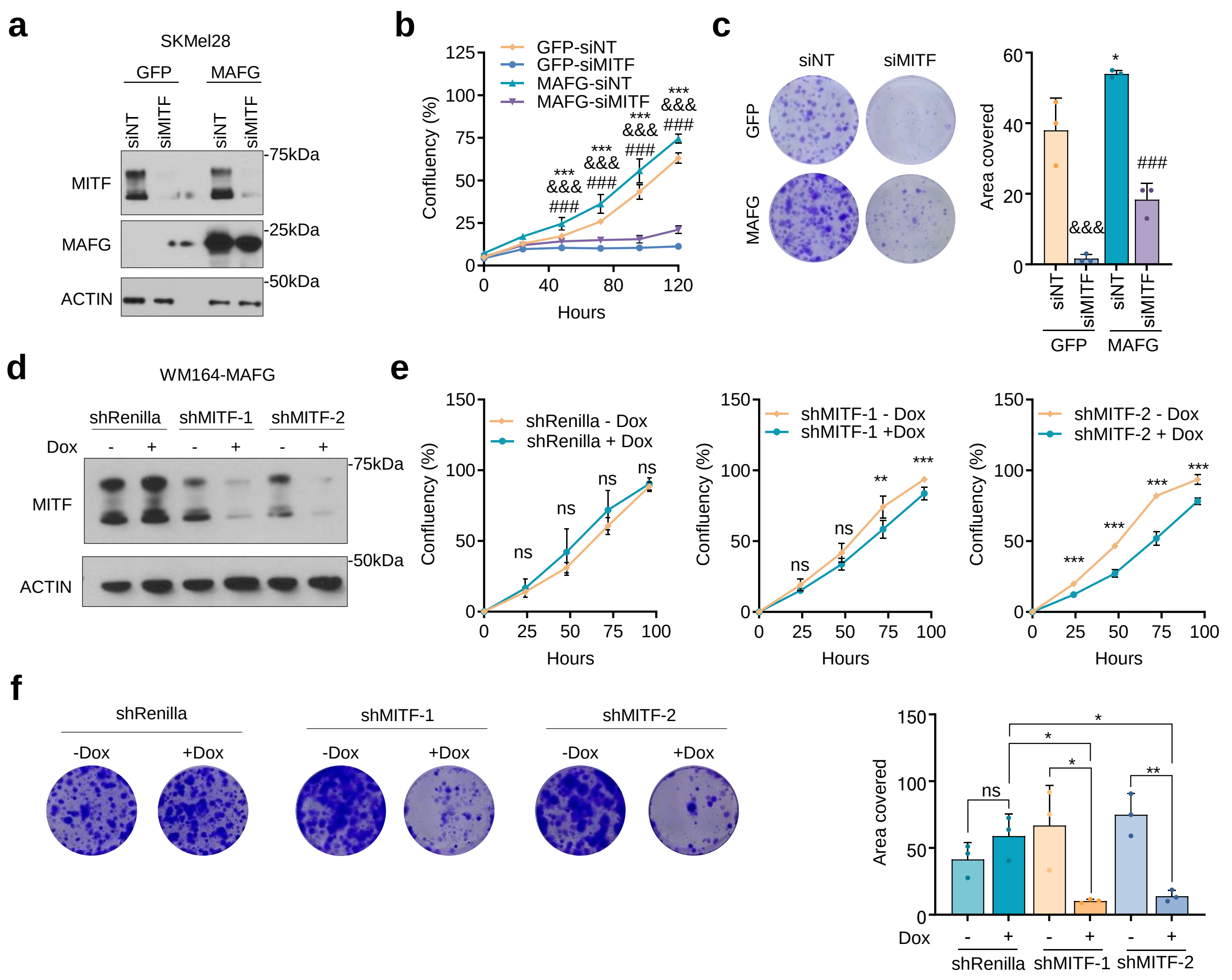

Supplementary Figure 10: MITF is required for the MAFG oncogenic effects. (**a**) Western blot validating the silencing of MITF in SKMel28 cells overexpressing MAFG or GFP. Blot shown represents one out of three experiments with similar results. (**b,c**) Proliferation (b) and focus formation (c) assays in SKMel28 cells overexpressing MAFG or GFP following MITF silencing. For proliferation assay, n=5 technical replicates from one out of two biological replicates are represented. Statistical significance was determined using Welch's two-tailed t test for each time point. p-value of each comparison at 120h is as follows: GFP-siNT vs MAFG-siNT p=0.0002; GFP-siNT vs GFP-siMITF, p<0.0001; MAFG siNT vs MAFG siMITF, p<0.0001; GFP-siMITF vs MAFG-siMITF, p= 0.0002. Error bars represent mean ± s.d. For focus formation assay, n=3 technical replicates from one out of two biological replicates are represented. Statistical significance was determined by one-way ANOVA with Tukey's multiple-comparison test. GFP-siNT vs MAFG-siNT (p= 0.0225); GFP-siNT vs GFP-siMITF (p=0.0001), MAFG siNT vs MAFG siMITF (p= 0.0001); GFP-siMITF vs MAFG siMITF (p= 0.0182). Error bars represent mean + s.d. *p<0.05; # p<0.05; ## p<0.01; ns, not significant; **p<0.01; ***p<0.001; ****p< 0.0001. GFP-siNT vs. MAFG-siNT: *p<0.01; ***p<0.0001. GFP-siNT vs GFP-siMITF: &&& p<0.001. MAFG-siNT vs MAFG-siMITF: ###, p<0.001. (**d**) Western blot validating the silencing of MITF with two doxycycline-inducible shRNAs in WM164 cells overexpressing MAFG. Blot shown represents one out of three experiments with similar results (**e,f**) Proliferation (e) and focus formation (f) assays showing the effect of MITF silencing with two doxycycline-inducible shRNAs in WM164 cells overexpressing MAFG. For proliferation assay, n=5 technical replicates from one out of two biological replicates are represented. Statistical significance was determined using Welch's two-tailed t-test for each time point. p-value of each comparison at 96h is as follows: shRenilla – Dox vs shRenilla +Dox, p=0.4643; shMITF-1 – Dox vs shMITF-1 +Dox, p=0.0055; shMITF-2 – Dox vs shMITF-2 +Dox, p=0.0001. Error bars represent mean ± s.d. For focus formation assay, n = 3 technical replicates from one out of two biological replicates are represented. Statistical significance was determined by one-way ANOVA with Tukey's multiple-comparison test. p-values for each comparison are as follows: shRenilla – Dox vs shRenilla + Dox, p=0.781; shMITF-1 -Dox vs shMITF-1 +Dox,

p=0.0316; shMITF-2 -Dox vs shMITF-2 +Dox, p=0.0067; shRenilla +Dox vs shMITF-1 +Dox, p=0.0116; shRenilla + Dox vs shMITF-2 +Dox, p=0.0500. Error bars represent mean + s.d. ns, not significant; *p<0.05; **p<0.01; ***p<0.001.

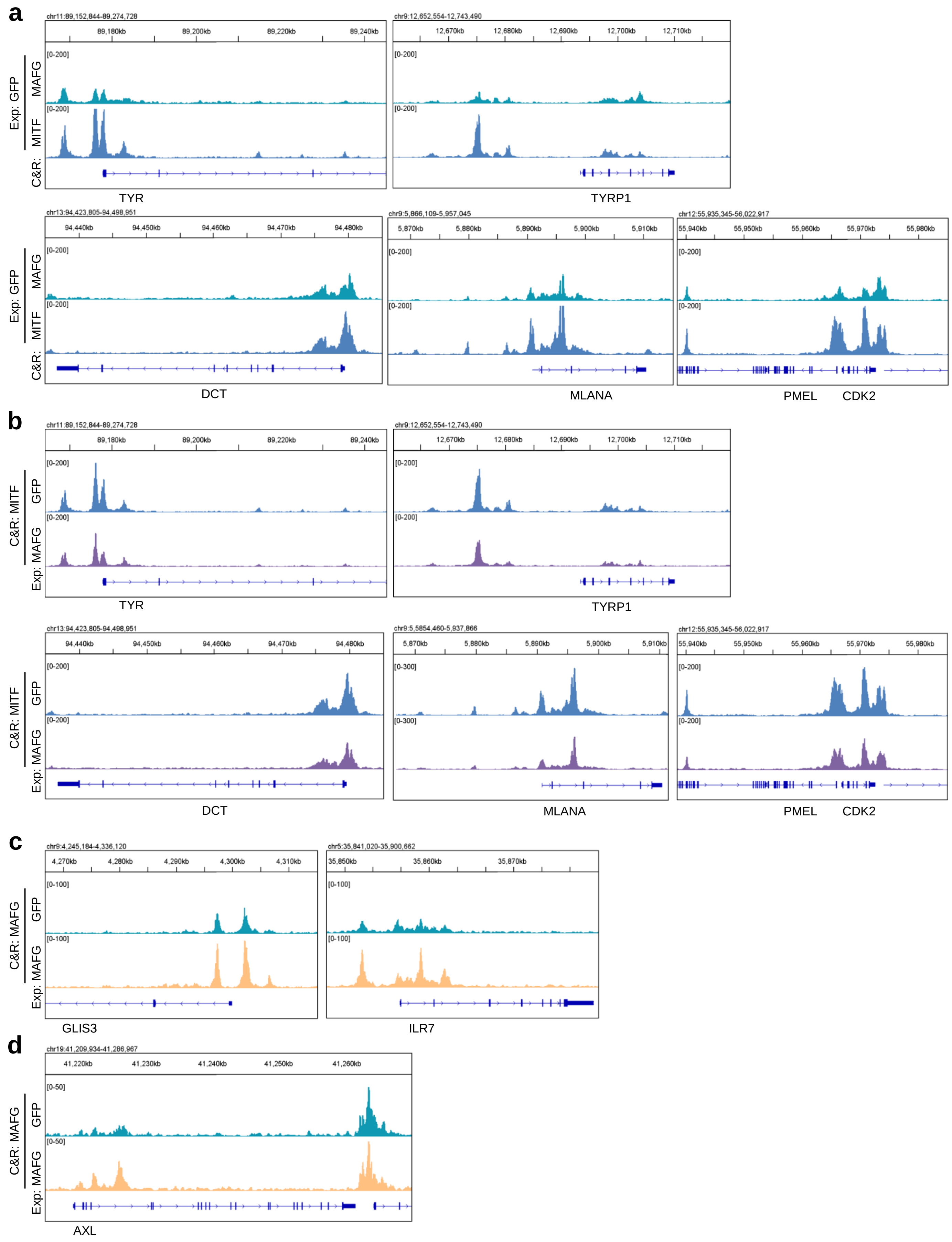

Supplementary Figure 11: MAFG impacts MITF target gene binding and transactivation. (**a**) IGV plots for MAFG and MITF in WM164 control cells showing the MITF target genes *TYR*, *TYRP1*, *DCT*, *MLANA*, and *PMEL*/*CDK2*. (**b**) IGV plots for MITF in WM164 cells expressing GFP or MAFG showing the MITF target genes *TYR*, *TYRP1*, *DCT*, *MLANA*, and *PMEL*/*CDK2*. (**c,d**) IGV plots for MAFG in WM164 cells expressing

GFP or MAFG cells showing *GLIS3* and *ILR7* (c) as well as *AXL* (d). Exp: overexpression of GFP or MAFG; C&R: CUT&RUN of MAFG or MITF.

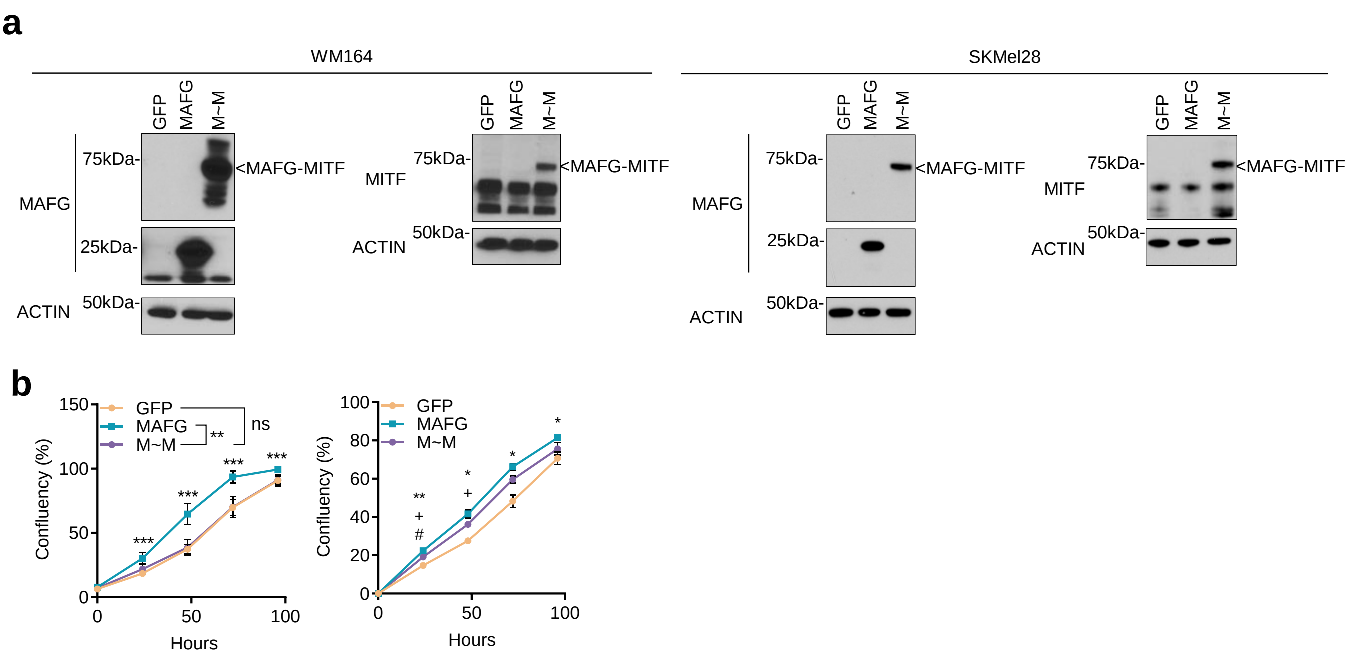

Supplementary Figure 12: Expression of a tethered MAFG~MITF dimer. (**a**) Western blot showing the expression of the MAFG~MITF tethered dimer construct in WM164 and SKMel28 cells. Blot shown represents one out of three experiments with similar results. (**b**) Proliferation assays in WM164 (left) and SKMel28 (right) cells overexpressing the MAFG~MITF dimer (M~M) or a GFP control. For proliferation assay, n=5 technical replicates from one out of two biological replicates are represented. Statistical significance was determined using Welch's two-tailed t-test for each time point. p-value of each comparison at 96h is as follows: WM164-GFP vs -MAFG p= 0.0046; WM164-GFP vs -M~M, p=0.8416; WM164-MAFG vs -M~M p= 0.0014; SKMel28-GFP vs -MAFG p= 0.0387; SKMel28-GFP vs -M~M, p=0.0957; SKMel28-MAFG vs -M~M p= 0.3099. Error bars represent mean ± s.d.. Ns, not significant; *** $p < 0.001$. GFP vs M~M: ++ $p < 0.01$; +++ $p < 0.001$. MAFG vs M~M: ## $p < 0.01$; ### $p < 0.001$.

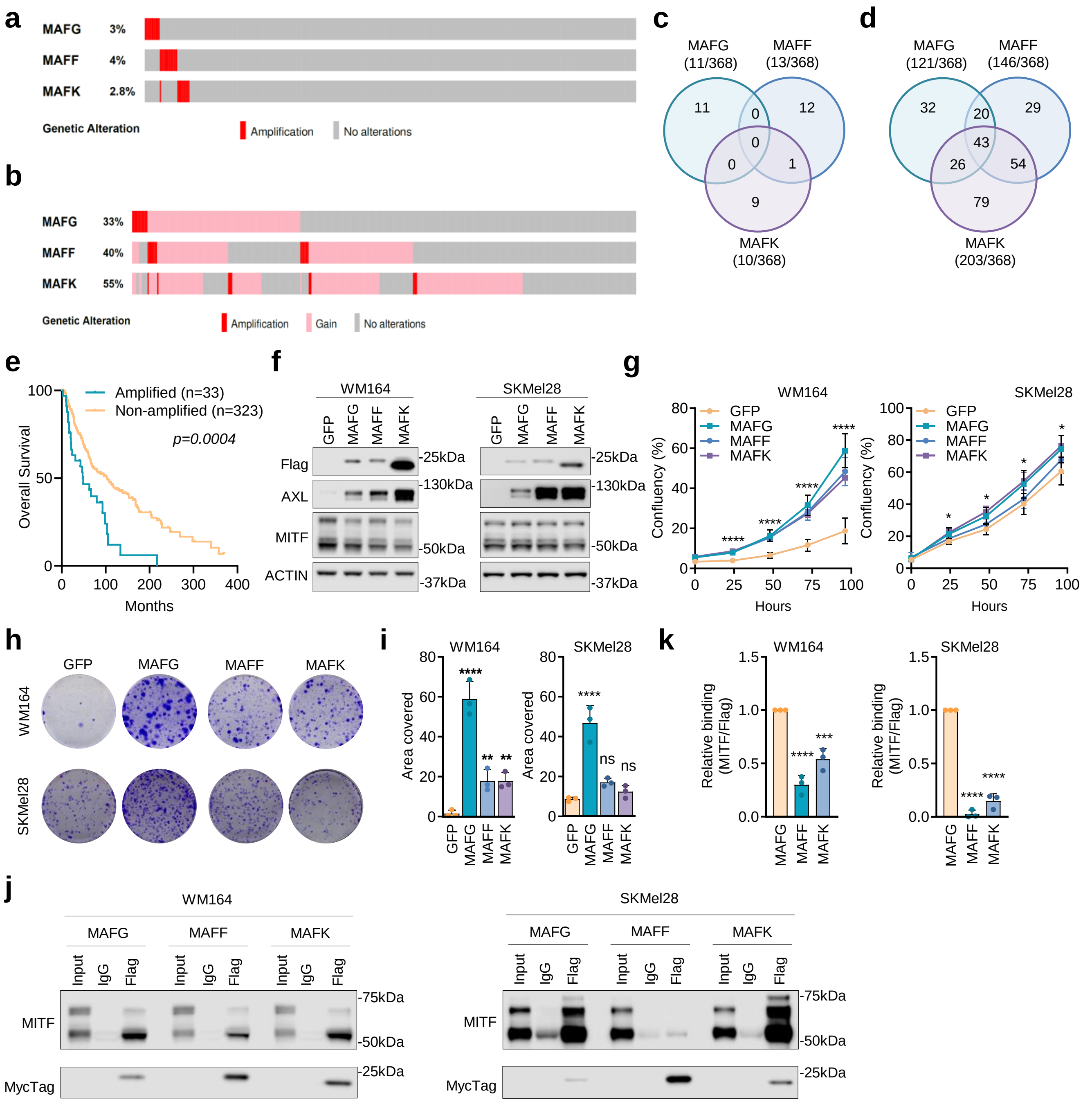

Supplementary Figure 13: MAFF and MAFK possess oncogenic potential in melanoma. (**a,b**) Oncoprint showing the co-occurrence of *MAFG*, *MAFF*, and *MAFK* amplifications (a) or copy number gains and amplifications (b) in samples from The Cancer Genome Atlas skin cutaneous melanoma (TCGA-SKCM) dataset. (**c,d**) Venn diagrams showing the co-occurrence of *MAFG*, *MAFF*, and *MAFK* amplifications (c) or copy number gains and amplifications (d) in the SKCM-TCGA cohort. (**e**) Survival analysis from TCGA (PanCancer Atlas, n = 473) comparing melanoma patients with or without amplification of at least one of the sMAF genes using the Log-rank (Mantel-Cox) test (p=0.0004). (**f**) Western blot showing the overexpression of Flag-MAFG, Flag-MAFK and Flag-MAFF and their effect on AXL and MITF levels in WM164 and SKMel28 cells. Blot shown represents one out of three experiments with similar results. (**g**) Proliferation assays of WM164 and SKMel28 cells overexpressing MAFG, MAFF, MAFK, or GFP. For proliferation assay, n=6 technical replicates from one out of two biological replicates are represented. Statistical significance was determined using Welch's two-tailed t-test for each time point. p-value of each comparison at 96h is as follows: WM164-GFP vs –MAFG, p<0.00001; WM164-GFP vs -MAFF, p<0.00001; WM164-GFP vs –MAFK, p<0.00001; SKMel28-GFP vs -MAFG p= 0.0197; SKMel28-GFP vs -MAFF, p=0.1778; SKMel28-MAFG vs –MAFK, p= 0.0056. Error bars represent mean ± s.d. (**h,i**) Focus formation assays of WM164 and SKMel28 cells overexpressing MAFG, MAFF, MAFK, or GFP. n=3 technical replicates from one out of three biological

replicates are represented. . Statistical significance was determined by one-way ANOVA with Tukey's multiple-comparison test. p-values for each comparison are as follows: WM164-GFP vs –MAFG, p=0.0001; WM164-GFP vs -MAFF, p=0.0.0323; WM164-GFP vs –MAFK, p=0.0329; SKMel28-GFP vs -MAFG p=0.0001; SKMel28-GFP vs -MAFF, p=0.2156; SKMel28-MAFG vs –MAFK, p= 0.7738. Error bars represent mean + s.d. (**j,k**) Co-immunoprecipitation of MITF in WM164 and SKMel28 cells overexpressing MAFG, MAFF, or MAFK. Quantification (k) of the relative binding of MAFG, MAFF, and MAFK to MITF is shown on the right. sMAF proteins were immunoprecipitated with a Flag tag antibody and detected with a Myc tag antibody. Co-immunoprecipitated MITF was normalized to sMAF-Myc signals. Blot shown represents one out of three experiments with similar results. n=3 technical replicates from one out of two biological replicates are represented. . Statistical significance was determined by one-way ANOVA with Tukey's multiple-comparison test. p-values for each comparison are as follows: WM164 MAFG vs MAFF, p=0.0001; WM164 MAFG vs MAFK, p=0.0008; SKMel28 MAFG vs MAFF, p<0.00001; SKMel28 MAFG vs MAFK, p<0.00001. Error bars represent mean + s.d.. ns, not significant; ** p < 0.01; *** p < 0.001; **** p < 0.0001.

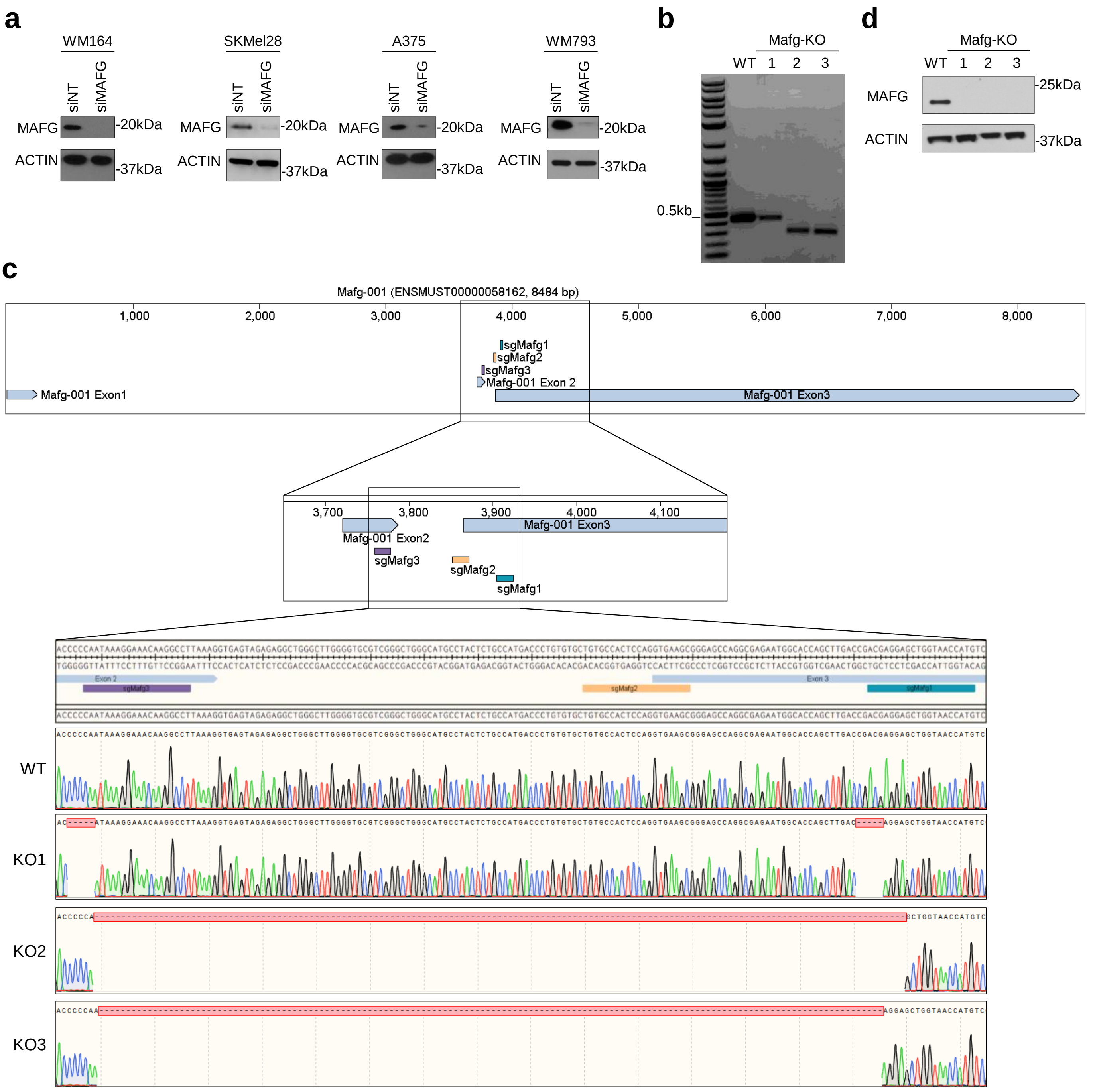

Supplementary Figure 14: MAFG is a vulnerability in melanoma. (**a**) Western blot validating the silencing of MAFG in WM164, SKMel28, A375 and WM793 cells using an ON-TARGETplus MAFG siRNA pool that contains 4 siRNAs against MAFG. Blot shown represents one out of three experiments with similar results. (**b**) Schematic representation of the location of the guide RNAs used to knock-out Mafg and histogram of one wildtype and three Mafg knockout BPP ES cell clones. Colored boxes in the histograms represent the sgRNA binding sites. (**c**) PCR on genomic DNA of the wildtype and three Mafg knockout BPP ES cell clones from (b). (**d**) Western blot of the one wildtype and three knockout BPP ES cell clones from (b). Blot shown represents one out of three experiments with similar results.

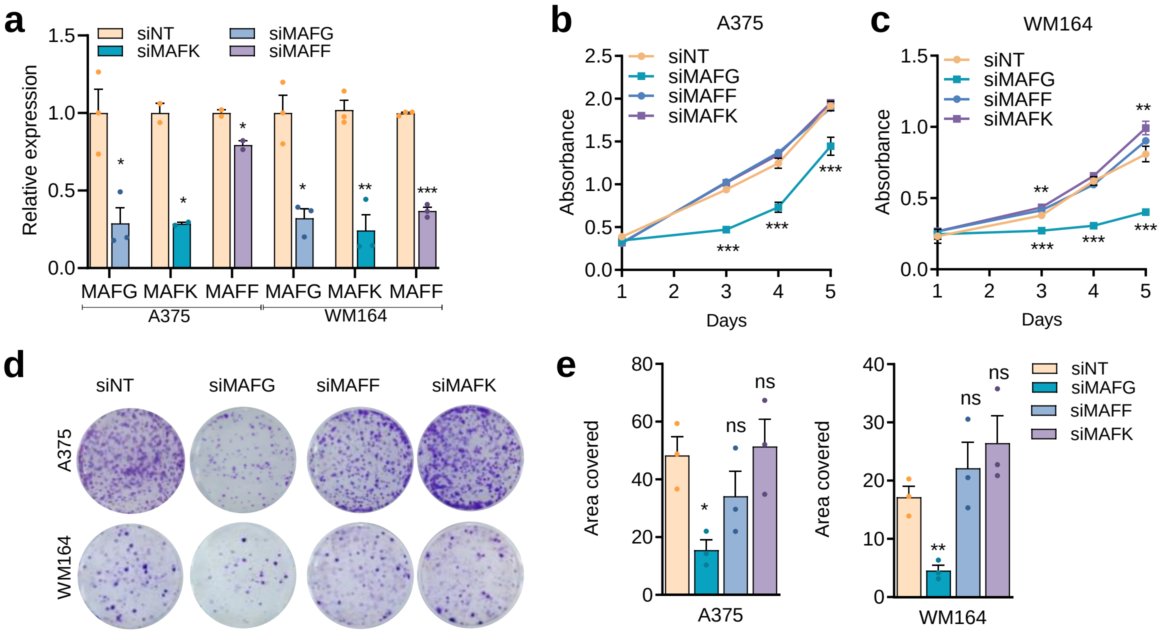

Supplementary Figure 15: MAFF and MAFK are dispensable for melanoma cell proliferation. (**a**) qRT-PCR validating silencing of *MAFG*, *MAFF*, or *MAFK* in A375 and WM164 cells. n = 2 technical replicates from one out of two biological replicates are represented. Statistical significance was determined using Welch's two-tailed t-test. p-value of each comparison is as follows: A375-siNT vs –siMAFG, p= 0.0231; A375-siNT vs –siMAFK, p= 0.0488; A375-siNT vs –siMAFF, p= 0.0340; WM164-siNT vs –siMAFG, p= 0.0132; WM164-siNT vs –siMAFK, p= 0.0051; WM164-siNT vs –siMAFF, p=0.0006. Error bars represent mean + s.d. (**b,c**) Proliferation assays in A375 (b) and WM164 (c) cells upon silencing of MAFG, MAFF, or MAFK, n = 5 technical replicates from one out of two biological replicates are represented. Statistical significance was determined using Welch's two-tailed t-test for each time point. p-value of each comparison at 5 days is as follows: A375-siNT vs –siMAFG, p= 0.0001; A375-siNT vs –siMAFK, p= 0.3845; A375-siNT vs –siMAFF, p= 0.2787; WM164-siNT vs –siMAFG, p<0.00001; WM164-siNT vs –siMAFK, p= 0.0172; WM164-siNT vs –siMAFF, p=0.0005. Error bars represent mean ± s.d. (**d,e**) Focus formation assays in A375 and WM164 cells upon silencing of MAFG, MAFF, or MAFK. Representative images (d) and quantification (e) are shown. n=3 technical replicates from one out of two biological replicates are represented. Statistical significance was determined using Welch's two-tailed t-test. p-value of each comparison is as follows: A375-siNT vs –siMAFG, p= 0.0212; A375-siNT vs –siMAFK, p= 0.2683; A375-siNT vs –siMAFF, p= 0.7991; WM164-siNT vs –siMAFG, p=0.0089; WM164-siNT vs –siMAFK, p= 0.3872; WM164-siNT vs –siMAFF, p=0.1765. Error bars represent mean + s.d. ns, not significant; * p < 0.05; ** p < 0.01; *** p < 0.001.

a

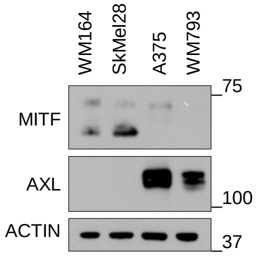

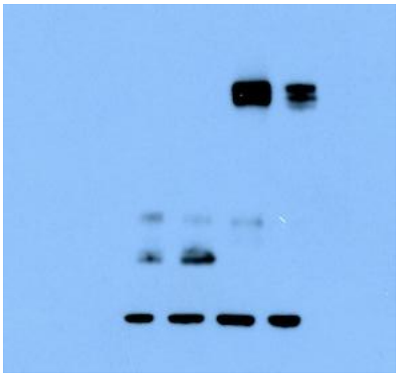

d

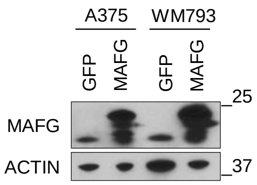

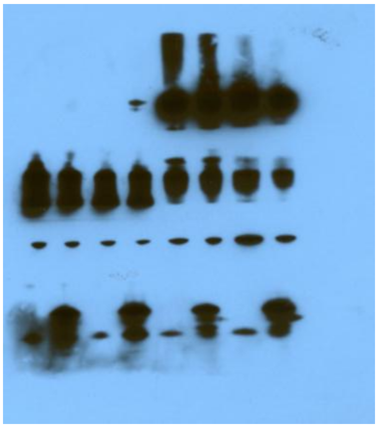

Supplementary Figure 5

d

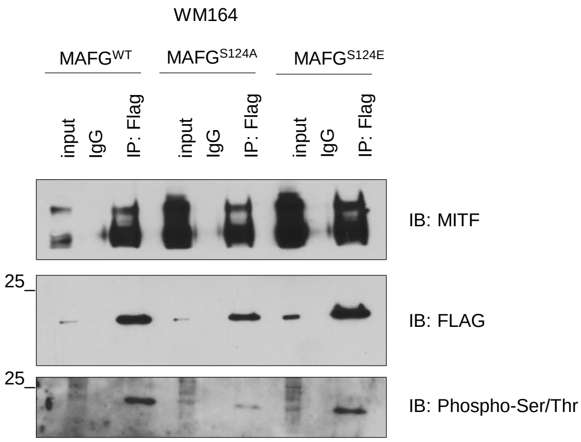

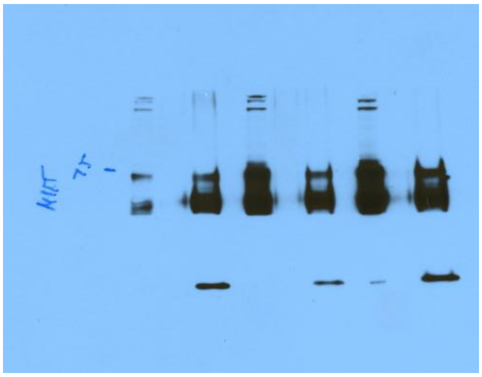

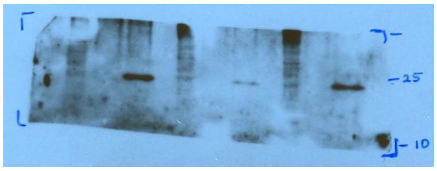

Supplementary Figure 6

a

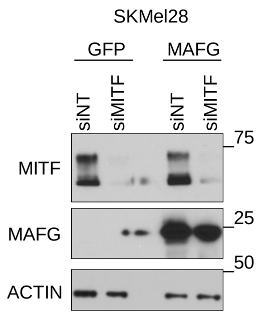

d

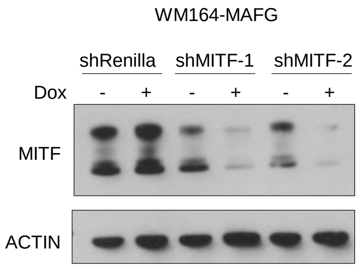

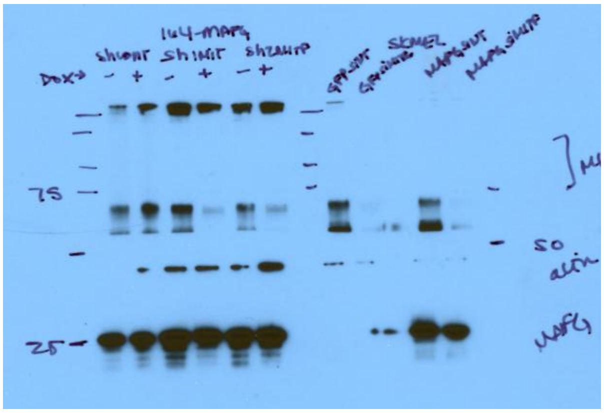

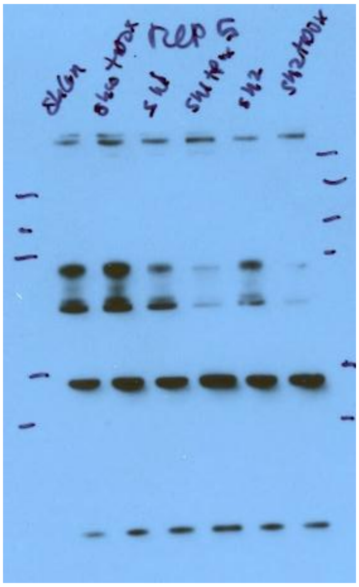

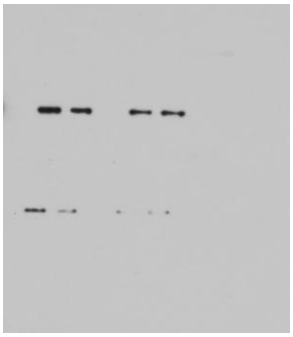

Supplementary Figure 10

a

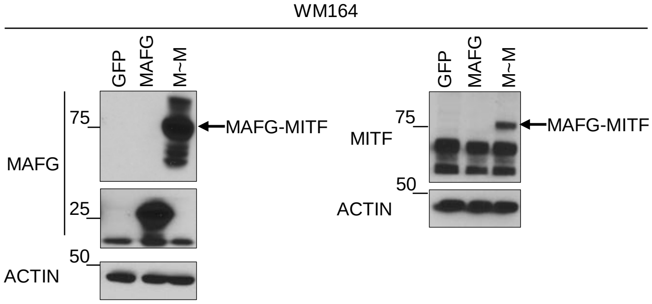

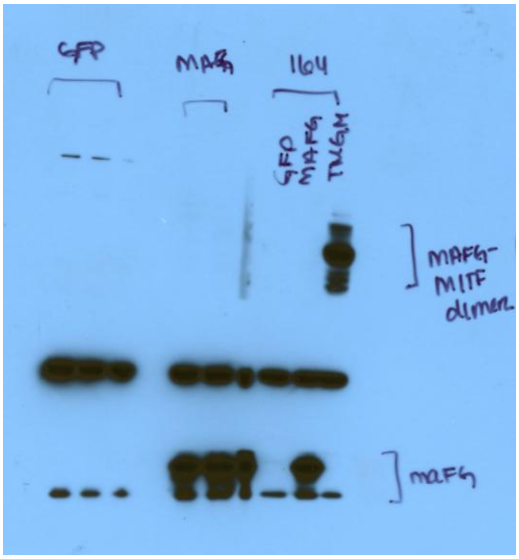

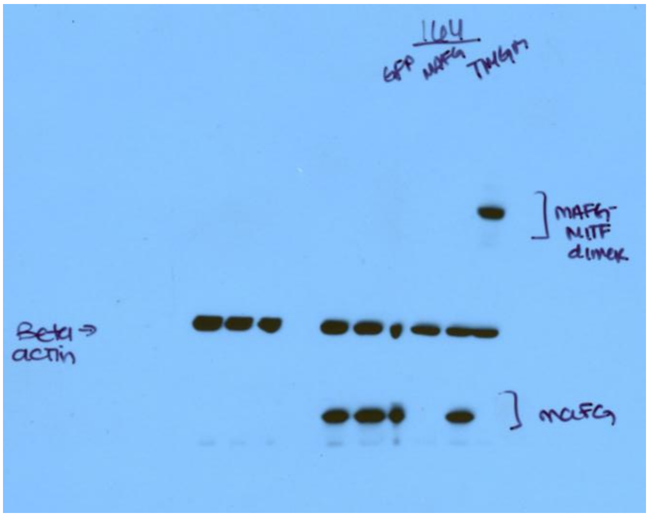

Supplementary Figure 12

a

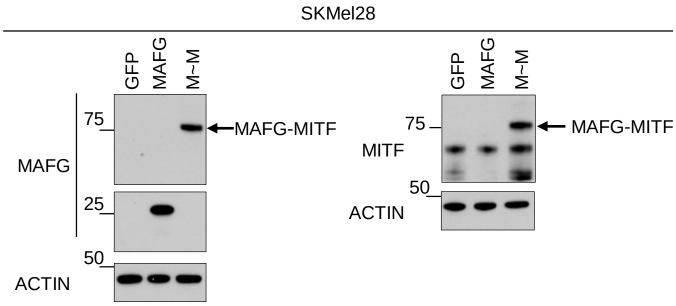

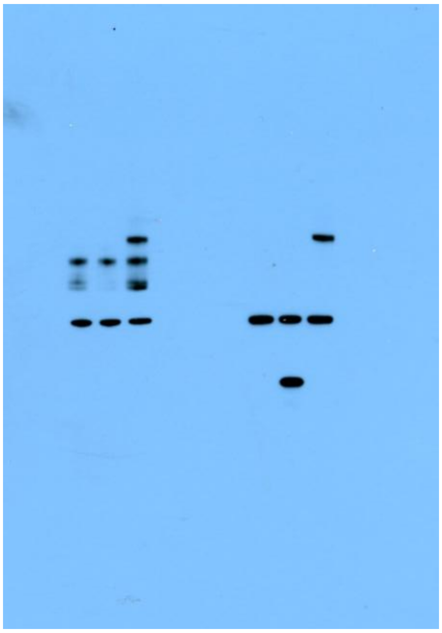

Supplementary Figure 12

f

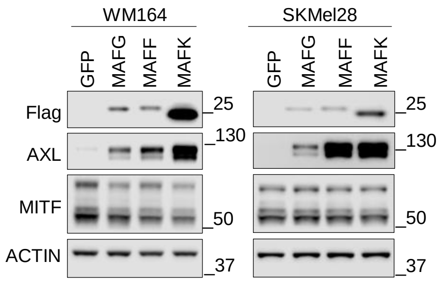

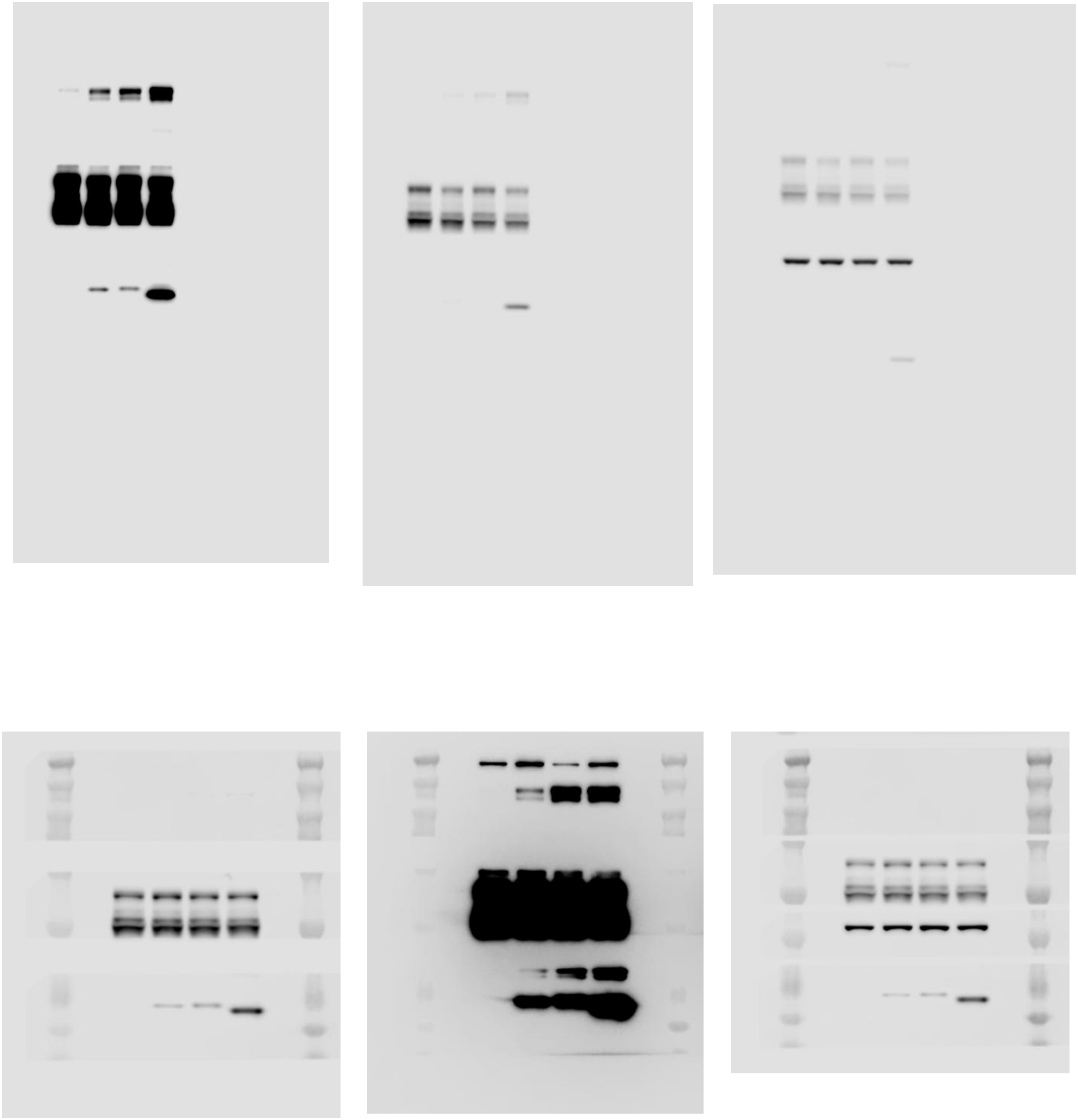

Supplementary Figure 13

j

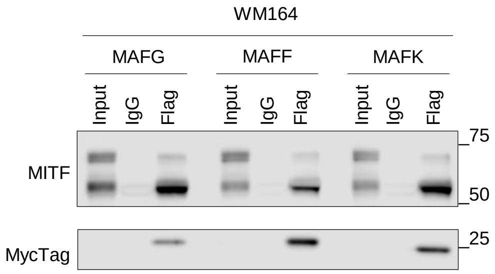

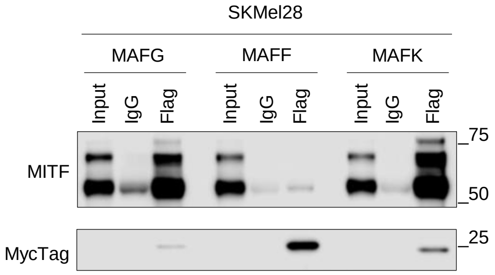

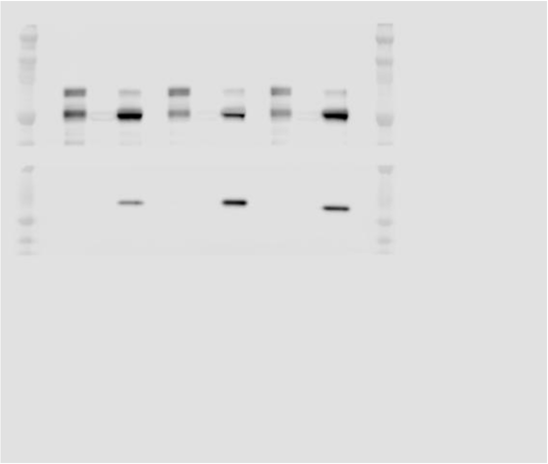

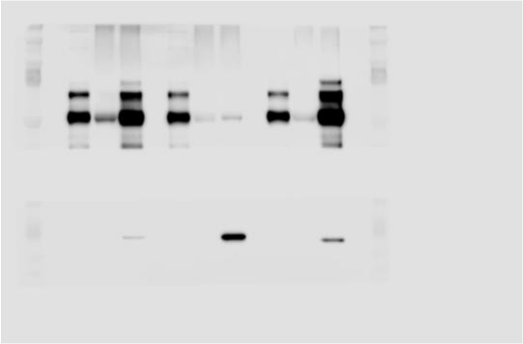

Supplementary Figure 13

a

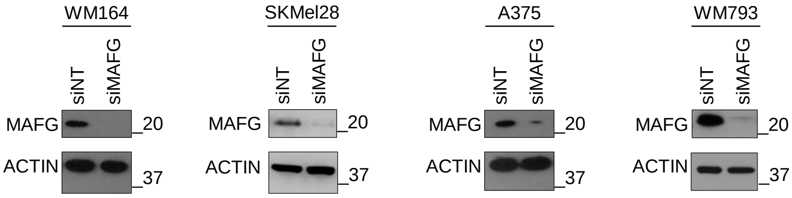

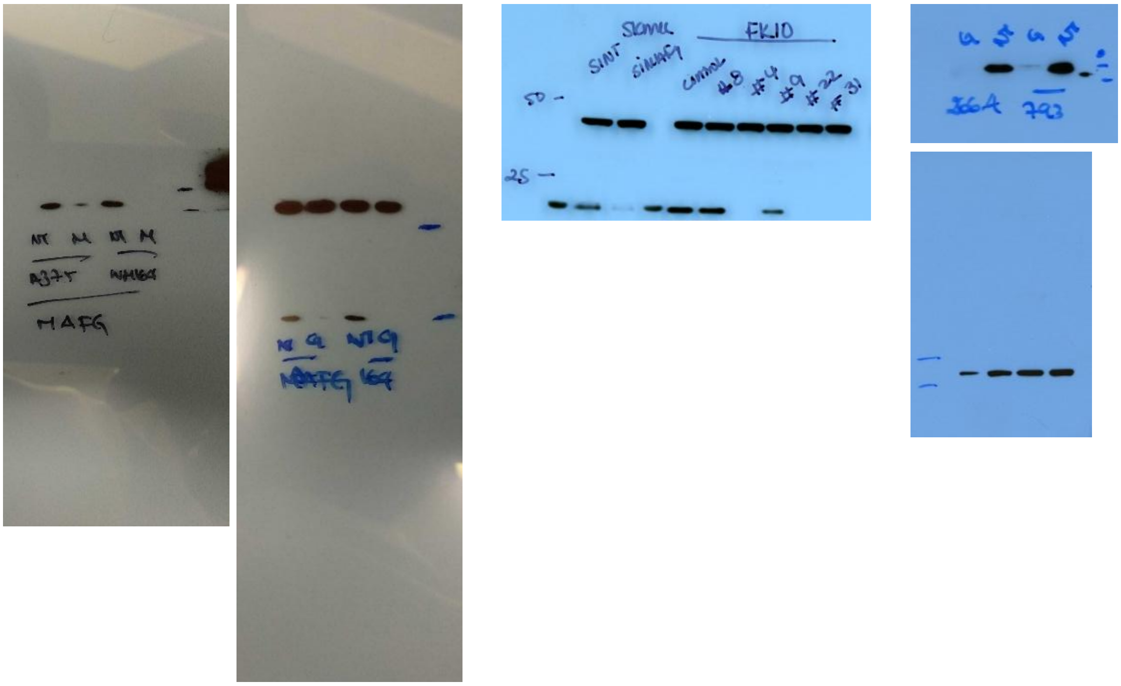

Supplementary Figure 14

c

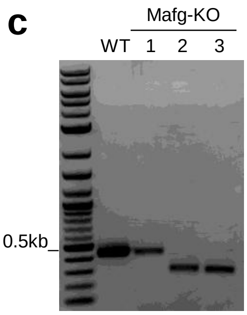

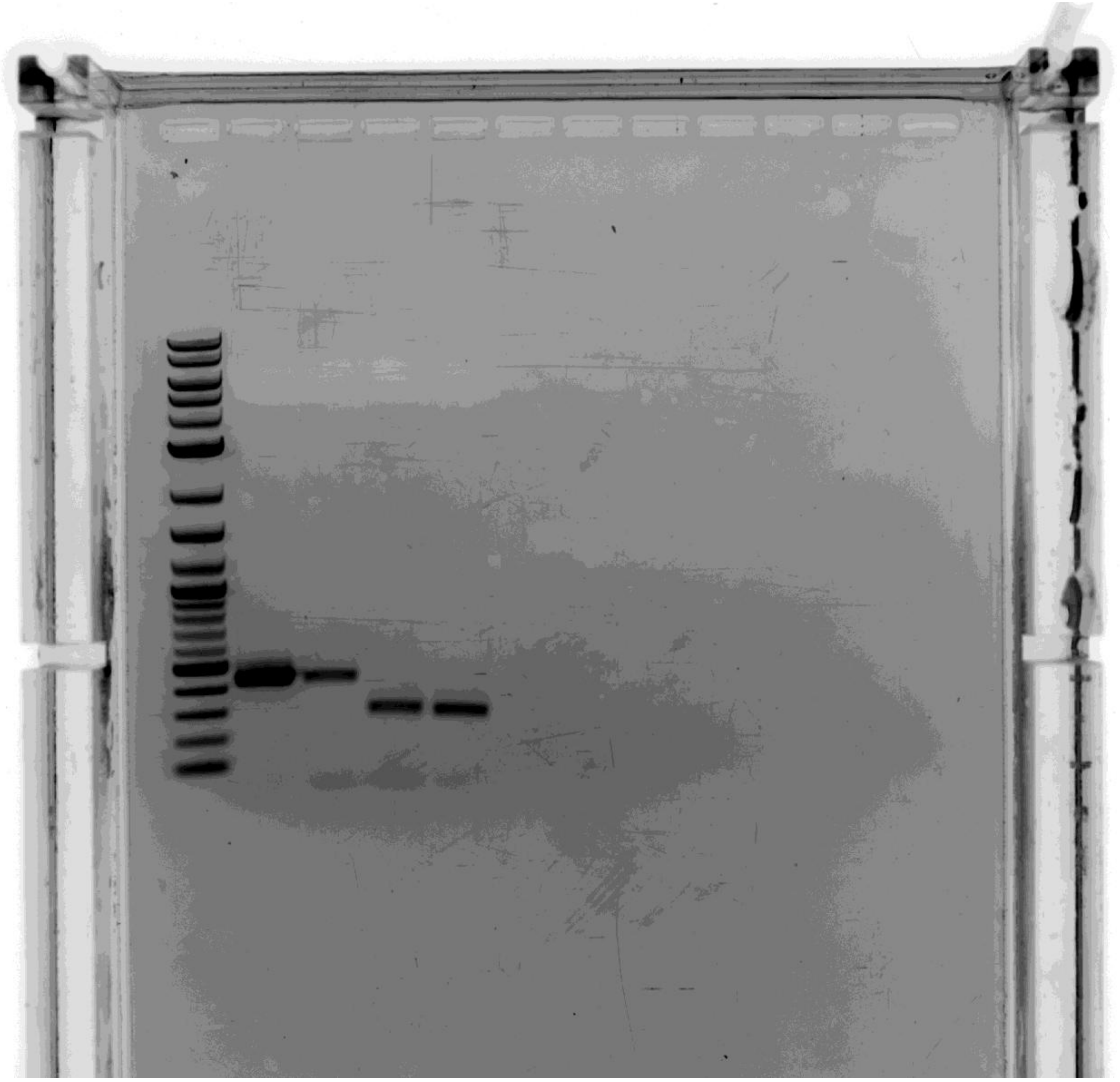

Supplementary Figure 14

d

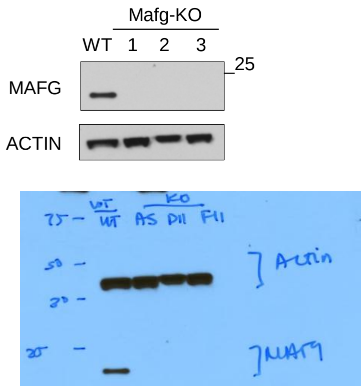

Supplement: Supplementary file 1 — Supplementary Information [file 41467_2026_73291_MOESM1_ESM.pdf]
