# Supplementary material for: A MAFG~MITF complex drives melanoma phenotype switching and progression
Source: Nat Commun. 2026 May 21;17:6685. doi: 10.1038/s41467-026-73291-x (PMC13385793; doi:10.1038/s41467-026-73291-x)
Supplement: Supplementary file 2 — Description of Additional Supplementary Files [file 41467_2026_73291_MOESM2_ESM.pdf]

Description of Additional Supplementary Files

Supplementary Dataset 1: TurboID dataset identifying proteins in proximity with MAFG in WM164 cells.

Supplementary Dataset 2: List of Cut&Run peaks identified for MAFG and MITF in WM164 cells expressing either GFP or MAFG.

Supplementary Dataset 3: Differential binding of MAFG and MITF at unique and shared genomic sites based in Cut&Run performed in WM164 cells expressing either GFP or MAFG.

Supplementary Dataset 4: Differentially expressed genes harboring MAFG and/or MITF peaks that significantly change upon MAFG overexpression.
