# Supplementary material for: A MAFG~MITF complex drives melanoma phenotype switching and progression
Source: Nat Commun. 2026 May 21;17:6685. doi: 10.1038/s41467-026-73291-x (PMC13385793; doi:10.1038/s41467-026-73291-x)
Supplement: Supplementary file 8 — Transparent Peer Review file [file 41467_2026_73291_MOESM8_ESM.pdf]

nature portfolio

Peer Review File

A MAFG~MITF complex drives melanoma phenotype switching and progression

Corresponding Author: Dr Florian Karreth

This file contains all reviewer reports in order by version, followed by all author rebuttals in order by version.

Version 0:

Reviewer comments:

Reviewer #1

(Remarks to the Author)
In this manuscript Olga Vera et. al. study the role of the transcription factor MAFG in the control of MITF along its renowned role in phenotype switching, which has been implicated in melanoma progression. Authors suggest that the complex formed between MAFG and MITF occupies >50% of MITF targets in the genome, which attenuates MITF ability to activate these genes transcription. Manipulation of MAFG revealed that it is sufficient to drive a dediffernetiated cell state and accelerate melanoma progression, which is MITF dependent. Authors further show that MAFG is important for melanoma proliferation and progression from nevi to melanoma state in genetic models. In as much authors uncover an important regulator of MITF function along melanoma development and progression. In patients, MAFG expression is elevated and associated with worse survival in melanoma, confirming its relevance for human melanoma. While of interest, the presented manuscript appears to be premature, as it lacks important controls, number of claims are not supported by data shown, and quality of data, at times, is subpar.

General points
why are the WM164 cells less sensitive to MITF silencing compared to SKMel28? Authors claim reduced proliferation and foci formation, but relative to SKMel28, the reduction is not as clear.

Considering MAFG is phosphorylated by ERK for stability, does this phosphorylation promotes the dimerization of this protein with MIFT? Is phosphorylation necessary for dimerization to take place? Is the overexpressed MAFG phosphorylated (stable) or the effect is due to abundance?
MITF has multiple isoforms (also seen in the data presented). Does MAFG interact with any specific isoforms of MITF?
Multiple cell lines are used throughout a project but for certain assays only one is used. It is not clear why certain assays are done with one cell line rather than another? (for example, the CUT&RUN assay was only used in WM164, or in assessing other components (MAFF and MAFK) only one cell line is assessed) Why was WM164 selected?

Specific points
Figure 1a. How is the expression of MAFG different in transformed tissues, compared to normal skin? Data set GSE3189 provides 7 samples of normal skin. It would be interesting to demonstrate the relative amounts.
Figure 1b. please add representative images.
Figure S1b. How large is the shift in Ectopic MAFG? Please label the membranes with MW markers. How is the basal level of MAFG protein different among these cell lines? Are the exposures displayed in images comparable? It seems like the OE of MAFG had different efficiencies in different cell lines. How did the authors account for that in the follow-up experiment?
Figure 1d. First panel - isn't the y-axis supposed to indicate confluency (%) rather than absorbance?
Figure 2b. The figure presented does not represent "accelerated melanoma development", rather it's another way to shows survival, which is not much different from data shown in Figure 2c. It would be interesting to know if there are differences in tumor sizes.
Figure 2g. Please replace MAFG-GFP panel. Image seems to be out of focus or has been filtered. Also, according to the manuscript, Figure 2g is supposed to show "nodular regions" hence, "increased cellularity". The data presented does not match, since the data shows quantification of overall MAFG positive cells by IHC.
Figure 3b. Decrease in MITF is not noticeable specially in SKMel28 cells. Also, please add the WB, confirming overexpression of MAFG in these cell lines.
Figure 3h and i. In the legend, it is mentioned "MAFG expression (left) in human/mouse melanoma cell clusters represent

different phenotypic states (right)", however the panel is in the opposite order.
Figure S4d. Please label WB (MITF antibody recognizes different isoforms-which would be something to attend to in assessing MAFG-MITF interactions.
Figure S4g. Figure legend does not match the panel. Data suggested in the legend are the controls needed for panel 3b, which are not provided.
Figure 4b. The input for the MITF pulldown in SkMel28 cells is not clear. Please provide a more exposed membrane.
Figure S5c and 4d. The number of PLA foci in the 3 cell lines are significantly different. How is this biologically relevant between these 3 cell lines? Or perhaps is this difference due to transfection efficiency of MAFG. From previous controls, it appears that not all cells overexpress MAFG with the same efficiency (i.e. S1b, S4g). As a control, authors could include an IF of the overall levels of MAFG, MITF and GFP in these cell lines in addition to the PLA images to see if the basal or overexpressed levels of these proteins is comparable prior to PLA assay. Also, DAPI staining is overexposed in all images (except WM164-MAFG). Please replace with less exposed images. Does overexpression of MAFG effect the localization of this protein? There are changes in the cytosolic pool of these proteins, that could be interesting to follow.
Figure 4f. Please include the control blot for MAFG confirming overexpression of the protein.
Figure 6a. colony formation images do not match the data. Images show significantly higher # of colonies in WM164 cells compared to SkMel28, however, total area reported in the results are comparable and even higher in M-M condition in SkMel28 cells. Please double check analysis.
Figure S8a. MAFG in supposed to be from the same membrane, but the membrane is shown as cropped. Please fix and explain.
Figure S9i. Data shown does not support authors claim. Were the results from this panel repeated n=3? Other experiments are needed to rule out the effect of MAFF and MAFK. (I.e. Could MAFF or MAFK overexpression create oncogenic effect? Could OE of these genes, effect the expression of MITF targets?
Figure S10c. The blot image is very pixelated. Please replace.

Reviewer #2

(Remarks to the Author)
In this study, the authors shown that MAFG interacts with MITF and that MAFG expression modulates melanoma cell proliferation.

The following issues should be addressed.

1. In Figure 1C the p-value for significance is close to the limit, perhaps the authors could analyze separately primary and metastatic samples in the SKCM TCGA, this would perhaps give a better evaluation of the association with outcome especially since MAFG seems to be associated with advanced stage tumours. Concerning the overexpression the authors must show immunoblots on the endogenous and over-expressed MAFG in Fig 1E (move extended data into the main Figures).

2. In Fig 1 FGH please indicate on the Figure which cell line is used and the sample size N= ?

3. In Fig 2 the authors make statements about the tumour histology. This is not at all evident in Fig 2G. These figures should be exchanged with images that clearly sown the histological features that the authors claim, including pigmentation, marco images of tumours, microscopy of regions that the authors claim show increased cellularity or nodular regions. It would also be useful to perform immunoblots or immunostaining to better characterize the expression of known melanoma cell type markers in these tumours such as MITF, SOX10, ZEB1, etc. Does MAFG expression promote melanoma EMT or other cell state transitions in this model in vivo as they suggest for human cell lines. This is important information for the in vivo model that is missing.

4. In Fig 3H and G the authors should quantify the data on MAFG expression. This can be readily done on the Marine-Lab website giving a more accurate quantification of the number of expressing cells and the relative expression levels in the different cell states. You can generate violin plots etc.

5. The experiments shown in Figures 4G and H are somewhat misleading. While they show that MITF is required for the increased proliferation induced by MAFG overexpression, they do not really show that endogenous MITF and MAFG cooperate. It is rather surprising that the WM164 cells are so tolerant of MITF loss. If endogenous MITF is not required for the proliferation of these cells under normal conditions, what is the relevance of the observation that it is required for proliferation under the artificial conditions of MAFG overexpression?

6. The authors hypothesize that MAFG interacts with MITF via the leucine zipper domains (line 205). While the data show co-immunoprecipitation, the authors do not perform any experiments to support the idea that the interactions involve the leucine zipper domains. It could be that MAFG interacts in some other way with MITF homodimers.

7. It is difficult to understand Figure 5A. The authors say they map MAFG and MITF binding sites in the GFP or MAFG overexpressing cells. What is shown in Figure 5A and B, the endogenous MAFG and MITF in the GFP cells or the MAFG overexpressing cells? The authors then claim (lines 246-248) that MAFG overexpression leads to MAFG sites that showed increased or decreased MAFG occupancy. Why does increased MAFG not lead to overall increased binding, and how do they explain that increased expression leads to decreased occupancy. It would be much more appropriate and indeed essential to show read density maps, rather than, or in addition to, the panels in Fig. 5. In particular, Figure 5I is not informative, it is difficult to understand what it is supposed to indicate. When looking at some of the extended Figures for

example 7D, the profile for MITF looks more like published profiles for H3K27ac rather than MITF. For example, how did the authors quantify the number of MITF binding sites at the at the TYR locus where the signal is spread over the entire promoter region?

8 Lines 268-9, the authors investigate the overlap between genes showing altered expression and those with altered MITF/MAFG occupancy. What is the justification for using such a reduced stringency in gene expression with changes of >/< +/- 0.58 rather than the standard Log 2 changes of +/-1? In the end, from all of the data, there are only few solid conclusions on how MAFG overexpression affects MITF binding and gene expression and no real explanation on how such changes impact cell proliferation which is the important readout. The authors should consider that perhaps endogenous MAFG expression is already optimal and they should rather have performed the Cut&Run for MAFG and MITF in cells silenced for MAFG or for MITF. This would have been much more appropriate and in addition provided additional controls for specificity of the Cut&Run signals. It may give more definitive on conclusions on whether MITF is required for MAFG genome occupancy and vice-versa.

9. In the last paragraph of the results, the authors silence MAFG expression in A375 or WM146 cells. In the extended data the authors show immuoblots of A375 cells that seem to strongly express MITF (Ext 4D). This is very surprising since it is considered in the field that A375 cells are of a NCSC-like phenotype expressing SOX10 but not MITF (the authors indeed indicate this transcriptional signature in the text). These results raise several issues. Are the authors sure that they really have A375 cells? Given the high MITF it may be an issue. It would be more appropriate to perform the MAFG silencing in more mesenchymal lines (for example WM793 shown in Ext 4D) that do not express MITF. Are the small MAFs expressed in mesenchymal lines and does their silencing have an effect that may be MITF independent. Furthermore, if MAFG is really partially redundant with the other small MAFs why does MAFG silencing have such a strong effect, would the same been seen upon silencing of the other small MAFs. In any case, the loss of function experiments are amongst the most critical in the manuscript and it is surprising that they are presented at the end of the results section.

Reviewer #3

(Remarks to the Author)
The authors in this manuscript investigate the role of the MAFG-MITF complex in phenotype switching in melanoma and its ability to promote progression. MITF is an essential transcription factor involved in melanocyte differentiation and melanoma but is inherently complex and its role is still not fully understood, particularly given its function in the phenotype switch. This paper proposes that MITF-MAFG interaction is crucial for expression of the expression of many MITF target genes through co-binding of half of their genomic sites, MAFG driven switching towards a de-differentiated state requires MITF interaction, and MAFG is also necessary for cell proliferation and tumor initiation in genetic mouse models.

This paper is very well written, is easy to follow and makes logical sense. Importantly, the experiments are fundamentally sound and they have gone to great efforts to use a number of cell lines and in vivo models to justify their hypothesis, including many complimentary techniques such as RNA-sequencing, CUT&RUN, Proximity Ligation Assay, Co-immunoprecipitation, and their in vivo genetic models. The results are important for the field, particularly given many misconceptions and complexities surrounding the role of MITF in the phenotype switch. Overall, the authors have done a great job and there are no real experiments that need to be added to improve the manuscript. These are some minor suggestions, which once completed, makes the paper suitable for publication. Congratulations to the authors for a very thorough paper:

While the authors provide quantification for Fig 1B, they should include an IHC example for each group.

The authors should include images of FOCI in Figures 4D and 4E.

The discussion should go into a bit more detail about the dedifferentiated state for the reader. The results also show that AXL expression is regulated during MAFG overexpression. The authors should discuss how it occurs given AXLs importance in the phenotype switch. Is this hypothesized to be an MITF-MAFG driven even or is it more indirect.

Finally given the important role of immune checkpoint therapy, a few sentences about MAFG and its role in potential immune related events (immunoevasion, suppression etc) and examples in the literature would be good for context in the discussion.

Reviewer #4

(Remarks to the Author)
I co-reviewed this manuscript with one of the reviewers who provided the listed reports. This is part of the Nature Communications initiative to facilitate training in peer review and to provide appropriate recognition for Early Career Researchers who co-review manuscripts.

Version 1:

Reviewer comments:

Reviewer #1

(Remarks to the Author)
Authors have addressed the reviewers comments and suggestions and the current version of the manuscript is significantly improved.

Reviewer #2

(Remarks to the Author)
This new version of the paper is vastly improved compared to the previous version. The authors have made a major effort to address the issues raised by this referee and have in large part done so effectively.
There are however still some outstanding issues that should be addressed.

1. In the rebuttal, the authors state that they evaluated the expression of SOX2 and AXL in the mouse tumours. This data is not however shown in the revised version of the paper. The H&E staining and the other shown data are not very informative and do not really address the question of phenotype switch in vivo in the mouse model, whose description remains superficial and incomplete. I would encourage the authors to show the data (why SOX2 and not SOX10 or MITF as requested?) it would strengthen the conclusions. Does MAFG promote loss of MITF expression that disappears as the tumours progress and lose their pigmentation
2. The authors have completely revisited the Cut&Run data that is now better presented with clearer conclusions. Now that the issue is better framed it raises the question of specificity of the MAFG specific vs MITF specific vs shared peaks. The authors should investigate the DNA sequences associated with each class of binding site. Does the MITF-MAFG heterodimer recognize preferentially the same sequence motif as the MITF homodimer, is there a motif specificity code that dictates which sites are bound by the different dimers. For example, perhaps fully palindromic M-boxes are more specific to MITF homodimers, whereas more variable E boxes and preferred by the MITF-MAFG heterodimer.
3. Concerning the MITF-MAFG interaction, I would suggest that the authors show the Alpha fold 3 model in the extended data and they move the IP experiment currently shown as Extended 6C to the main figure. The conclusion concerning the importance of the Leucine zipper in the interaction is important as it should lead to the contribution of MAFG to binding the specificity as mentioned above.
4. There seems to be a discrepancy in the results of Fig 3I and J. In panel J MAFG show strong (almost strongest) expression in the RNA processing (and stress-like) population, but this is not represented in panel I. Could the authors please comment.

Reviewer #3

(Remarks to the Author)
The reviewers have addressed all comments and I recommend publication. Congratulations!

Reviewer #4

(Remarks to the Author)
I co-reviewed this manuscript with one of the reviewers who provided the listed reports. This is part of the Nature Communications initiative to facilitate training in peer review and to provide appropriate recognition for Early Career Researchers who co-review manuscripts.

Version 2:

Reviewer comments:

Reviewer #2

(Remarks to the Author)
The authors have now addressed my remaining issues. The finding that the MITF-MAFG-bound sites have a different recognition sequence than the MITF homodimer sites is very interesting and, given the role of AP1 in mesenchymal melanoma cells, intriguing. The authors may consider mentioning in the discussion that if MAFG can interact with MITF and alter its function, what about TFEB and TFE3? This would be an interesting point to consider for the future.

Open Access This Peer Review File is licensed under a Creative Commons Attribution 4.0 International License, which permits use, sharing, adaptation, distribution and reproduction in any medium or format, as long as you give appropriate credit to the original author(s) and the source, provide a link to the Creative Commons license, and indicate if changes were made.
In cases where reviewers are anonymous, credit should be given to 'Anonymous Referee' and the source.
The images or other third party material in this Peer Review File are included in the article's Creative Commons license, unless indicated otherwise in a credit line to the material. If material is not included in the article's Creative Commons license and your intended use is not permitted by statutory regulation or exceeds the permitted use, you will need to obtain permission directly from the copyright holder.
To view a copy of this license, visit https://creativecommons.org/licenses/by/4.0/

We thank the reviewers and editors for their constructive feedback, which has significantly improved our manuscript. In response, we have added new experiments, clarified key interpretations, and expanded our discussion to address all major concerns. Specifically, we performed additional immunoblots, CUT&RUN analyses in silenced cells, and histological characterizations in mouse models. We also refined our data presentation, included missing controls, and contextualized our findings within immune-related mechanisms and phenotype switching. We believe these revisions substantially strengthen the manuscript and address all reviewer comments in full.

Reviewer #1 (Remarks to the Author): Expertise in mouse model, melanoma and MITF

In this manuscript Olga Vera et. al. study the role of the transcription factor MAFG in the control of MITF along its renowned role in phenotype switching, which has been implicated in melanoma progression. Authors suggest that the complex formed between MAFG and MITF occupies >50% of MITF targets in the genome, which attenuates MITF ability to activate these genes transcription. Manipulation of MAFG revealed that it is sufficient to drive a dediffernetiated cell state and accelerate melanoma progression, which is MITF dependent. Authors further show that MAFG is important for melanoma proliferation and progression from nevi to melanoma state in genetic models. In as much authors uncover an important regulator of MITF function along melanoma development and progression. In patients, MAFG expression is elevated and associated with worse survival in melanoma, confirming its relevance for human melanoma. While of interest, the presented manuscript appears to be premature, as it lacks important controls, number of claims are not supported by data shown, and quality of data, at times, is subpar.

We thank Reviewer #1 for their detailed and critical evaluation. In response, we clarified cell line selection, added missing controls and molecular weight markers, and performed new co-immunoprecipitation experiments to assess the role of MAFG phosphorylation. We expanded our analysis of MITF isoforms and included additional data on MAFF and MAFK. We also revised figures and legends for clarity and addressed concerns about data interpretation and experimental design.

General points
why are the WM164 cells less sensitive to MITF silencing compared to SKMel28? Authors claim reduced proliferation and foci formation, but relative to SKMel28, the reduction is not as clear.

It is currently not known why WM164 cells tolerate silencing of MITF when most melanoma cell lines that express MITF do not tolerate its silencing. While this may reflect an intrinsic biological difference between WM164 and SKMel28 cells (which is to be expected between cell lines), it provided the opportunity to test in WM164 cells if the oncogenic effects of MAFG are dependent on MITF. Importantly, silencing of MITF partially rescues the effect of MAFG on proliferation and focus formation in WM164 (Fig. 4g,h), demonstrating that MITF is important but not the only mediator of the MAFG effects. While the same conclusions cannot be drawn from SKMel28 cells because MITF silencing reduces growth of GFP control cells (i.e., MITF is required beyond mediating MAFG's oncogenic effects, Ext Data Fig. 10b,c), this experiment demonstrated that MAFG does not simply bind to and impair MITF function since MITF silencing and MAFG overexpression elicit opposite phenotypes.

Considering MAFG is phosphorylated by ERK for stability, does this phosphorylation promotes

the dimerization of this protein with MIFT? Is phosphorylation necessary for dimerization to take place? Is the overexpressed MAFG phosphorylated (stable) or the effect is due to abundance?

To address the reviewer's question, we expressed wild-type MAFG (MAFG^{WT}), a mutant that cannot be phosphorylated by ERK (MAFG^{S124A}), and a phospho-mimetic mutant (MAFG^{S124D}) in WM164 cells, followed by immunoprecipitation of flag-tagged MAFG and immunoblotting with a MITF and a phospho-Serine/Threonine antibody. Our results demonstrate that phosphorylation of MAFG at Ser124 does not influence the interaction with MITF. Additionally, blotting with the pS/T antibody showed that the ectopically expressed MAFG protein is readily phosphorylated, which is reduced by the S124A mutation. Interestingly, while pS/T typically do not detect phosphomimetic mutants, we found phosphorylation of the MAFG^{S124D} protein. This suggests that either the pS/T antibody detects the phosphomimetic mutant in this case or that MAFG is phosphorylated at additional Ser/Thr residues and phosphorylation at S124 is required for the additional PTMs given an overall reduction in pS/T signal of the S124A mutant. We have added this below results in Ext. Data. Fig. 6d.

MITF has multiple isoforms (also seen in the data presented). Does MAFG interact with any specific isoforms of MITF?

Melanocytes and melanoma cells almost exclusively express the M-isoform of MITF (PMID: 15715979). We interrogated our RNA-seq data for MITF isoform expression in WM164 and SKMel28 melanoma cell lines and found no evidence of transcription for exons 1A, 1H, 1D, and 1B (see Figure R1). Zooming into the genomic location of exon 1M and exon 2 confirmed RNA-seq read alignment with exon 1M (Figure R2), which encodes the N-terminus of the M-MITF isoform (ENST00000394351.3), indicating that M-MITF is the predominant isoform expressed in these melanoma cells. Thus, the two bands observed in MITF western blots from melanoma cells likely represent posttranslational modifications of the M-MITF isoform.

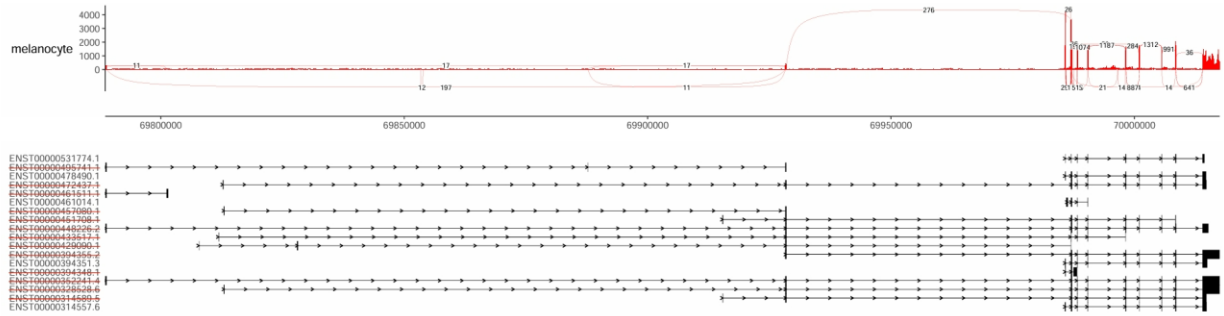

Figure R1: Sashimi plot showing reads from WM164 and SKMel28 cells mapped to the human MITF genomic locus.

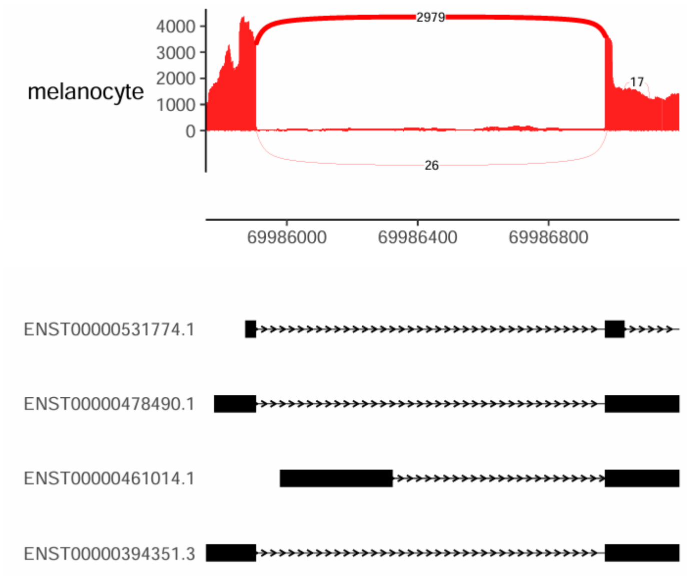

Figure R2: Sashimi plot showing reads from WM164 and SKMel28 cells mapped to the exons 1M and 2 of the human MITF genomic locus.

Additionally, as can be seen in Main Figures 4b, 4c, Ext. Data Fig. 13j, and the results from the phospho-mutant experiment above, MAFG immunoprecipitation pulled down both detectable MITF protein bands. This suggests that MAFG interacts with M-MITF independent of PTMs of the latter.

Multiple cell lines are used throughout a project but for certain assays only one is used. It is not clear why certain assays are done with one cell line rather than another? (for example, the CUT&RUN assay was only used in WM164, or in assessing other components (MAFF and MAFK) only one cell line is assessed) Why was WM164 selected?

Our initial experiments were conducted in both WM164 and SKMel28, two melanoma cell lines characterized by low endogenous levels of MAFG (as previously reported in our miR-29 study, Vera et al., Cancers, 2021). Among these, WM164 consistently exhibited a more pronounced phenotype in response to MAFG modulation, which guided our decision to prioritize this line for resource- and cost-intensive assays such as CUT&RUN. Importantly, however, the MAFG/MITF gene signature derived by integrating CUT&Run with RNAseq was applied to and confirmed in scRNAseq and TCGA datasets (Figure 5), demonstrating that even though a single cell line was used for the CUT&RUN assays, the findings are broadly applicable.

Regarding the assessment of MAFF and MAFK, we agree that including data from both cell lines would strengthen the conclusions. Therefore, we have incorporated new data from SKMel28 in the revised manuscript (Extended Data Figure 13) which show that MAFF and MAFK can bind MITF albeit to a lesser extent, and that these two sMAF family members also have moderate oncogenic effects, corroborating our findings in WM164 cells. All other experiments have been performed in at least two cell lines.

Specific points

Figure 1a. How is the expression of MAFG different in transformed tissues, compared to normal skin? Data set GSE3189 provides 7 samples of normal skin. It would be interesting to demonstrate the relative amounts.

We appreciate the reviewer's suggestion regarding the comparison between melanoma samples and normal skin. However, we respectfully note that such a comparison is not appropriate due to the biological disparity and cellular composition in melanoma vs normal skin. Tumor samples are enriched for melanoma cells; normal skin, by contrast, comprises a heterogeneous mix of cell types – including keratinocytes, fibroblasts, and melanocytes – with only a small proportion of non-transformed melanocytes (typically <5%). This imbalance introduces confounding variables and risks, confusing tumor-specific changes with differences inherent to tissue composition. We hope this clarifies our rationale and respectfully suggest that comparisons to normal skin are not suitable when assessing expression changes in melanoma specimens.

Figure 1b. please add representative images.

We have added representative images of TMA cores representing nevus, primary melanoma, and metastatic melanoma in the revised manuscript in Extended Data Figure 1b.

Figure S1b. How large is the shift in Ectopic MAFG? Please label the membranes with MW markers. How is the basal level of MAFG protein different among these cell lines? Are the exposures displayed in images comparable? It seems like the OE of MAFG had different efficiencies in different cell lines. How did the authors account for that in the follow-up experiment?

We appreciate the reviewer's recommendation and have updated and moved Figure S1b to Main Figure 1, included molecular weight markers, and standardized exposure across all three cell lines. We have re-run the Western blot with the Hermes1, WM164, and SKMel28 cells run side-by-side for comparison of endogenous and ectopic MAFG expression (now shown in Figure 1d including MW marker). Endogenous MAFG (~20 kDa) is expressed at lower levels in control (GFP) Hermes1 cells compared to SKMel28 and WM164 cells, consistent with our previous findings (Vera et al, 2021). The observed shift in ectopic MAFG corresponds to the addition of a Myc/Flag-tag in the expression plasmid (Origene: RC221486), which adds approximately 3 kDa to the native MAFG protein. This tag was used to facilitate detection and was essential for downstream applications such as co-immunoprecipitation.

Figure 1d. First panel - isn't the y-axis supposed to indicate confluency (%) rather than absorbance?

The y-axis in this Figure (Fig. 1e of the revised Figures) showing proliferation of Hermes1 cells is labeled correctly as Absorbance as we used Cystal Violet staining, not confluency percentages, for melanocyte cell lines. As noted in the Methods section, melanocytes were assessed using this traditional staining approach due to technical limitations with the CellCyte system.

Specifically, melanocytes are a slow-growing cell line and the CellCyte equipment could not accommodate the extended 8-day incubation required to capture their proliferation accurately. Additionally, melanocytes require a higher CO_2 concentration (10%) than other cell lines, which posed logistical challenges in adjusting the incubator settings exclusively for these assays. For these reasons, we opted for Crystal Violet staining, which provides a reliable proxy for cell density via absorbance measurements. Importantly, this (and all other assays) are internally controlled as the GFP and MAFG expressing cells are assayed under the same conditions.

Figure 2b. The figure presented does not represent "accelerated melanoma development", rather it's another way to shows survival, which is not much different from data shown in Figure 2c. It would be interesting to know if there are differences in tumor sizes.

We respectfully disagree with the reviewer's statement. The "Tumor Free Survival" analysis shown in Figure 2b shows when tumors first emerge. It is therefore a measure of tumor latency and is not indicative of when mice reach IACUC-mandated endpoints. This analysis therefore captures the latency period between induction and tumor onset. In spontaneous melanoma models, shortened tumor-free survival (i.e., shortened latency) is widely considered indicative of aggressive cancer phenotypes, driven by strong endogenous tumor-promoting mechanisms. Therefore, this analysis provides evidence that MAFG overexpression accelerates the formation of melanomas. To avoid any confusion, we have revised the text to state that MAFG "reduced tumor latency".

Regarding tumor size, we agree this is an important parameter. However, there is an important nuance to tumor size analyses. In xenograft experiments with more uniform tumor growth, all cohorts are collected at a pre-determined time-point and tumor sizes can be compared. Tumor emergence and growth rates are vastly more variable in genetic models of cancer such as the one shown in Fig. 2. Moreover, in the survival analysis shown in Fig. 2, the endpoint is defined by tumor size and, thus, all tumors from different cohorts are the same size at endpoint. What would be more appropriate is to measure the volumes of individual tumors over time. While we have not specifically performed tumor size measurements, we have used a surrogate measurement of tumor growth rate: we measured the time it takes from the emergence of a tumor to when a mouse must be euthanized due to the size of the tumor (based on our IACUC approved tumor volumes). This analysis, shown in Fig. 2e, demonstrates that the "Days to endpoint" measure is shorter in MAFG tumors, indicating that MAFG overexpressing tumors grow faster.

Figure 2g. Please replace MAFG-GFP panel. Image seems to be out of focus or has been filtered. Also, according to the manuscript, Figure 2g is supposed to show "nodular regions" hence, "increased cellularity". The data presented does not match, since the data shows quantification of overall MAFG positive cells by IHC.

The original Figure 2g contained both H&E staining as well as MAFG IHC. We have separated H&E (Fig. 2g) and MAFG (Fig. 2h) to avoid any confusion and have updated the figure legend to better reflect the nature of the image and its relevance to the described pathology. We have replaced the original H&E images with ones that better represent the assessment of our dermato-pathologist, Dr Ken Tsai (co-author on the paper). We have also included lower magnification H&E images in Ext Data Fig 2a. Importantly, these images were taken by Dr. Tsai and clearly illustrate the nodular regions with increased cellularity, consistent with the description

in the manuscript. We believe these revisions address this reviewer's and reviewer #2's concerns and improve the clarity of the presented data.

Figure 3b. Decrease in MITF is not noticeable specially in SKMel28 cells. Also, please add the WB, confirming overexpression of MAFG in these cell lines.

We agree that a clear downregulation of MITF protein is not evident in WM164 and SKMel28 cells, and we were trying to make the point that MAFG does not significantly reduce MITF protein levels. Rather than suggesting a reduction in MITF levels, we show that MAFG overexpression modulates the transcriptional activity of MITF through direct interaction rather than suppression of MITF expression. We acknowledge that our description could be misinterpreted, and we have adjusted the text accordingly. Additionally, we have now included the Western blot confirming MAFG overexpression in all cell lines in Figure 3b.

Figure 3h and i. In the legend, it is mentioned "MAFG expression (left) in human/mouse melanoma cell clusters represent different phenotypic states (right)", however the panel is in the opposite order.

We thank the reviewer for highlighting this labeling mistake. We have edited the figure legend of Figure 3 to correctly describe the panels.

Figure S4d. Please label WB (MITF antibody recognizes different isoforms-which would be something to attend to in assessing MAFG-MITF interactions.

We have added molecular weight markers throughout the manuscript figures as suggested by the reviewer. Please see our response regarding MITF isoforms above.

Figure S4g. Figure legend does not match the panel. Data suggested in the legend are the controls needed for panel 3b, which are not provided.

We thank the reviewer for catching this mistake. We have corrected the legend of Ext Data Fig 4g (now Ext Data Fig 5d) to correctly refer to A375 and WM793 cells. Additionally, MAFG Western blot was added to Figure 3b, as mentioned above.

Figure 4b. The input for the MITF pulldown in SkMel28 cells is not clear. Please provide a more exposed membrane.

We have added a longer exposure of this Western blot to better show input MITF levels.

Figure S5c and 4d. The number of PLA foci in the 3 cell lines are significantly different. How is this biologically relevant between these 3 cell lines? Or perhaps is this difference due to transfection efficiency of MAFG. From previous controls, it appears that not all cells overexpress MAFG with the same efficiency (i.e. S1b, S4g). As a control, authors could include an IF of the overall levels of MAFG, MITF and GFP in these cell lines in addition to the PLA images to see if the basal or overexpressed levels of these proteins is comparable prior to PLA assay. Also, DAPI staining is overexposed in all images (except WM164-MAFG). Please replace with less exposed

images. Does overexpression of MAFG effect the localization of this protein? There are changes in the cytosolic pool of these proteins, that could be interesting to follow.

The differences in PLA foci numbers observed across the three cell lines likely reflect variations in basal MITF (highest in WM164, high in SKMel28, low in A375) expression rather than differences in transfection efficiencies. The cell lines used in this study were generated via lentiviral transduction followed by antibiotic selection, resulting in stable populations expressing either MAFG or GFP, overexpression is comparable across cell lines (see also Figure 1d). As suggested by the reviewer, we performed immunofluorescence (IF) staining for MAFG and MITF in control (GFP) and MAFG-overexpressing cells to further compare basal and ectopic expression levels. This analysis confirmed that (i) MAFG is low in WM164, SKMel28, and A375 GFP control cells and elevated in MAFG overexpressing cells; (ii) MAFG is not redistributed upon overexpression, remaining localized to the nucleus; (iii) WM164 and SKMel28 cells express high MITF while it is undetectable in A375 cells, and (iv) MITF does not change in abundance or localization upon MAFG overexpression across the cell lines. These IF analyses were added as Ext Data Figures 7 and 8.

In response to the reviewer’s concern regarding image quality, we have replaced the overexposed DAPI panels (now Ext Data Fig 9a) with appropriately adjusted images.

Figure 4f. Please include the control blot for MAFG confirming overexpression of the protein.

We have included the Western blot confirming MAFG overexpression in Figure 4f as requested by the reviewer.

Figure 6a. colony formation images do not match the data. Images show significantly higher # of colonies in WM164 cells compared to SkMel28, however, total area reported in the results are comparable and even higher in M-M condition in SkMel28 cells. Please double check analysis.

The quantification that was shown in the original Figure was correct, and we note that the y-axis scales showed different ranges for the two panels (0-80,000 for WM164 and 0-8,000 for SKMel28). Thus, the quantification graphs accurately reflect the images shown. We acknowledge that the values in the y-axes may be difficult to discern. We have revised the axes and replaced the arbitrary units with “percent area covered by foci”, improving clarity and avoiding confusion.

Figure S8a. MAFG in supposed to be from the same membrane, but the membrane is shown as cropped. Please fix and explain.

Endogenous and Myc/DDK-tagged ectopic MAFG (running at 20-25kDa) as well as the tethered MAFG~MITF dimer (running at ~75kDa) shown in this Western blot are indeed from the same membrane. We simply cut the membrane so that we can blot for Actin (~45kDa) and MAFG simultaneously. Importantly, the upper and lower MAFG blots shown are the same exposure (see enclosed uncropped films in rebuttal Figure R3), not only confirming expression of ectopic Myc/DDK-MAFG and MAFG~MITF, but also showing equal abundance of both constructs.

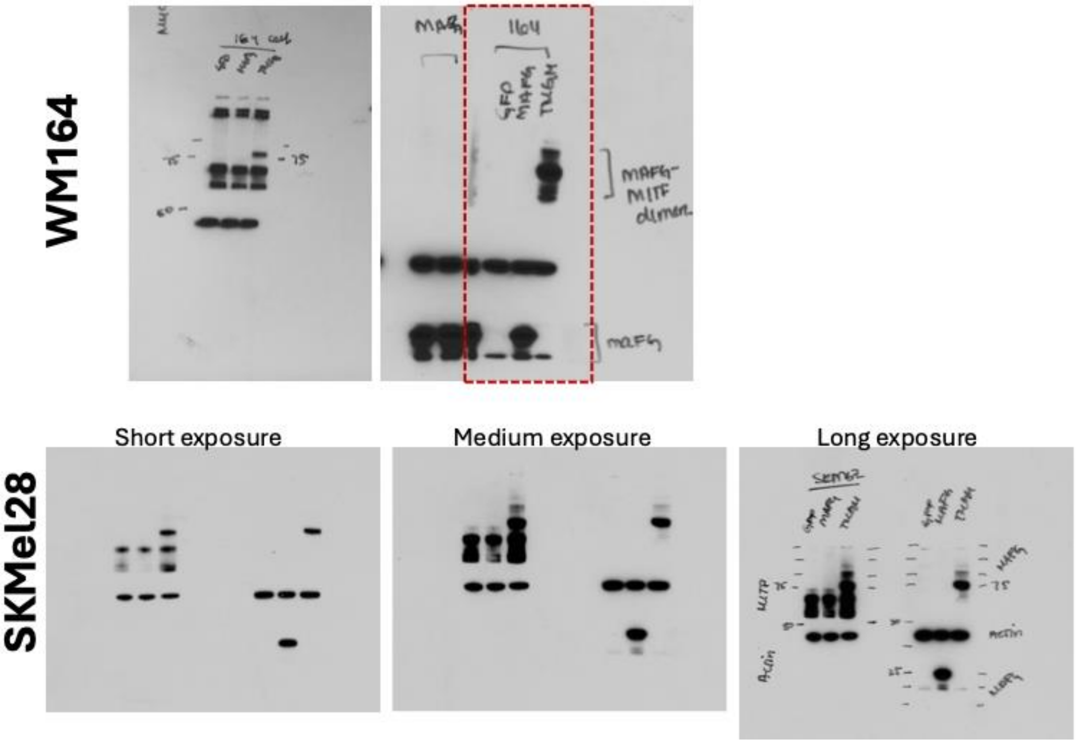

Figure R3: Uncropped films showing that the bands for ectopic MAFG and MAFG~MITF shown in Extended Data Fig. 12a are from the same exposure. The membrane incubated with MAFG antibody was cut so that Actin can be detected simultaneously.

Figure S9i. Data shown does not support authors claim. Were the results from this panel repeated n=3? Other experiments are needed to rule out the effect of MAFF and MAFK. (I.e. Could MAFF or MAFK overexpression create oncogenic effect? Could OE of these genes, effect the expression of MITF targets?

In our original submission, we stated that “These data indicate that all three sMAF proteins have oncogenic effects in melanoma, but MAFG is the most potent family member.” We therefore did not exclude potential contributions of MAFF and MAFK, but rather highlighted the relative strength of MAFG’s effects based on the data presented. We acknowledge that the co-IP Western blot in original Ext Data Fig 9i was not of optimal quality to convincingly show that MAFG binds MITF more efficiently than MAFF and MAFK. We have re-run this Western blot and show images of higher quality, we have incorporated parallel experiments in SKMel28 cells, and show the average (n=3 experiments) quantification of MITF co-immunoprecipitation normalized to sMAF protein pulldown (via Myc Western blot as the three sMAF proteins harbor the same MYC/DDK tag). Moreover, we show that (i) MAFF and MAFK increase proliferation of WM164 and SKMel28 cells as potently as MAFG; (ii) MAFF and MAFK enhance focus formation of WM164 and SKMel28 cells albeit to a lesser extent than MAFG; (iii) MAFF and MAFK induce AXL expression similar to MAFG. These data, now included in Ext Data Fig 13, corroborate our earlier findings and claims. While these data demonstrate oncogenic potential of all three sMAF family members in WM164 and SKMel28 cells, MAFG consistently showed the strongest phenotype. This is likely explained by MAFG displaying the strongest association with MITF; however, we cannot rule out

additional functions of MAFG not shared with MAFF and MAFK that synergize with its effect on MITF to facilitate melanoma progression.

Figure S10c. The blot image is very pixelated. Please replace.

We believe that the pixelation occurred during the conversion to PDF. We have replaced this image with a higher resolution version, which on our end appears clear and of sufficient resolution, and confirmed that it maintains high quality after PDF conversion.

Reviewer #2 (Remarks to the Author): Expertise in melanoma, mouse models, MITF, scRNA-seq and ChIP-seq

In this study, the authors shown that MAFG interacts with MITF and that MAFG expression modulates melanoma cell proliferation.

The following issues should be addressed.

We appreciate Reviewer #2's insightful suggestions. We reanalyzed TCGA data with stratification by tumor stage and origin, added immunoblots for MAFG expression, and improved figure annotations. We included new histological images and marker analyses to better characterize tumor phenotypes and cell state transitions. Additional CUT&RUN experiments in silenced cells were performed, and we clarified the cooperative role of MAFG and MITF in transcriptional regulation and proliferation.

1. In Figure 1C the p-value for significance is close to the limit, perhaps the authors could analyze separately primary and metastatic samples in the SKCM TCGA, this would perhaps give a better evaluation of the association with outcome especially since MAFG seems to be associated with advanced stage tumours. Concerning the overexpression the authors must show immunoblots on the endogenous and over-expressed MAFG in Fig 1E (move extended data into the main Figures).

We thank the reviewer for this suggestion regarding stratification of SKCM TCGA samples by primary versus metastatic origin, particularly in light of the gradually increased expression of MAFG in advanced-stage tumors. We analyzed the dataset by separating primary and metastatic samples. Within the metastatic cohort, high MAFG expression was significantly associated with poorer overall survival ($p = 0.0304$), reinforcing its relevance in disease progression. While there was no association of high MAFG levels with poorer survival in the primary tumor group, this group paradoxically exhibited worse overall survival compared to the metastatic group. This may reflect annotation biases and suggests that this stratification of patients in the SKCM TCGA dataset is not appropriate.

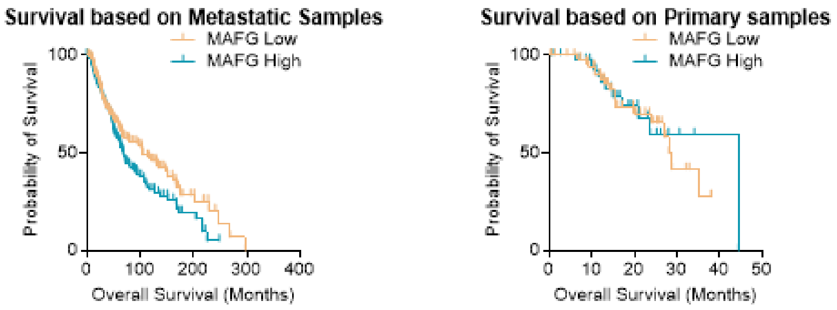

Figure R4: Association of MAFG levels with patient survival in the primary melanoma vs metastatic melanoma cohorts.

To further explore this association, we also stratified patients by tumor stage (I–II, III, and IV). While there was a trend observed in stage IV patients of high MAFG levels being associated with poorer survival, the low number of stage IV specimen limits the conclusions that can be drawn from this analysis.

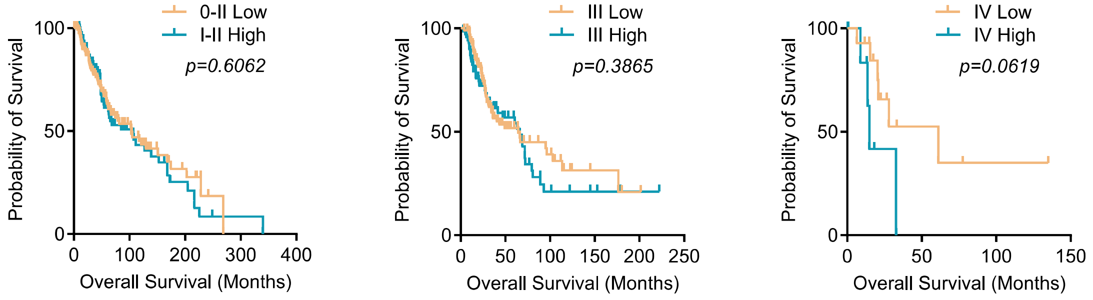

Figure R5: Association of MAFG levels with patient survival in patient cohorts separated by tumor stage.

We additionally tested multiple stratification approaches (including percentile-based thresholds and ROC-survival-based cut-off finder tools) and found that mean-based stratification consistently provided the most robust separation between groups. We have replaced the previous survival analysis with the mean-based stratification in Figure 1. These analyses collectively support the observation that the survival association is statistically significant, and there is a trend toward statistical significance particularly in advanced disease.

Importantly, our mouse modeling data demonstrate that MAFG overexpression cooperates with different oncogenic driver contexts to shorten melanoma latency, increase tumor number, and accelerate melanoma growth. Moreover, MAFG depletion in the aggressive $BRAF^{V600E}$; $PTEN^{-/-}$ model blocked the transition from benign nevi to frank melanoma. The findings

suggest that MAFG likely plays roles at different stages of melanomagenesis. Thus, while MAFG levels increase with melanoma progression, the stratification of patients based on metastasis status or tumor stage for survival analysis may not capture the entirety of MAFG effects in melanomagenesis.

We have now incorporated immunoblot data directly into the main figures (Figure 1d) to include panels showing both endogenous and ectopically overexpressed MAFG, as suggested by the reviewer.

2. In Fig 1 FGH please indicate on the Figure which cell line is used and the sample size N= ?

We have revised Figure 1f-i to indicate both the cell line used and the corresponding sample size.

3. In Fig 2 the authors make statements about the tumour histology. This is not at all evident in Fig 2G. These figures should be exchanged with images that clearly sown the histological features that the authors claim, including pigmentation, marco images of tumours, microscopy of regions that the authors claim show increased cellularity or nodular regions. It would also be useful to perform immunoblots or immunostaining to better characterize the expression of known melanoma cell type markers in these tumours such as MITF, SOX10, ZEB1, etc. Does MAFG expression promote melanoma EMT or other cell state transitions in this model in vivo as they suggest for human cell lines. This is important information for the in vivo model that is missing.

We have replaced the original H&E images with ones that better represent the assessment of our dermato-pathologist, Dr Ken Tsai (co-author on the paper). Importantly, these images were taken by Dr. Tsai and clearly illustrate the nodular regions with increased cellularity, consistent with the description in the manuscript. We have also included lower magnification images in Extended Data Figure 2.

To address the reviewer's question about cell state transitions in the mouse models, we performed IHC for SOX2 and AXL on MAFG overexpressing and GFP control tumors (n=5 tumors per group). These markers are expressed at high levels in stem cell-like, mesenchymal, and neural crest stem cell-like states and are low in the melanocytic state. We found that the expression of both markers is increased in MAFG tumors, corroborating our human cell line observation that MAFG promotes a phenotype switch from the melanocytic to neural crest and mesenchymal like states.

4. In Fig 3H and G the authors should quantify the data on MAFG expression. This can be readily done on the Marine-Lab website giving a more accurate quantification of the number of expressing cells and the relative expression levels in the different cell states. You can generate violin plots etc.

The dot plots shown in Fig. 3g and 3i are showing MAFG expression levels and number of cells expressing MAFG in the Marine scRNAseq datasets for human and mouse melanoma. We acknowledge that the way we plotted the data is not intuitive and we have revised the dot plots to make the data clearer. We show these plots in lieu of violin plots as the latter do not capture the percent of cells expressing MAFG. These plots show that MAFG expression is lowest

in the melanocytic cell state in both human and mouse melanoma, and we hope that these revised dot plots alleviate the reviewer's concern.

5. The experiments shown in Figures 4G and H are somewhat misleading. While they show that MITF is required for the increased proliferation induced by MAFG overexpression, they do not really show that endogenous MITF and MAFG cooperate. It is rather surprising that the WM164 cells are so tolerant of MITF loss. If endogenous MITF is not required for the proliferation of these cells under normal conditions, what is the relevance of the observation that it is required for proliferation under the artificial conditions of MAFG overexpression?

We thank the reviewer for this thoughtful comment. We agree that the tolerance of WM164 cells to MITF silencing is surprising, but this provided the opportunity to test whether MITF is specifically required for the oncogenic effects of MAFG. Our data indicate that while under basal conditions MITF is dispensable for proliferation, it is required, at least in part, for the oncogenic effects of MAFG. These data point to a context-dependent functional cooperation between the two factors where MITF mediates the effects of MAFG.

MAFG does not simply impair the activity of MITF, since MITF silencing in parental WM164 and SKMel28 cells does not phenocopy the effects of MAFG overexpression. Moreover, our tethered MAFG~MITF experiments demonstrate that the dimer induces transcriptional changes that overlap with MAFG overexpression, indicating that MAFG engages MITF to reprogram transcription. Importantly, the critical role for MITF is further demonstrated by the lack of an effect of MAFG overexpression in melanoma cells with low/absent MITF such as A375 and WM793, implying that the proliferative effect observed upon MAFG overexpression is not solely due to MAFG activity but also requires transcriptional engagement via MITF. Furthermore, we have shown that endogenous MAFG and MITF physically interact, as evidenced by reciprocal co-immunoprecipitation experiments (Fig. 4c), supporting the notion that this interaction occurs under physiological conditions. Therefore, silencing MITF in WM164 cells disrupts this cooperative transcriptional output, which cannot be compensated by MAFG alone.

We have revised our Discussion to further contextualize the functional interaction of MAFG with MITF.

6. The authors hypothesize that MAFG interacts with MITF via the leucine zipper domains (line 205). While the data show co-immunoprecipitation, the authors do not perform any experiments to support the idea that the interactions involve the leucine zipper domains. It could be that MAFG interacts in some other way with MITF homodimers.

We agree with the reviewer that our statement was speculative. To address this, we used AlphaFold3 to model the MAFG~MITF dimer. This approach predicted interaction of the respective leucine zippers and indicated hydrogen bond pairing between the two zippers that recapitulate the interaction of MAFG with NRF2. Met100, Leu104, and Leu107 of MAFG appear to tightly interlock with the partner Leucine Zipper in both NRF2 and MITF, and this interaction with MITF appeared to involve Leu281 and Ile285. While the predicted interaction extends beyond these amino acids, we mutated M100/L104/L107 in MAFG and L281/I285 in MITF and performed co-immunoprecipitation experiments in HEK293 cells. This demonstrated that the mutations in the respective Leucine Zippers completely abrogated the interaction of MAFG with MITF, indicating that the interaction indeed occurs via the Zipper domains. We have included this experiment in Ext Data Fig 6c.

7. It is difficult to understand Figure 5A. The authors say they map MAFG and MITF binding sites in the GFP or MAFG overexpressing cells. What is shown in Figure 5A and B, the endogenous MAFG and MITF in the GFP cells or the MAFG overexpressing cells? The authors then claim (lines 246-248) that MAFG overexpression leads to MAFG sites that showed increased or decreased MAFG occupancy. Why does increased MAFG not lead to overall increased binding, and how do they explain that increased expression leads to decreased occupancy. It would be much more appropriate and indeed essential to show read density maps, rather than, or in addition to, the panels in Fig. 5. In particular, Figure 5I is not informative, it is difficult to understand what it is supposed to indicate. When looking at some of the extended Figures for example 7D, the profile for MITF looks more like published profiles for H3K27ac rather than MITF. For example, how did the authors quantify the number of MITF binding sites at the at the TYR locus where the signal is spread over the entire promoter region?

Please see below our combined comments to the reviewer's points 7 and 8.

8 Lines 268-9, the authors investigate the overlap between genes showing altered expression and those with altered MITF/MAFG occupancy. What is the justification for using such a reduced stringency in gene expression with changes of >/< +/- 0.58 rather than the standard Log 2 changes of +/-1? In the end, from all of the data, there are only few solid conclusions on how MAFG overexpression affects MITF binding and gene expression and no real explanation on how such changes impact cell proliferation which is the important readout. The authors should consider that perhaps endogenous MAFG expression is already optimal and they should rather have performed the Cut&Run for MAFG and MITF in cells silenced for MAFG or for MITF. This would have been much more appropriate and in addition provided additional controls for specificity of the Cut&Run signals. It may give more definitive on conclusions on whether MITF is required for MAFG genome occupancy and vice-versa.

We appreciate the reviewer's insightful evaluation of our Cut&Run experiments, which led to the realization that our analyses and data representation was too complex and convoluted. Based on the points raised by the reviewer, we have re-ran Cut&Run with MAFG overexpression and silencing conditions side-by-side and performed analyses that more clearly demonstrate the effect of MAFG on genome occupancy of MITF and the consequential gene expression changes. These new analyses are shown in Figure 5 and include:

1) We show that at baseline, MAFG and MITF display unique and overlapping peaks, and upon MAFG overexpression MITF occupancy shifts from unique peaks to overlapping peaks (Venn diagrams and density maps). This demonstrates that MAFG promotes genome binding of MITF as a MAFG~MITF heterodimer.
2) We integrate differential MAFG and MITF occupancy upon MAFG overexpression (unique and overlapping peaks) with RNA expression changes (Volcano plots). This showed that differential binding of MAFG and/or MITF can both repress and stimulate expression of target genes.
3) We identify differentially expressed genes that also show significant changes in MITF occupancy upon MAFG overexpression (both unique and overlapping peaks). Gene ontology analysis of these genes identified EMT as the major Cancer Hallmark.
4) We then analyzed the expression pattern of these MAFG/MITF-dependent genes in the different melanoma cell states in human and mouse scRNAseq data. Remarkably, the expression strongly correlates (positive regulation of upregulated genes, negative correlation with downregulated genes) with the Mesenchymal-like and Neural Crest-like

cell states. This indicates that MAFG through MITF promotes the expression of genes that are associated with the MAFG-driven phenotype switch.

5) We then analyzed the correlation of the differentially expressed gene sets with MAFG and MITF expression in TCGA-SKCM dataset. MITF expression strongly correlated with the gene sets while MAFG expression did not. This indicates that these genes are tightly controlled by MITF levels and that MAFG dysregulation alone is not sufficient to affect their expression - it requires MITF for this effect.
6) Finally, we asked whether MAFG is needed for MITF genome occupancy. While MAFG silencing showed the expected overall reduction of peak heights, MITF peaks (both unique and overlapping) were only modestly affected and trended toward enhanced occupancy. This finding indicates that MAFG slightly impairs MITF genome occupancy, possibly because a MAFG~MITF dimer has reduced DNA binding affinity compared to a MITF homodimer.

We believe these analyses address all of the reviewer’s points and comprehensively characterize the genome occupancy of MAFG and MITF and the consequential gene expression changes.

9. In the last paragraph of the results, the authors silence MAFG expression in A375 or WM146 cells. In the extended data the authors show immuoblots of A375 cells that seem to strongly express MITF (Ext 4D). This is very surprising since it is considered in the field that A375 cells are of a NCSC-like phenotype expressing SOX10 but not MITF (the authors indeed indicate this transcriptional signature in the text). These results raise several issues. Are the authors sure that they really have A375 cells? Given the high MITF it may be an issue. It would be more appropriate to perform the MAFG silencing in more mesenchymal lines (for example WM793 shown in Ext 4D) that do not express MITF. Are the small MAFs expressed in mesenchymal lines and does their silencing have an effect that may be MITF independent. Furthermore, if MAFG is really partially redundant with the other small MAFs why does MAFG silencing have such a strong effect, would the same been seen upon silencing of the other small MAFs. In any case, the loss of function experiments are amongst the most critical in the manuscript and it is surprising that they are presented at the end of the results section.

We thank the reviewer for this thoughtful comment. We appreciate the opportunity to clarify the apparent discrepancy in MITF expression in A375 cells, the role of small MAFs (sMAFs), and the rationale behind the structure of our manuscript.

Regarding the A375 cell line, we STR footprint all our cell lines regularly and we are certain our A375 cell line is authentic. We acknowledge that the western blot shown in Extended Data Fig. 4D may suggest relatively high MITF expression; however, this is likely due to a long exposure blot being shown. We have replaced the MITF blot in Extended Data Fig. 5a with a lower exposure image, and the difference in MITF levels between A375 and WM164/SKMel28 is now readily apparent. Importantly, multiple additional pieces of evidence corroborate low MITF levels in our A375 cells. RT-qPCR analysis (Extended Data Fig. 5b) confirms low MITF expression and its melanocytic target genes, alongside elevated AXL levels - consistent with a NCSC-like phenotype. This transcriptional profile is further supported by RNA-seq data (Extended Data Fig. 5c), which show downregulation of the melanocytic program. To reinforce this, we have now included immunofluorescence data (Extended Data Fig. 8) showing no detectable MITF protein in A375 cells, validating our previous data through an independent method.

Regarding the silencing of MAFG, as suggested by the reviewer, we extended our analysis to include the mesenchymal-like WM793 cell line as well as SKMel28 cells (Fig. 7). Consistent with our findings in A375 and WM164 cells, MAFG silencing in WM793 and SKMel28 cells significantly impairs cell proliferation, indicating that MAFG is critical across both melanocytic and mesenchymal-like melanoma phenotypes.

To address the question of redundancy among small MAFs, we performed targeted silencing of MAFG, MAFF, and MAFK in both WM164 and A375 cells (Extended Data Fig. 15). Our results show that only MAFG silencing leads to a marked reduction in proliferation and colony formation, while MAFF and MAFK silencing has less robust effects. These findings suggest that MAFG plays a non-redundant and indispensable role in melanoma, despite its structural similarity to other sMAFs.

Intriguingly, the impact of MAFG silencing appears to be independent of MITF status, as it affects both MITF-high and MITF-low cell lines. This further suggests that the oncogenic effect of elevated MAFG is mediated by a different mechanism than the effect of MAFG depletion. This could reflect a difference in dimer composition at basal vs overexpressed MAFG levels. Specifically, at basal levels, MAFG may primarily interact with its canonical partners to which it has high affinity. Silencing of MAFG would impair the function of these canonical dimers. When MAFG is overexpressed, greater abundance of MAFG enables the interaction with lower affinity partners such as MITF, while the canonical dimers are unaffected (or become also more abundant). While we agree that the loss-of-function experiments are important to show the critical role of MAFG in melanomagenesis, the placement of these experiments at the end of the manuscript was chosen to reflect the MITF-independent mechanism underlying MAFG silencing. We have further clarified this distinction in the Discussion.

Reviewer #3 (Remarks to the Author): Expertise in melanoma phenotypic switch, MITF and mouse models

The authors in this manuscript investigate the role of the MAFG-MITF complex in phenotype switching in melanoma and its ability to promote progression. MITF is an essential transcription factor involved in melanocyte differentiation and melanoma but is inherently complex and its role is still not fully understood, particularly given its function in the phenotype switch. This paper proposes that MITF-MAFG interaction is crucial for expression of the expression of many MITF target genes through co-binding of half of their genomic sites, MAFG driven switching towards a de-differentiated state requires MITF interaction, and MAFG is also necessary for cell proliferation and tumor initiation in genetic mouse models.

This paper is very well written, is easy to follow and makes logical sense. Importantly, the experiments are fundamentally sound and they have gone to great efforts to use a number of cell lines and in vivo models to justify their hypothesis, including many complimentary techniques such as RNA-sequencing, CUT&RUN, Proximity Ligation Assay, Co-immunoprecipitation, and their in vivo genetic models. The results are important for the field, particularly given many misconceptions and complexities surrounding the role of MITF in the phenotype switch. Overall, the authors have done a great job and there are no real experiments that need to be added to improve the manuscript. These are some minor suggestions, which once completed, makes the

paper suitable for publication. Congratulations to the authors for a very thorough paper:

We thank Reviewer #3 for their insightful feedback and positive assessment. In response, we have added requested images, clarified the role of AXL in the phenotype switch, and expanded the discussion to include MAFG's potential involvement in immune regulation. We appreciate their suggestions, which helped strengthen the manuscript.

While the authors provide quantification for Fig 1B, they should include an IHC example for each group.

Based on the reviewer's suggestion, we have added representative immunohistochemistry (IHC) images for each group to complement the quantification shown in Figure 1b. These examples are now included in Ext Data Fig 1b to provide visual context for the scoring.

The authors should include images of FOCI in Figures 4D and 4E.

As suggested by the reviewer, we have now included representative images illustrating PLA foci in the revised manuscript, which can be found in Ext Data Fig 9.

The discussion should go into a bit more detail about the dedifferentiated state for the reader. The results also show that AXL expression is regulated during MAFG overexpression. The authors should discuss how it occurs given AXLs importance in the phenotype switch. Is this hypothesized to be an MITF-MAFG driven even or is it more indirect

We thank the reviewer for raising this important point. We have added more detail to our Discussion regarding the dedifferentiated state. Moreover, we included CUT&RUN data that show a peak for MAFG in the AXL gene. This suggests that MAFG may directly regulate the expression of AXL. We also included a statement regarding the potential AXL regulation by MAFG in the Discussion.

Finally given the important role of immune checkpoint therapy, a few sentences about MAFG and its role in potential immune related events (immunoevasion, suppression etc) and examples in the literature would be good for context in the discussion.

We appreciate the reviewer's suggestion to contextualize MAFG within the framework of immune-related mechanisms, particularly in light of the growing relevance of immune checkpoint therapy. While our current study does not directly address immunoevasion or immune modulation, we fully agree that this is a critical and emerging area of investigation — and one that we are actively pursuing as a future direction. Recent literature has begun to uncover compelling links between MAFG and immune regulation across various cancer types:

- In hepatocellular carcinoma (HCC), MAFG overexpression has been shown to promote senescence and stemness, while its inhibition reduces PD-L1 expression and enhances antigen presentation. Notably, MAFG-silenced tumor cells are more susceptible to T cell-mediated cytotoxicity, suggesting a role in immune evasion (PMID: 38614215).
- In breast cancer, MAFG and NRF1 were identified as key regulators of PD-L1 transcription via a super-enhancer locus. Silencing MAFG impaired the ability of tumor cells to escape T cell killing, reinforcing its role in immune suppression (PMID: 37985752).

- In astrocytes, MAFG is activated by GM-CSF and contributes to pro-inflammatory transcriptional programs, suggesting its involvement in shaping the immune microenvironment beyond tumor cells (PMID: 32051591).
- In lung cancer, emerging data suggest that MAFG may influence CD8+ T cell function directly, although the mechanistic details remain to be fully elucidated (PMID: 39235522).

These findings collectively support the hypothesis that MAFG may act as a transcriptional hub linking tumor-intrinsic programs to immune modulation. Although we were not able to experimentally explore this axis in melanoma within the scope of the current study, it remains a central goal of our future research. We are currently developing models to investigate how MAFG may influence immune cell recruitment, checkpoint expression, and response to immunotherapy in melanoma. We have added a brief paragraph in the Discussion to reflect this emerging connection and to acknowledge the potential relevance of MAFG in immune-related pathways.

Reviewer #4 (Remarks to the Author): An Early Career Researcher Co-Reviewer

I co-reviewed this manuscript with one of the reviewers who provided the listed reports. This is part of the Nature Communications initiative to facilitate training in peer review and to provide appropriate recognition for Early Career Researchers who co-review manuscripts.

We are grateful for Reviewer #4’s time and effort dedicated to evaluating our study.

We thank the editor and reviewer 2 for their constructive feedback on our manuscript describing a MAFG-induced phenotype switch that promotes melanoma formation. We have fully addressed the few remaining concerns of reviewer 2, which further strengthened our study significantly. We are grateful for the support and believe our revised manuscript is now a strong candidate for publication in Nature Communications. Please see below how we addressed the remaining reviewer concerns in a point-by-point fashion.

1. In the rebuttal, the authors state that they evaluated the expression of SOX2 and AXL in the mouse tumours. This data is not however shown in the revised version of the paper. The H&E staining and the other shown data are not very informative and do not really address the question of phenotype switch in vivo in the mouse model, whose description remains superficial and incomplete. I would encourage the authors to show the data (why SOX2 and not SOX10 or MITF as requested?) it would strengthen the conclusions. Does MAFG promote loss of MITF expression that disappears as the tumours progress and lose their pigmentation.

We selected SOX2 and AXL as markers of a phenotype switch because their expression is very low in the melanocytic population and high in Stem-like, NC-like, and Mesenchymal-like clusters based on Chris Marine's scRNAseq analysis of murine melanoma. This was included in Extended Data Figure 4 and showed an increase of SOX2 and AXL positive cells in MAFG expressing melanomas. We were hesitant to use SOX10 and MITF as markers because (i) while SOX10 is somewhat higher in NC-like cells, it is more broadly expressed in all populations, (ii) murine melanomas often downregulate expression of the pigmentation genes including MITF, and (iii) SOX10 regulates MITF expression and since MAFG influences MITF activity, it could engage feedback mechanisms that affect SOX10 expression. However, to further characterize the phenotype switch in murine melanomas expressing MAFG, we performed IHC for SOX10 and MITF as requested by the reviewer. We found that MITF expression is low and not affected by MAFG, which corroborates our observations in human melanoma cell lines and suggests that MITF levels are maintained despite a phenotype switch away from the Melanocytic state. This is likely explained by MAFG influencing MITF's transcriptional output, which negates the requirement for MITF downregulation to reach dedifferentiated phenotypic states. In contrast, SOX10 levels are increased in MAFG tumors. As SOX10 levels are highest in neural crest progenitors and lower in terminally differentiated melanocytes, this SOX10 pattern supports dedifferentiation of MAFG expressing melanoma cells, in line with the observed phenotype switch. However, we cannot exclude the possibility that the influence of MAFG on MITF activity induces a feedback mechanism that promotes the upregulation of SOX10. Nevertheless, we think these data further support a MAFG-induced phenotype switch in murine melanomas in vivo. Our future studies will include scRNAseq-based analyses of the dynamics and reversibility of the MAFG-induced phenotype switch; however, these future analyses are beyond the scope of the current study.

2. The authors have completely revisited the Cut&Run data that is now better presented with clearer conclusions. Now that the issue is better framed it raises the question of specificity of the MAFG specific vs MITF specific vs shared peaks. The authors should investigate the DNA sequences associated with each class of binding site. Does the MITF-MAFG heterodimer recognize preferentially the same sequence motif as the MITF homodimer, is there a motif specificity code that dictates which sites are bound by the different dimers. For example, perhaps fully palindromic M-boxes are more specific to MITF homodimers, whereas more variable E boxes and preferred by the MITF-MAFG heterodimer.

This is an excellent suggestion, and we have performed the motif analysis recommended by the reviewer. The results are very intriguing: unique MITF peaks show a strong motif enrichment for E/M-boxes, the canonical binding site for MITF, while unique MAFG peaks show strong enrichment for the canonical MAFG binding site MARE. Interestingly, genomic sites where both MAFG and MITF bind (presumably as

heterodimer) are most strongly enriched for AP-1 motifs that are naturally embedded in the MARE motif, and some weak E-box motifs are also present. Thus, while canonical dimers containing MAFG and MITF strongly associate with their respective binding motifs, the heterodimer binds to weaker motifs that partially resemble MARE (AP-1) and E-box motifs). This explains why the heterodimer affects expression of only a subset of MAFG and MITF target genes and why the tethered MAFG~MITF dimer induces expression changes that only partially overlap with those induced by MAFG alone. These motif enrichment analyses are now included in Figure 5e.

3. Concerning the MITF-MAFG interaction, I would suggest that the authors show the Alpha fold 3 model in the extended data and they move the IP experiment currently shown as Extended 6C to the main figure. The conclusion concerning the importance of the Leucine zipper in the interaction is important as it should lead to the contribution of MAFG to binding the specificity as mentioned above.

As recommended by the reviewer, we have included the AlphaFold3 structure prediction of the MAFG-NRF2 and MAFG-MITF dimers in Extended Data Figure 6 and moved the co-IP of the leucine zipper mutants to main Figure 4d.

4. There seems to be a discrepancy in the results of Fig 3I and J. In panel J MAFG show strong (almost strongest) expression in the RNA processing (and stress-like) population, but this is not represented in panel I. Could the authors please comment.

We thank the reviewer for catching this, there was indeed a mistake in our analysis of MAFG expression in the cell clusters of murine melanoma. We have re-analyzed MAFG expression and are now showing the correct levels in Figure 3i. We also double checked the MAFG expression analysis in human melanoma cell clusters and confirmed this is correct as shown.

Rebuttal

The authors have now addressed my remaining issues. The finding that the MITF-MAFG-bound sites have a different recognition sequence than the MITF homodimer sites is very interesting and, given the role of AP1 in mesenchymal melanoma cells, intriguing. The authors may consider mentioning in the discussion that if MAFG can interact with MITF and alter its function, what about TFEB and TFE3? This would be an interesting point to consider for the future.

We thank the reviewer for their positive evaluation of our work. We have included a statement in the Discussion regarding the potential interaction of MAFG with TFEB and TFE3, as suggested by the reviewer.
